# RSF1‐Dependent PAR Turnover Promotes 53BP1 Liquid Condensate Formation at DNA Damage Sites

**DOI:** 10.1002/advs.77271

**Published:** 2026-08-24

**Authors:** Yungyeong Heo, Yonghyeon Kim, Nangyeong Hwang, Gwanho Sung, Sujin Jo, Yeonhu Lee, Ho‐Chul Kang, Jae‐Ho Lee, Yong Won Choi, Hyeseong Cho, Sunwoo Min

**Affiliations:** ^1^ Department of Biochemistry and Molecular Biology Ajou University School of Medicine Suwon Republic of Korea; ^2^ Department of Biomedical Sciences, The Graduate School Ajou University Suwon Republic of Korea; ^3^ Department of Biochemistry, College of Natural Sciences Chungnam National University Daejeon Republic of Korea; ^4^ Department of Physiology Ajou University School of Medicine Suwon Republic of Korea; ^5^ Department of Hematology and Oncology Ajou University School of Medicine Suwon Republic of Korea; ^6^ Inflamm‐Aging Translational Research Center Ajou University School of Medicine Suwon Republic of Korea

**Keywords:** 53BP1, phase separation, remodeling and spacing factor 1

## Abstract

Liquid–liquid phase separation (LLPS) is essential for proper DNA damage signaling. The sequential formation of PAR‐ and 53BP1‐based condensates must be tightly controlled in space and time, however the molecular regulators of this process remain insufficiently defined. Here, we show that RSF1 contains a substantial proportion of intrinsically disordered regions (IDRs) and participates in the sequential assembly of liquid condensates at DSB sites. RSF1 depletion severely abolishes 53BP1 optoDroplet formation without affecting FUS condensates, but causes persistent PAR chains due to defective PARG recruitment. Domain mapping reveals that the C‐terminal region of the IDR in RSF1 is sufficient to support 53BP1 condensate formation. Mechanistically, RSF1 controls the timely extinction of PAR chains by promoting the recruitment of PARG, but not PARP1, to DNA damage sites. This regulation enables the transition from PAR‐based scaffolds to 53BP1 condensates. Importantly, defects in RSF1‐mediated condensate formation correlate with impaired transcription of p53 target genes, including p21CIP1. Together, these findings establish RSF1 as a molecular coordinator that links PAR turnover to 53BP1 condensate assembly, thereby coupling the spatial and temporal regulation of LLPS with p53‐dependent transcriptional responses at DNA lesions.

## Graphical abstract

At sites of DNA damage, RSF1 recruits PARG to accelerate PAR turnover, triggering a switch from PAR‐driven condensates to 53BP1 condensates. This condensate transition enables p53‐dependent gene transcription and coordinates the DNA damage response. The study identifies RSF1 as a central regulator that couples biomolecular condensate dynamics to efficient genome maintenance during DNA repair.

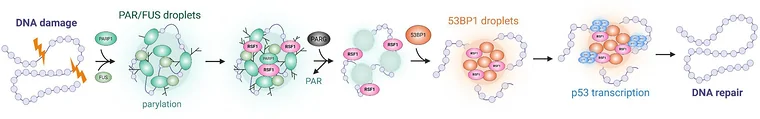

## 1 Introduction

DNA double‐strand breaks (DSBs) represent a major threat to genome integrity [1]. In order to protect the genome, DNA damage response (DDR) is triggered in response to DSBs, resulting in the formation of transient repair compartments [2, 3, 4]. The DDR is initiated by ATM kinase, which phosphorylates the histone variant H2AX to produce γH2AX [5, 6]. The adaptor protein MDC1 then binds to γH2AX, which subsequently recruits the ubiquitin ligases of RNF8 and RNF168 [7, 8, 9, 10, 11]. The ubiquitination at the sites of K13/15 of H2A recruits 53BP1 [12], which cooperates with the REV7/shieldin complex to facilitate the non‐homologous end joining (NHEJ) repair [13, 14, 15]. BRCA1 plays a role in error‐free homologous recombination repair (HRR) [16, 17, 18, 19, 20, 21], in a cell cycle‐dependent manner [22, 23, 24, 25].

Recent studies have revealed that DSB repair involves the formation of dynamic, membrane‐less compartments through liquid‐liquid phase separation (LLPS) [26, 27, 28, 29]. These condensates spatially and temporally concentrate DDR factors to coordinate repair [30, 31]. At damaged chromatin, PAR‐seeded liquid demixing triggers the assembly of FUS, initiating dynamic intracellular compartments for the spatial segregation [32]. In contrast, 53BP1 is excluded from PAR‐initiated condensates [32], which indicates that 53BP1 condensate is a distinct type of protein assembly. The ability of 53BP1 to undergo LLPS depends on its oligomerization domain, and recent evidence suggests that 53BP1‐mediated phase separation is linked to global p53 target gene activation following DNA damage [26, 33]. A defining feature of LLPS‐associated proteins is the presence of intrinsically disordered regions (IDRs), which mediate weak, multivalent interactions required for condensate formation [34, 35, 36]. IDRs are especially enriched in chromatin‐associated and transcriptional regulators‐proteins whose functions often rely on transient interactions and dynamic regulation. Despite growing recognition of LLPS in DDR, the contribution of IDRs within ATP‐dependent chromatin remodeling complexes remains poorly understood [37]. Given the central role of remodelers such as SWI/SNF and ISWI in genome stability, an important unresolved question is whether their disordered regions participate in or regulate the sequential assembly of DNA damage‐induced condensates like those formed by FUS and 53BP1.

Remodeling and Spacing Factor 1 (RSF1) belongs to the ISWI family member of ATP‐dependent chromatin remodeling complexes [38, 39]. Heterodimer of RSF1 and SNF2H forms RSF, which is shown to mediate nucleosome deposition and generates regularly spaced nucleosome arrays in vitro during chromatin assembly and transcription [38, 40, 41]. In cells, RSF1 accumulates at DNA lesions and mitotic centromeres [42]. At DSB sites, RSF1 interacts with ATM and the CENP‐S–CENP‐X centromere proteins to promote DNA repair by HR and NHEJ [43, 44]. RSF1 also interacts with HDAC1 and this interaction promotes transcriptional silencing at DSB sites [45, 46]. In neural‐specific rsf1 knockout mice, apoptosis induced by exogenous DNA strand breaks was reduced. Likewise, expression of p53 target genes was reduced in RSF1 knockout cells, suggesting that RSF1 functions in p53‐mediated global transcription upon DNA damage [47, 48]. At mitotic centromeres, RSF1 interacts HDAC1 and PLK1 and these interactions are crucial for faithful chromosomal segregation [49]. It is shown that N‐terminal alpha helical module of RSF1 interacts with nucleosomal linker DNA and SNF2H [38, 50]. However, a large portion of RSF1 does not fold into a fixed three‐dimensional structure which is a characteristic of IDR.

In this study, we investigated whether RSF1 contributes to the dynamic formation of LLPS‐mediated compartments within the DNA damage response. Our findings indicate that RSF1 is dispensable for initial formation of PAR‐FUS liquid condensates, but it is essential for PAR clearance, likely by facilitating timely recruitment of PARG. Furthermore, RSF1 is a critical component of the 53BP1 liquid condensate, and its presence is tightly linked to the activation of p53 target genes. These findings suggest that RSF1, through its disordered regions, functions as a regulatory mediator of the dynamic DDR condensate landscape during DNA damage response.

## 2 Results

### 2.1 RSF1 Facilitates the Formation of 53BP1 Liquid Condensate but Not FUS Droplet

We first addressed the role of RSF1 in the formation of biomolecular condensates at damaged chromatin by analyzing the biochemical feature of RSF1 protein. AlphaFold Protein Structure Database (EMBL‐EBI) and PONDR Score analyses revealed that only N‐terminal region of RSF1 contains the folded structure while other 80% is intrinsically disordered region (IDR) (Figure 1A and Figure S1A). Given the established role of IDRs in driving liquid–liquid phase separation (LLPS) in both DNA damage [34, 35] and transcriptional compartments [51], we next examined whether RSF1 holds the capability to induce liquid condensates by itself. To assess this possibility, we generated RSF1–mCherry–Cry2 fusion constructs and evaluated their ability to undergo light‐induced optoDroplet formation upon 488 nm illumination [26, 52, 53]. Cry2 is used to promote the oligomerization of the fusion protein that induces optoDroplet formation.

**FIGURE 1:**
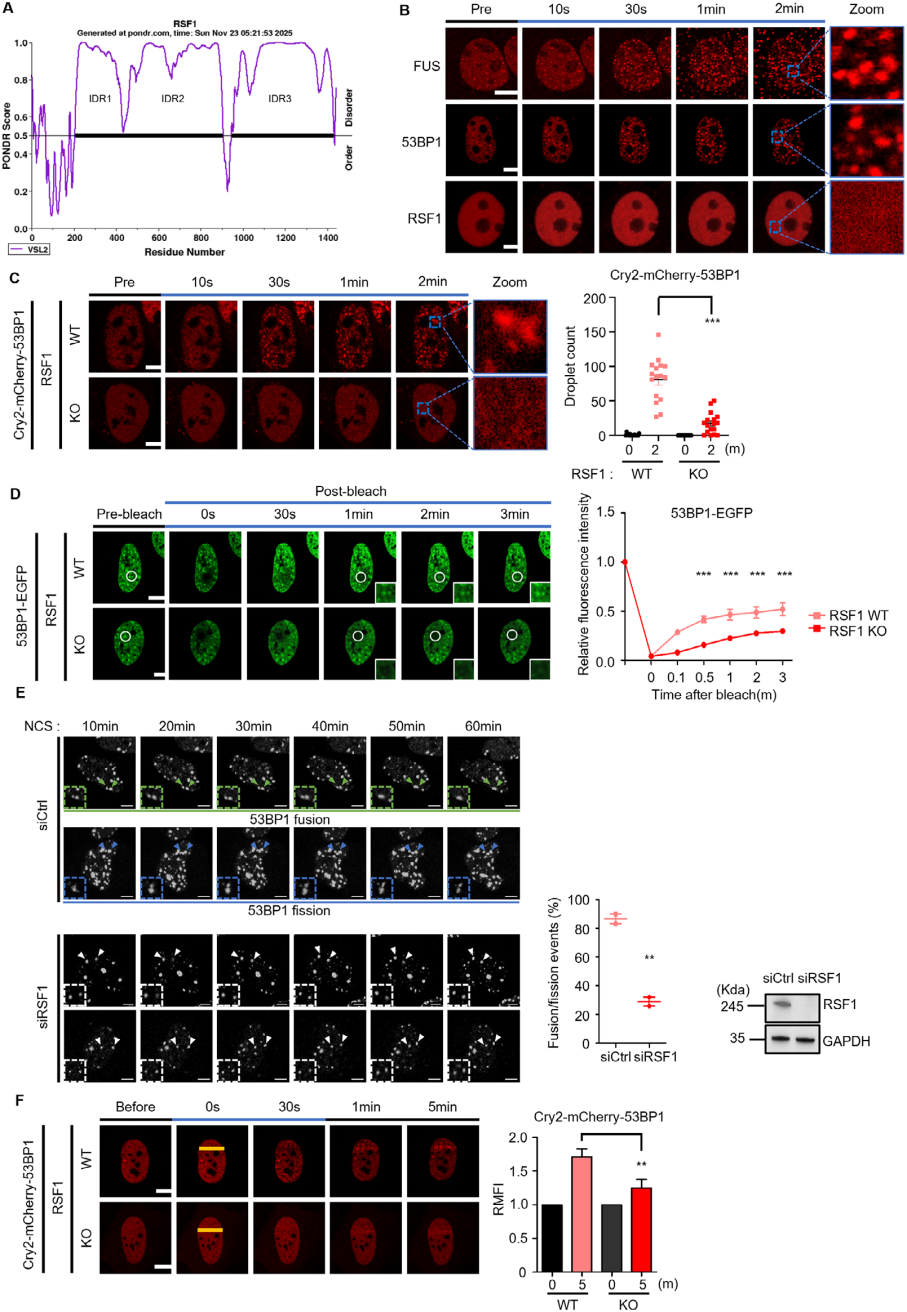
RSF1 facilitates the formation of 53BP1 liquid condensate but not FUS droplet. (A) Disordered region analysis of RSF1 using PONDR (http://www.pondr.com). (B) Light‐induced optoDroplet formation of Cry2‐mCherry‐FUS, Cry2‐mCherry‐53BP1 W1495A, and RSF1‐mCherry‐Cry2. Exogenous Cry2‐mCherry formed puncta upon stimulation of blue light (488 nm) in U2OS cells. (C) Representative images of Cry2‐mCherry‐53BP1 W1495A optoDroplet formation in RSF1 WT and RSF1 KO U2OS cells. Quantification of the number of 53BP1 optoDroplets per cell from five independent biological experiments (*n* = 5) is presented as mean ± SEM (right panel). Statistical significance was determined using an unpaired two‐tailed Student's t‐test. ***p < 0.001. Scale bar, 10 µm. (D) Fluorescence recovery after photobleaching (FRAP) of NCS (Neocarzinostatin)‐induced 53BP1‐EGFP puncta in RSF1 WT and KO U2OS cells. Quantification of fluorescence recovery kinetics in the bleached region, normalized to pre‐bleach levels, from three independent biological experiments (*n* = 3) is shown as mean ± SEM (right panel). Statistical significance was determined using an unpaired two‐tailed Student's t‐test. ***p < 0.001. Scale bar, 10 µm. (E) YFP‐53BP1 KI cells were treated with 200 ng mL^−1^ NCS to induce DNA breaks and imaged at 10 min intervals. Examples of YFP‐53BP1 fusions (green arrowheads and magnified regions) and fissions (blue arrowheads and magnified regions) are shown. Fusion and fission events were quantified from two independent biological experiments (*n* = 2) and are presented as mean ± SEM (right panels). A total of *n* = 60 siCtrl cells and *n* = 56 siRSF1 cells from two independent experiments were analyzed. Cells showing at least one fusion event accounted for 32/60 cells (53.3%) in siCtrl and 9/56 cells (16%) in siRSF1, whereas cells showing at least one fission event accounted for 20/60 cells (33.3%) and 7/56 cells (12.5%), respectively. RSF1 depletion efficiency was validated by immunoblotting. P‐values were calculated using Student's t‐test. P‐values were as follows: **p = 0.0062. Scale bar, 10 µm. (F) Light‐induced condensate formation and coupling to the DNA damage response were examined by combining optoDroplet activation (488 nm) with targeted microirradiation (405 nm) in RSF1 WT and KO U2OS cells. Quantification of fluorescence intensity at laser strips, normalized to background levels, from three independent biological experiments (*n* = 3) is presented as mean ± SEM (right panel). Statistical significance was determined using an unpaired two‐tailed Student's t‐test. P = 0.006. Scale bar, 10 µm.

Consistent with previous findings [26], both Cry2‐mCherry‐53BP1 W1495A and a prion‐like domain of FUS fused to mCherry‐Cry2 resulted in rapid light‐induced optoDroplet formation within 10–30 s. The W1495A mutation, located in the tandem Tudor domain of 53BP1, has been used to reduce chromatin‐ or protein‐interaction‐dependent effect and thereby assess the intrinsic phase‐separation propensity of 53BP1. In contrast, RSF1 WT‐mCherry‐Cry2 did not form the optoDroplet upon light stimulation (Figure 1B). Notably, even the RSF1 C2‐mCherry‐Cry2, which contains a large IDR, failed to form optoDroplet upon light stimulation, regardless of DNA damage (Figure S1B,C).

To examine the function of IDR in RSF1 rather than the formation of condensate by itself, next we examined whether RSF1 IDR contributes to other biomolecular condensates such as FUS or 53BP1‐mediated condensate. Upon light stimulation, Cry2‐mCherry‐53BP1 droplets readily formed but were disrupted by 1,6‐hexanediol treatment (Figure S1D), consistent with their liquid‐like properties [26]. Interestingly, we observed that Cry2‐53BP1 optoDroplet was markedly impaired in RSF1 KO cells compared to those in RSF1 WT cells (Figure 1C), suggesting that RSF1, and potentially its IDR, facilitates the assembly or stabilization of 53BP1‐mediated condensates. To exclude the possibility that this effect was specific to the W1495A mutant, we further examined Cry2‐53BP1 WT and found that its optoDroplet formation was also impaired in RSF1 KO cells (Figure S1E).

Then, we determined whether RSF1 affects the Cry2‐FUS optoDroplet formation. In contrast to the role of RSF1 on 53BP1 optoDroplet, formation of FUS optoDroplet was not affected by the presence of RSF1 (Figure S1F). Thus, the effect of RSF1 on LLPS formation was specific to the formation of 53BP1 condensate. Then we verified the effect of RSF1 on 53BP1‐induced phase separation by FRAP (fluorescence recovery after photobleaching) that determines the mobility of the phase‐separated liquid droplets. When the recovery of 53BP1‐EGFP was compared after photobleaching, we noticed much slower recoveries of 53BP1‐EGFP in RSF1 KO cells compared to those in RSF1 WT cells (Figure 1D). We further assessed the dynamics of endogenous 53BP1 condensates by live‐cell imaging of fusion and fission events using YFP‐53BP1 KI cell line after DNA damage. Immunoblot analysis confirmed that the YFP‐53BP1 KI protein was expressed at a level comparable to endogenous 53BP1 in parental cells (Figure S1G). RSF1 depletion strongly suppressed these fusion and fission events, supporting the idea that RSF1 promotes the dynamic liquid‐like properties of 53BP1 condensates in response to DNA damage (Figure 1E). Consistent with the impaired fusion and fission dynamics, RSF1 depletion delayed the accumulation of 53BP1 foci at early time points after DNA damage, whereas the number of foci became comparable at later time points (Figure S1H). In addition, 53BP1 foci area was largely reduced in RSF1 KO cells (Figure S1I).

Furthermore, we combined optoDroplet formation with laser microirradiation to assess whether RSF1 influences the spatial formation of 53BP1 condensates at sites of DNA damage. Cry2‐53BP1 was expressed in RSF1 WT and KO cells, followed by global exposure with 488 nm laser and localized microirradiation with 405 nm laser to induce DNA damage stripes. Notably, we found that 53BP1 droplets are enhanced at microirradiated sites in WT, whereas this localized droplet enrichment was largely absent in KO cells (Figure 1F). Thus, we concluded that RSF1 plays a significant role in the formation of 53BP1‐mediated liquid condensates in the DNA damage compartment.

### 2.2 IDR3 Of RSF1 Is Sufficient for the Formation of 53BP1‐Mediated LLPS

We examined which domain of RSF1 is involved in the regulation of the 53BP1 liquid condensate. N‐terminal domain of RSF1 comprises an alpha helical module that interacts with the SLIDE domain of SNF2H and nucleosomal linker DNA [38, 54, 55], followed by glutamate‐rich region. In the middle, a plant homeodomain‐type zinc domain (PHD) (aa 893–939) is inferred to the involvement of transcriptional regulation and protein‐protein interaction [55] but its functional role in RSF1 has not been addressed. Three nucleus localization signal sites (NLS) exist between aa 1084–1244 of RSF1.

We examined the effects of two N‐terminal containing deletion mutants (RSF1 N2, N3) and two C‐terminal mutants (RSF1 C1, C2) on 53BP1 droplet formation (Figure 2A) [49]. RSF1 N2 and N3 were predominantly localized in the cytoplasm and failed to rescue 53BP1 droplet formation. In contrast, C1 failed to rescue 53BP1 droplet formation, whereas C2 rescued the droplet formation (Figure 2B). Because RSF1 N2 and N3 lack a nuclear localization signal, we added NLS to these constructs to clearly examine the domain of RSF1 and examined their ability to induce 53BP1 droplet formation. When RSF1 WT and these mutants were co‐expressed with Cry2‐53BP1 in RSF1 KO cells, RSF1 WT restored the 53BP1 liquid optoDroplets whereas RSF1 N2, N3 were unable to form 53BP1 droplets (Figure S2A). RSF1 N3 showed a slight increase in 53BP1 droplet formation, suggesting that the N‐terminal fragments were insufficient to induce 53BP1 droplet formation. Of note, RSF1 C2 containing Arg‐rich region was capable of stimulating 53BP1 liquid droplet as RSF1 WT. In contrast, RSF1 C1 did not recover the 53BP1 liquid optoDroplet formation.

**FIGURE 2:**
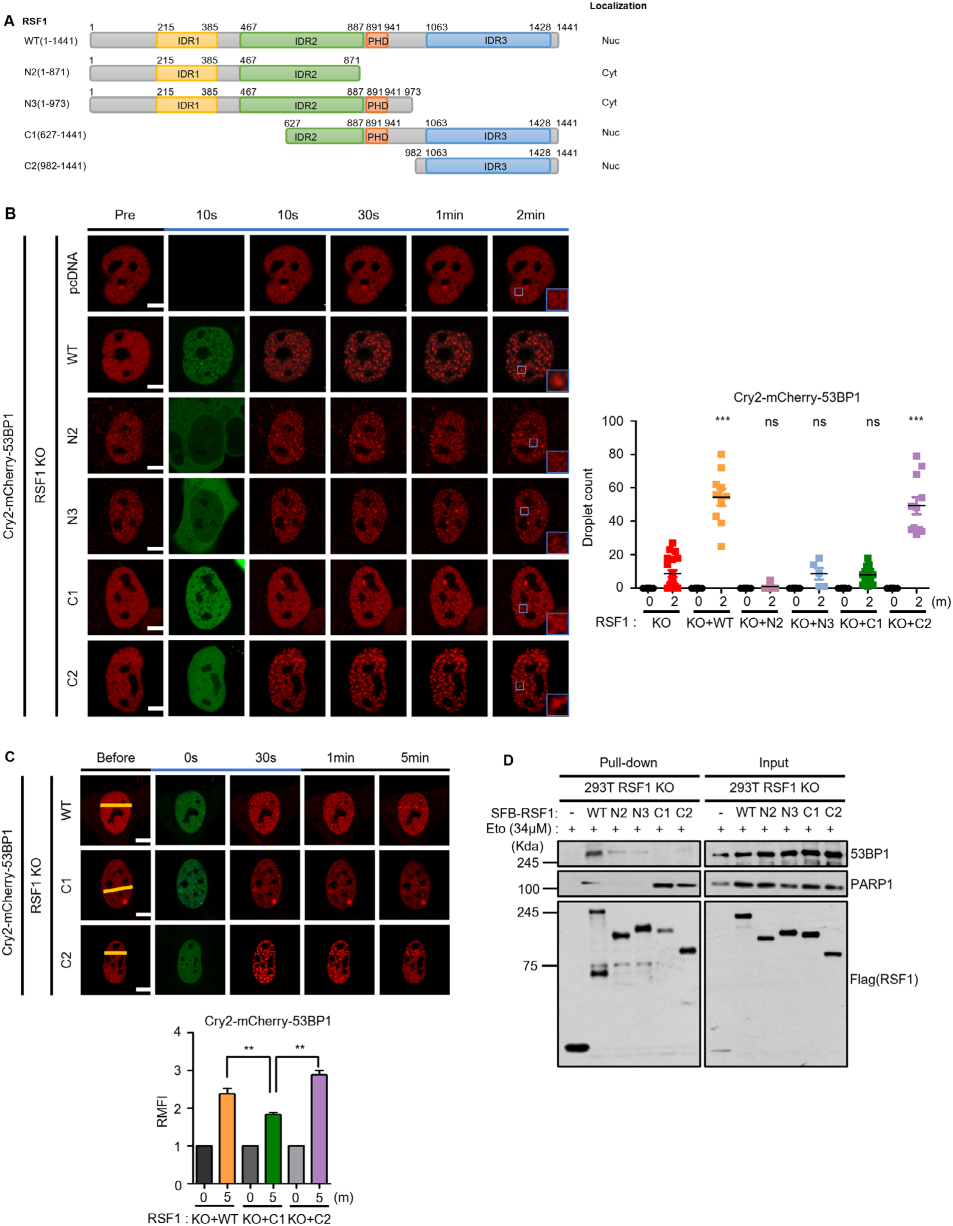
IDR3 of RSF1 is sufficient for the formation of 53BP1‐mediated LLPS. (A) Schematic illustration of domain structure of WT and RSF1 mutants. (B) Light‐induced optoDroplet formation of Cry2‐mCherry‐53BP1 W1495A in RSF1 KO U2OS cells expressing RSF1‐GFP truncation mutants. Representative images before and 2 min after 488 nm light activation are shown. Droplet numbers were quantified based on red puncta detected at 2 min after light activation (right panel). Quantification of 53BP1 optoDroplet numbers per cell from three independent biological experiments (*n* = 3) is presented as mean ± SEM (right panel). P‐values were calculated using one‐way ANOVA followed by Tukey's multiple‐comparison test. P‐values were as follows: ***P≤0.001, ns = >0.999, 0.108. Scale bar, 10 µm. (C) U2OS RSF1 KO cells expressing RSF1 WT‐GFP or mutant‐GFP constructs were transfected with Cry2‐mCherry‐53BP1, first exposed to 488 nm light to induce droplet formation, and subsequently subjected to 405 nm laser micro‐irradiation to generate localized DNA damage. Quantification was performed using the red fluorescence signal at 5 min after micro‐irradiation (lower panel). Fluorescence intensity at laser strips was normalized to background fluorescence intensity, from three independent biological experiments (*n* = 3) is presented as mean ± SEM. P‐values were calculated one‐way ANOVA followed by Tukey's multiple‐comparison test. P‐values were as follows: **p = 0.010, 0.001. Scale bar, 10 µm. (D) 293T RSF1 KO cells were transfected with vector or SFB‐RSF1 mutants. Cells were treated with etoposide (34 µM) for 6 h to induce DSBs. Cells were lysed, subjected to streptavidin beads pull‐down assay and analyzed by immunoblotting with the indicated antibodies.

RSF1 C1 consists of partial IDR2, PHD and IDR3 while RSF1 C2 is mainly composed of IDR3 (Figure 2A). To verify these differences between RSF1 WT and C1 at the damaged compartment, we carried out microirradiation after stimulation of optoDroplet formation at 488 nm. We observed a robust accumulation of 53BP1 droplets at the DNA strip at 5 min in the RSF1 WT‐reconstituted cell whereas no change was observed in the RSF1 C1‐expressing cell (Figure 2C). Notably, RSF1 C2 showed even faster recruitment of 53BP1 at DSB sites. We further examined fusion and fission events of endogenous 53BP1 condensates using YFP‐53BP1 KI cells. Consistent with the optoDroplet formation assay, 53BP1 fusion and fission dynamics were largely abolished in RSF1 C1 expressing cells, whereas these dynamic events were preserved in cells expressing RSF1 WT or C2 (Figure S2B).

Thus, RSF1 WT and C2 showed similar behavior in the formation of 53BP1‐mediated liquid condensates whereas C1 showed a distinct behavior at damaged chromatin. To illustrate whether these mutants showed differences in 53BP1 binding, we carried out a pull‐down assay. RSF1 WT strongly interacted with 53BP1 but RSF1 C1 and C2 were not able to do (Figure 2D). Because RSF1 C2 promoted the formation of 53BP1 optoDroplet (Figure 2B), the ability to interact with 53BP1 does not account for the formation of LLPS.

Interestingly, we found that RSF1 WT, C1, and C2 mutants, but not N2 or N3, interact with PARP1, with the C1 mutant exhibiting stronger binding affinity than the WT or C2 forms. This suggests that RSF1 may regulate condensate formation by modulating PARP1 rather than through direct interaction with 53BP1. Together, the data here showed that IDR3 of RSF1 is sufficient for the formation of 53BP1‐mediated LLPS, through modulating PARP1.

### 2.3 RSF1 Regulates PAR Turnover to Promote the Transition to 53BP1 Condensates

To explore whether the enhanced interaction between RSF1 C1 and PARP1 contributes to its post‐translational modification and the suppression of 53BP1 condensate formation, we next examined whether RSF1 is directly PARylated by PARP1. In an in vitro PARylation assay, PARP1 catalyzed the PARylation of RSF1. Notably, both RSF1 WT and C1 showed detectable PARylation, whereas PARylation of RSF1 C2 was not observed (Figure S3A). Consistent results were observed when RSF1 WT and mutant constructs were expressed in cells and followed by immunoblots with anti‐PAR antibody (Figure S3B). This suggests that RSF1 PARylation itself is not required for 53BP1 droplet formation; rather, the temporal control of RSF1 PARylation and subsequent dePARylation particularly across IDR2 and IDR3 is critical for proper 53BP1 condensate assembly. We next compared PAR accumulation after DNA damage induced by H_2_O_2_. In RSF1 KO cells, PAR accumulation was enhanced upon H_2_O_2_ treatment (Figure 3A). In RSF1 WT‐expressing cells, PAR accumulation peaked at 10 min and resolved by 30 min. On the other hand, in RSF1 knockout cells, PAR chains persisted until 30 min, and reconstitution with RSF1 WT and C2 restored timely PAR degradation. Moreover, a stronger accumulation of PAR was observed in cells expressing RSF1 C1 and persisted until 30 min (Figure 3B), highlighting that C1 impairs proper PAR turnover at damaged chromatin.

**FIGURE 3:**
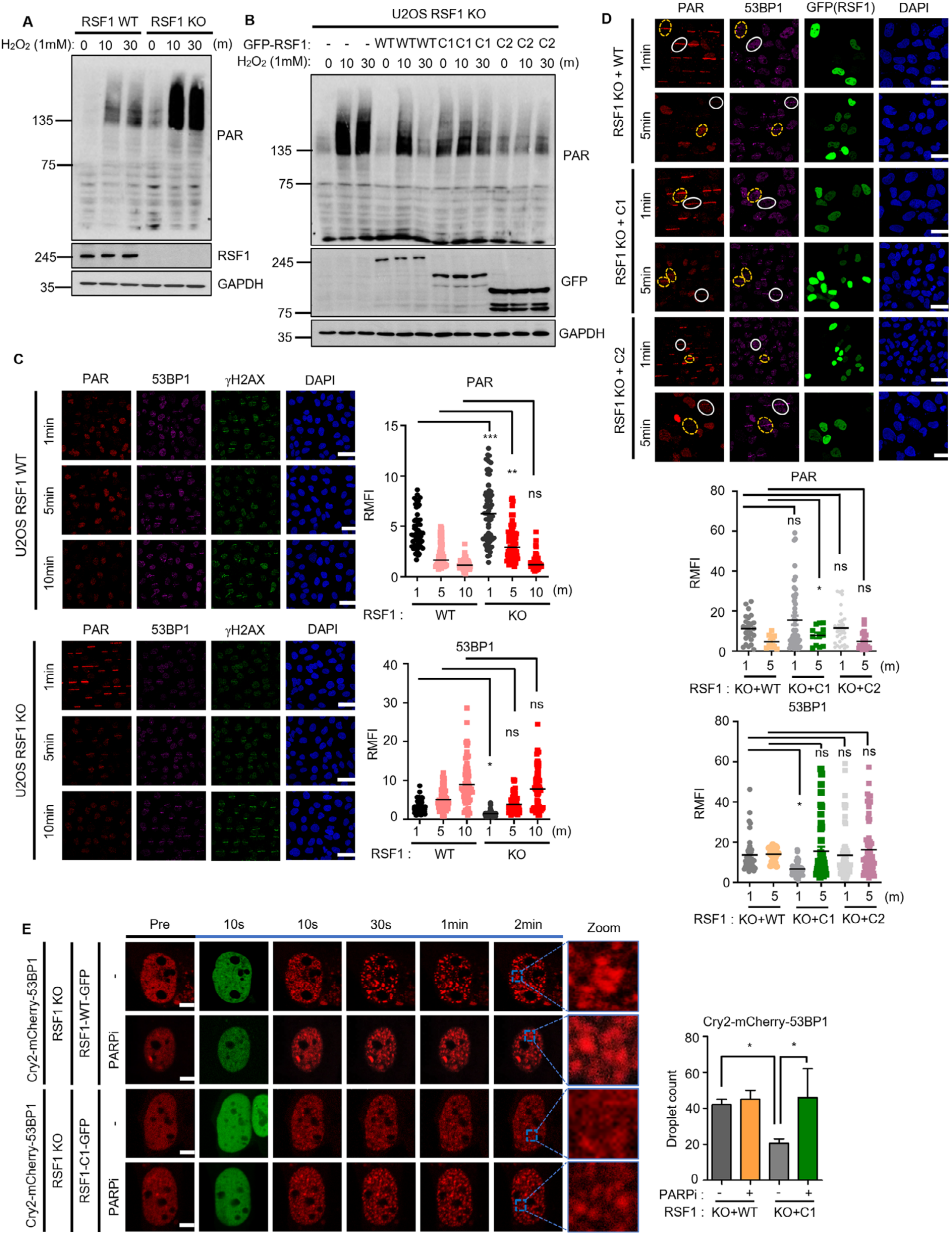
RSF1 regulates PAR turnover to promote the transition to 53BP1 condensates. (A) Whole‐cell lysates from U2OS RSF1 WT and KO cells were treated with H_2_O_2_ (1 mM) for the indicated time points and analyzed by immunoblotting with the indicated antibodies. (B) Whole‐cell lysates from U2OS RSF1 KO cells reconstituted with GFP‐RSF1 WT, C1, or C2 were treated with H_2_O_2_ (1 mM) for the indicated time points and analyzed by immunoblotting with the indicated antibodies. (C) U2OS RSF1 WT and KO cells were subjected to laser microirradiation and fixed at the indicated time points. PAR persistence at DNA damage sites was visualized by co‐staining with PAR, 53BP1and γH2AX. Quantification of PAR and 53BP1 fluorescence intensity at laser tracks, normalized to background levels, from three independent biological experiments (*n* = 3) is shown as mean ± SEM (right panel). P‐values were calculated using one‐way ANOVA followed by Tukey's multiple‐comparison test. P‐values were as follows: PAR, ***p≤0.001, **p = 0.002, ns = 0.928. 53BP1, *p = 0.041, ns = 0.270,0.287. Scale bar, 10 µm. (D) U2OS RSF1 KO cells expressing GFP–RSF1 WT, C1, or C2 were subjected to laser microirradiation and fixed at the indicated time points. Cells expressing RSF1 WT or mutants are indicated (yellow, rescued cells; white, KO cells). Quantification of PAR and 53BP1 fluorescence intensity at laser tracks, normalized to background levels, from three independent biological experiments (*n* = 3) is shown as mean ± SEM (right panel). P‐values were calculated using one‐way ANOVA followed by Tukey's multiple‐comparison test. P‐values were as follows: PAR: *p = 0.038, ns = >0.999, 0.985, >0.999. 53BP1, *p = 0.034, ns = >.999, 0.995,0.969. Scale bar, 10 µm. (E) U2OS RSF1 KO cells reconstituted with RSF1 WT or C1 and expressing Cry2‐mCherry‐53BP1 W1495A were illuminated with 488 nm light to induce 53BP1 optoDroplet formation. Cells were treated with or without a PARP inhibitor (PARPi) and imaged at the indicated time points. Quantification of 53BP1 droplets per nucleus from three independent biological experiments (*n* = 3) is presented as mean ± SEM (right panel). P‐values were calculated using an unpaired two‐tailed Student's t‐test. P‐values were as follows: *p = 0.0108, 0.02. Scale bar, 10 µm.

To directly assess PAR turnover at damaged chromatin, we next visualized PAR and 53BP1 dynamics at DSB sites. In RSF1 WT‐reconstituted cells, PAR chains appeared rapidly at early time points and disappeared within 5 min. However, in C1‐reconstituted cells, PAR chains persisted at DSB sites even at 5 min and delayed 53BP1 recruitment (Figure 3D and Figure S3C), suggesting a failure to resolve PARP1‐mediated condensates and a disruption in the transition to 53BP1 condensate formation. Similar to RSF1 WT, RSF1 C2 promoted rapid PAR clearance within 5 min and proper recruitment of 53BP1 to damaged chromatin.

Finally, to determine whether excessive PARylation affects 53BP1 condensate formation, we treated C1‐reconstituted cells with a PARP inhibitor (Figure 3E). Remarkably, PARP inhibition restored 53BP1 condensate formation, highlighting the importance of balanced PARylation‐dePARylation dynamics at damaged chromatin for proper assembly of 53BP1 biomolecular condensates.

### 2.4 RSF1 Regulates PARG but Not PARP1 Recruitment to DNA Damage Sites, Affecting 53BP1 Condensate Formation

Because PARylation on the damaged chromatin is regulated by PARP1 and PARG [56, 57], we first analyzed whether RSF1 expression affects PARP1 recruitment following microirradiation. PARP1 rapidly accumulated at DSB sites regardless of RSF1, indicating that PARP1 recruitment is independent of RSF1 expression (Figure S4A,B). In contrast, RSF1 localization at DSBs required PARP1 (Figure S4C,D). Depletion of PARP1 or treatment with a PARP inhibitor markedly reduced RSF1 recruitment.

We next investigated whether RSF1‐mediated 53BP1 condensate formation involves PARG, the principal PAR‐degrading enzyme. RSF1 KO cells exhibited delayed PARG recruitment, with detectable signals only appearing at 10 min after microirradiation (Figure 4A). We next compared RSF1 WT and C1 in the recruitment of PARG following microirradiation. PARG rapidly accumulated at DNA damage regions within 5 min in RSF1 WT cells. In contrast, cells expressing RSF1 C1 exhibited delayed PARG recruitment, with detectable PARG signals only appearing at 10 min post‐irradiation, resembling the phenotype observed in RSF1 KO cells (Figure 4B). These results indicate that delayed PARG recruitment disrupts PAR turnover in RSF1 KO and C1 expressing cells, thereby impairing 53BP1 condensate formation. To mimic PAR dynamics in RSF1 knockout cells, we depleted PARG and analyzed 53BP1 optoDroplet formation. PARG knockdown markedly suppressed 53BP1 optoDroplet formation in RSF1‐reconstituted cells, suggesting that persistent PAR accumulation interferes with 53BP1 condensate assembly (Figure 4C). Likewise, FUS overexpression, which stabilizes PAR‐FUS condensates, inhibited 53BP1 droplets (Figure S4E). RSF1 itself did not alter FUS droplet formation (Figure S1F), but persistent PAR accumulation blocked RSF1‐driven 53BP1 assembly. Thus, RSF1 ensures proper PAR turnover at damaged chromatin, a prerequisite for productive 53BP1 condensate formation.

**FIGURE 4:**
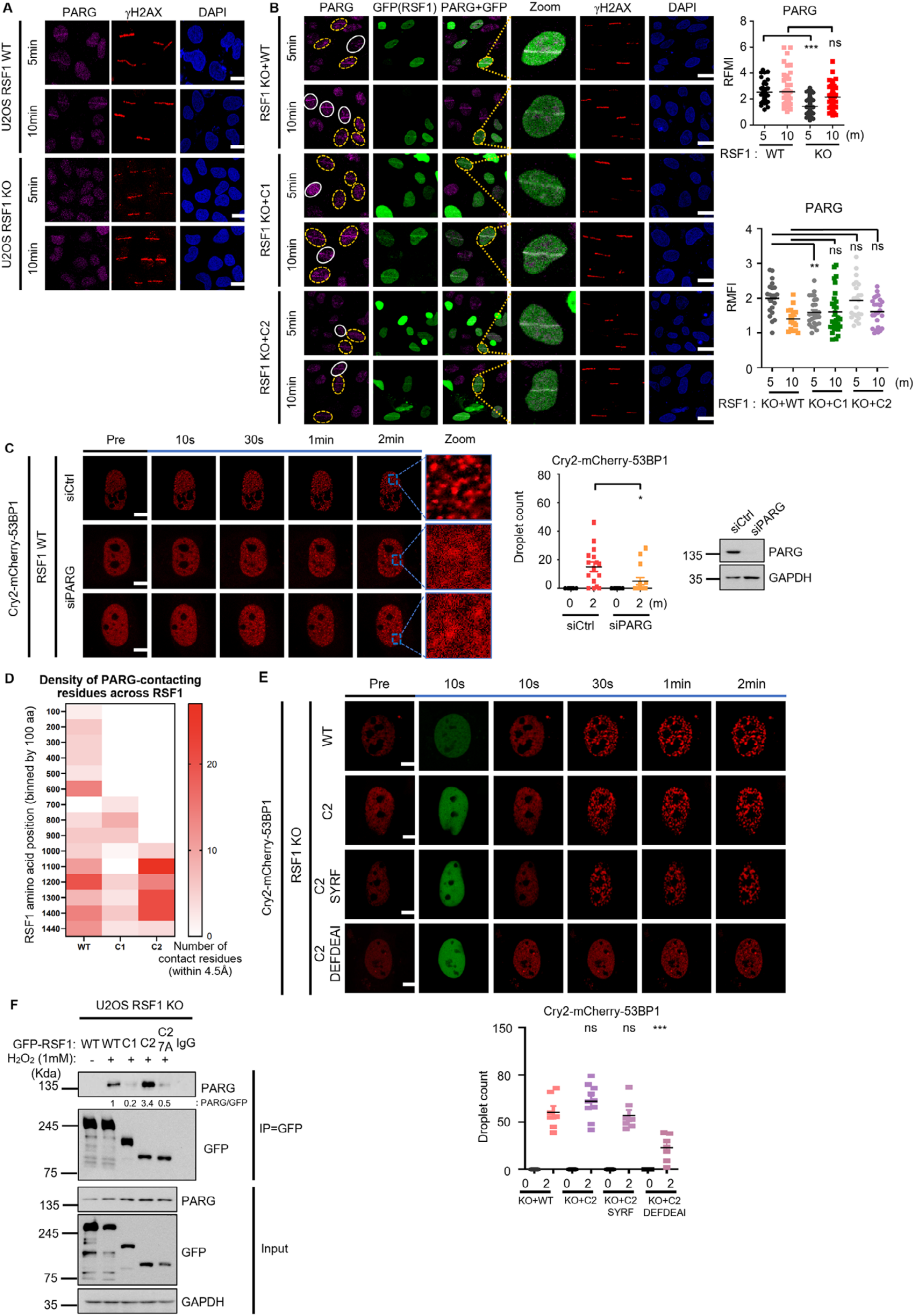
RSF1 regulates PARG but not PARP1 recruitment to DNA damage sites, affecting 53BP1 condensate formation. (A) Immunofluorescence analysis of PARG and γH2AX in U2OS RSF1 WT and KO cells after laser microirradiation. Cells were fixed at 5 and 10 min to examine PARG recruitment at damaged chromatin. Quantification of PARG fluorescence intensity at laser strips, normalized to background fluorescence intensity, from three independent biological experiments (*n* = 3) is presented as mean ± SEM (lower panel). P‐values were calculated using an unpaired two‐tailed Student's t‐test. P‐values were as follows: ***p≤0.0001, ns = 0.108. Scale bar, 10 µm. (B) Immunofluorescence analysis of PARG and GFP–RSF1 WT or GFP–RSF1 C1 in RSF1 KO cells following microirradiation. Cells expressing RSF1 WT or C1 are indicated (yellow, rescued cells; white, KO cells). Quantification of PARG–GFP fluorescence intensity was measured at laser tracks and normalized to background fluorescence intensity, from three independent biological experiments (*n* = 3) is presented as mean ± SEM (lower panel). P‐values were calculated using an unpaired two‐tailed Student's t‐test. P‐values were as follows: **p = 0.0024, ns = 0.445. Scale bar, 10 µm. (C) OptoDroplet assay of Cry2‐mCherry‐53BP1 W1495A in U2OS RSF1 WT cells transfected with siCtrl or siPARG. PARG depletion efficiency was validated by immunoblotting. Cells were illuminated with 488 nm light to induce 53BP1 droplet formation, and the number of droplets per nucleus was quantified after 2 min from three independent biological experiments (*n* = 3) is presented as mean ± SEM. P‐values were calculated using an unpaired two‐tailed Student's t‐test. P‐values were as follows: *p = 0.021. Scale bar, 10 µm. (D) Heatmap analysis representing the density of interaction interfaces. The spatial distribution of interacting residues situated within a 4.5Å threshold distance from PARG was quantified. The color intensity (white to red) corresponds to the contact residue density. (E) Light‐induced optoDroplet formation of Cry2‐mCherry‐53BP1 W1495A in RSF1 KO U2OS cells expressing RSF1 truncation mutants. Representative images before and 2 min after 488 nm light activation are shown. Droplet numbers were quantified based on red puncta detected at 2 min after light activation. Quantification of 53BP1 optoDroplet numbers in individual cells from three independent experiments (*n* = 3) is presented as mean ± SEM. P‐values were calculated using one‐way ANOVA with Tukey's post‐test. P‐values were as follows: ***p≤0.001, ns = 0.203, 0.998. Scale bar, 10 µm. (F) U2OS RSF1 KO cells were transfected with GFP‐RSF1 WT, C1, C2, or C2 7A mutant. Cells were treated with H_2_O_2_ (1 mM) for 10 min to induce DNA damage and subjected to immunoprecipitation using an anti‐GFP antibody, followed by immunoblotting with the indicated antibodies.

To gain structural insight into how RSF1 engages PARG, we performed in silico structural modeling using AlphaFold‐Multimer to predict complex structures of PARG with full‐length RSF1, C1, and C2. The top‐ranked predicted structures were visualized and analyzed using Mol* Viewer (Figure S5A). To compare the predicted PARG‐binding interfaces among full‐length RSF1, C1, and C2, we extracted RSF1 residues located within 4.5 Å of PARG and quantified the density of PARG‐contacting residues per 100 amino acids across each RSF1 construct (Figure 4D). This analysis revealed that the contact‐residue distribution of C2 was more similar to that of full‐length RSF1 than to that of C1, consistent with the ability of C2, but not C1, to support 53BP1 condensate formation. Manual inspection of the predicted PARG–RSF1 interface identified three representative proximal interaction regions in full‐length RSF1: SYRFDEFDEAI, KKYSDDDEEEESEEN, and EDELLRVTDLVDYVCNSEQ (Figure S5B). Among these, the EDELLRVTDLVDYVCNSEQ region was not positioned at the PARG‐contacting interface in either C1 or C2. In contrast, the SYRFDEFDEAI‐ and KKYSDDDEEEESEEN‐containing regions in C2 were predicted to contact PARG in a manner similar to full‐length RSF1. Notably, the SYRFDEFDEA‐containing region was well conserved and predicted to directly contact PARG in both full‐length RSF1 and C2, whereas this contact was not observed in the C1 complex model (Figure S5C). These results suggest that, although C1 contains the entire C2/IDR3 sequence, its structural context may alter the spatial presentation of this region, thereby preventing productive PARG engagement.

Based on these structural predictions, we hypothesized that the SYRFDEFDEAI‐containing region contributes to the interaction between C2 and PARG. To further evaluate this possibility, we performed docking simulations using HADDOCK2.4 to compare the predicted binding of PARG to WT C2 and C2 mutants in which residues within this region were progressively substituted with alanine. WT C2 showed the most favorable predicted docking profile, whereas alanine substitutions within the SYRFDEFDEAI sequence progressively reduced the predicted interaction with PARG (Table S1). In particular, complete alanine substitution of this region markedly reduced the predicted buried surface area and docking score, supporting the possibility that this sequence contributes to structural stabilization of the C2–PARG complex.

To validate these predictions experimentally, we generated C2 substitution mutants targeting either the SYRF sequence or the DEFDEAI sequence and examined their ability to promote 53BP1 droplet formation. Mutation of the DEFDEAI motif to AAAAAAA (hereafter referred to as C2 7A) strongly impaired 53BP1 droplet formation, whereas mutation of the SYRF motif did not impair 53BP1 droplet formation (Figure 4E). We further assessed the interaction of these mutants with PARG by co‐immunoprecipitation and found that both C1 and the C2 7A mutants showed reduced PARG association (Figure 4F). Furthermore, analysis of PAR and 53BP1 dynamics in RSF1 C2‐7A‐expressing cells showed defective PAR turnover and impaired 53BP1 recruitment (Figure S5D). These results indicate that the DEFDEAI‐containing region within C2 is important for PARG interaction and that this interaction is required for RSF1‐mediated PAR turnover and subsequent 53BP1 condensate formation at DNA damage sites.

### 2.5 RSF1‐Mediated Stimulation of 53BP1 Liquid Droplets Directly Links to Its Transcriptional Regulation of p53 Target Genes

We previously reported that RSF1 stimulates the transcription of p53 target genes upon DNA double strand breaks and RSF1 KO reduces the binding of p53 to target gene promoters in CDKN1A (p21CIP1), BAX and PUMA [47]. It is also shown that disruption of 53BP1 phase separation diminishes p53 target gene expression [26].

As RSF1 plays a key role in the sequential formation of LLPS in the DNA damage compartment, we investigated whether this has an impact on p53‐related gene transcription. In line with our previous findings, the administration of etoposide resulted in elevated levels of p53, p21CIP1, and γH2AX. The inclusion of 1,6‐Hexanediol (HD), which dissolves LLPS, led to a marked decrease in these levels (Figure 5A). Similarly, increased p21CIP1 and PUMA mRNA levels were suppressed by co‐treatment with HD (Figure 5B). We also found that p53 protein was enriched in 53BP1 optoDroplets in RSF1 WT cells as previously shown [26], whereas they were hardly co‐localized in RSF1 KO cells (Figure 5C), suggesting that RSF1‐mediated liquid condensates may be important for their co‐localization as well as for p53 target gene expression.

**FIGURE 5:**
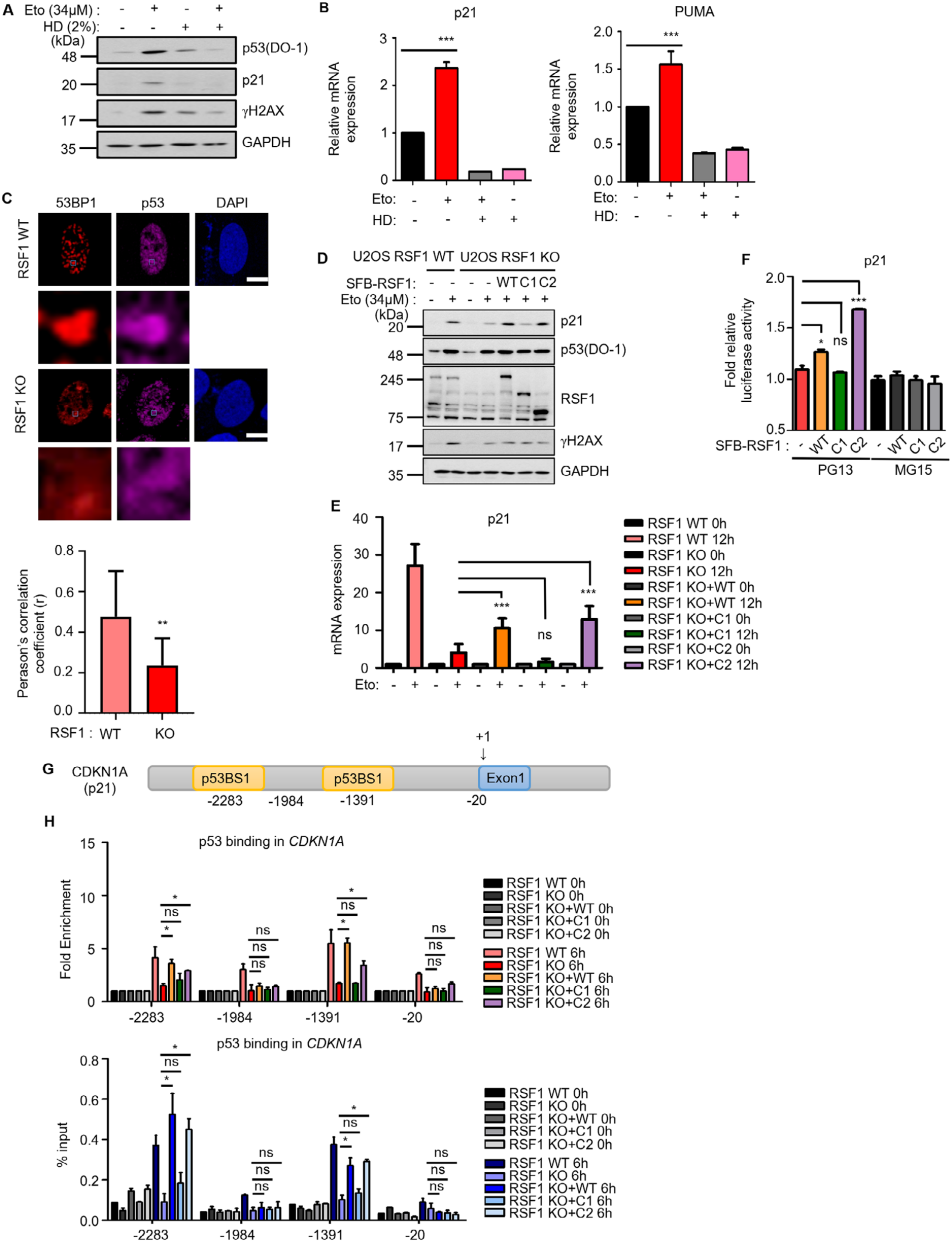
RSF1‐mediated stimulation of 53BP1 liquid droplets directly links to its transcriptional regulation of p53 target genes. (A) U2OS cells were treated with Etoposide (34 µM) for 6 h in the absence or presence of 2% 1,6‐hexanediol. Whole‐cell extracts were analyzed by immunoblotting with the indicated antibodies. (B) qPCR analysis of p21 and PUMA induction following etoposide treatment (34 µM, 6 h) with or without 2% 1,6‐hexanediol. Data are presented as mean ± SEM from three independent biological experiments (*n* = 3). Statistical significance was determined using an unpaired two‐tailed Student's t‐test. P‐values were as follows: ***p≤ 0.001. (C) Light‐induced optoDroplet formation of Cry2‐mCherry‐53BP1 W1495A in U2OS RSF1 WT and KO cells, fixed 2 min after 488‐nm illumination. Endogenous p53 was co‐stained. Pearson's correlation coefficients for 53BP1 and p53 colocalization from three independent biological experiments (*n* = 3) are presented as mean ± SEM. Statistical significance was determined using an unpaired two‐tailed Student's t‐test. P‐values were as follows: **p≤0.01. Scale bar, 10 µm. (D) U2OS RSF1 KO cells were transfected with SFB empty vector or SFB‐tagged RSF1 WT,C1, or C2 and treated with etoposide (34 µM) for 6 h. Whole‐cell extracts were analyzed by immunoblotting with the indicated antibodies. (E) Fold induction of the p53 target gene p21 in U2OS RSF1 WT and KO cells transfected with pcDNA or V5‐tagged RSF1 truncation mutants following etoposide treatment (34 µM). qPCR data were normalized to basal expression in untreated cells. Data are presented as mean ± SEM from three independent biological experiments (*n* = 3). Statistical significance was determined using one‐way ANOVA followed by Tukey's multiple‐comparison test. P‐values were as follows: ***p≤0.001, ns = 0.569. (F) Luciferase assay measuring p53 transcriptional activity in U2OS RSF1 KO cells transfected with the indicated plasmids. PG13 contains 13 tandem p53 response elements, whereas MG15 contains 15 mutated p53 response elements. Data represent mean ± SEM from three independent experiments. P‐values were calculated using one‐way ANOVA followed by Dunnett's multiple‐comparison test. P‐values were as follows: ***p≤0.001, *p = 0.034. ns = 0.998. (G) Schematic diagram of the CDKN1A (p21) promoter. (H) ChIP analysis of p53 binding at the CDKN1A promoter in U2OS RSF1 WT and KO cells transfected with pcDNA or V5‐tagged RSF1 truncation mutants after etoposide treatment (34 µM). p53 enrichment was calculated as % input (lower panel) and normalized to untreated controls (upper panel). Data are presented as mean ± SEM from three independent biological experiments (*n* = 3). Statistical significance was determined using one‐way ANOVA followed by Tukey's multiple‐comparison test. *p*‐values were as follows: *p≤0.05, ns = >0.999.

Because RSF1 WT C1, and C2 mutant displayed distinct phenotypes in the formation of 53BP1 phase separation, we examined whether these two constructs showed the differences in p53 target gene expression. A moderate increase in p21CIP1 mRNA and protein expression was observed in RSF1 KO cells reconstituted with RSF1 WT and C2 whereas it was not observed in RSF1 C1 expressing cells (Figure 5D,E). We also tested luciferase activity driven by p53 binding sites (PG13), which was significantly increased in RSF1 WT and C2 restored U2OS RSF1 KO cells after etoposide treatment. The negative control with the binding site mutants (MG15) did not show any significance in luciferase activity (Figure 5F).

Next, the p53 binding ability to p53 response elements (p53REs) located upstream of the p21CIP1 gene was examined by chromatin immunoprecipitation (ChIP) assay. A schematic map of the CDKN1A promoter illustrates the two p53REs (‐2283 and ‐1391) and the two non‐p53RE regions (‐1984 and ‐20) (Figure 5G). The gene was assessed after DNA‐damaging drug treatment in RSF1 WT‐, C1‐, and C2‐reconstituted RSF1 KO U2OS cells. p53 binding at the p53REs in the CDKN1A gene was specific and was significantly enriched after 6 h of etoposide treatment in RSF1 WT‐ and C2‐expressing cells, whereas p53 binding was markedly reduced in RSF1 C1‐reconstituted cells (Figure 5H).

We next asked whether the ability of RSF1 fragments to regulate 53BP1 condensate formation is associated with functional DNA damage responses. In clonogenic survival assays, RSF1 KO cells showed increased sensitivity to etoposide and phleomycin compared with RSF1 WT cells. Reconstitution with full‐length RSF1 restored cell survival, whereas RSF1 C1 failed to provide efficient protection. RSF1 C2 partially improved survival compared with C1‐expressing cells, although it did not fully restore resistance to the level observed with full‐length RSF1 (Figure S6A). These results suggest that the C2 region contributes to DNA damage tolerance, but that additional regions of RSF1 are likely required for full repair capacity. We further examined DNA repair pathway activity using DR‐GFP and EJ5‐GFP reporter assays. Depletion of RSF1 impaired both HR‐ and end‐joining‐associated reporter activity, and full‐length RSF1 reconstitution partially restored these activities (Figure S6B). In contrast, C1 and C2 fragments did not fully rescue reporter activity, suggesting that restoration of 53BP1 condensate formation by C2 is not sufficient to reconstitute all DNA repair functions of full‐length RSF1.

Together, these data suggest that the RSF1‐PARG‐PAR turnover supports 53BP1 condensate formation and p53‐dependent transcriptional responses after DNA damage.

## 3 Discussion

The present study elucidated that RSF1 plays a regulatory role in the dynamic regulation of liquid condensates formed at DNA damage sites. RSF1 did not affect the initial formation of PAR‐FUS liquid condensates; however, its absence resulted in significant impairment of 53BP1 liquid condensate formation. It is noteworthy that RSF1 regulates the timely recruitment of PARG, which is responsible for the removal of PAR chains. This permits the sequential formation of 53BP1 liquid condensates and p53‐induced p21WIP1 transcription (Figure S7). In light of these findings, RSF1, which contains a substantial proportion of intrinsically disordered regions, plays a multifunctional role in the DNA damage response and the damage‐induced gene transcription, thereby contributing to the maintenance of genome integrity.

Proteins containing IDRs are frequently enriched within phase‐separated compartments [34, 35]. However, the functional contribution of IDRs in chromatin‐associated proteins to genome stability has remained poorly understood. Our structural prediction using AlphaFold and PONDR identified three IDRs within RSF1, suggesting that RSF1 may act as a regulatory scaffold within DNA damage‐induced condensates (Figure 1A and Figure S1A). Among these, IDR3, contained within the RSF1 C2, was sufficient to stimulate the formation of 53BP1 liquid condensates (Figure 2B), a process that is specific to 53BP1 condensates as RSF1 did not affect the PAR‐FUS condensates (Figure 1C and Figure S1F). Notably, RSF1 itself did not undergo phase separation, supporting the notion that multivalency within IDRs, rather than the intrinsic disordered three‐dimensional structure of protein itself, drives LLPS through transient and flexible interactions within PAR‐FUS condensates [53, 58, 59]. Interestingly, RSF1 C1, which also contains IDR3, failed to stimulate condensate formation, suggesting that the presence of IDR3 alone is not sufficient, and that the structural context in which IDR3 is presented is critical for its function. Our structural modeling and biochemical analyses suggest that C1 fails to engage PARG efficiently, whereas C2 retains PARG‐binding ability and supports 53BP1 condensate assembly. Furthermore, RSF1 WT interacted with both PARP1 and 53BP1 following DNA damage, while C1 and C2 mutants retained PARP1 binding but lost interaction with 53BP1. Given that RSF1 C2 still promoted 53BP1 condensate assembly, our data indicates that dynamic PAR turnover, rather than a direct RSF1‐53BP1 interaction, is critical for the proper formation of 53BP1 condensates. Furthermore, the arginine‐rich composition of RSF1 in C1 mutant may underlie its phase behavior, as frame mutations in arginine‐rich IDRs have the potential to disrupt protein function through aberrant phase behavior, leading to pathological diseases [60]. Thus, arginine composition likely governs distinct phase behaviors and functions of RSF1 within damaged chromatin compartments.

LLPS within the DNA damage compartment is a crucial biological phenomenon that enables the efficient sequestration of DDR factors into membraneless organelles [26, 33, 61]. The PAR‐FUS and 53BP1 condensates differ in that MDC1 is limited to the PAR condensate, whereas damage‐induced long non‐coding RNAs (dilncRNAs) are crucial for 53BP1 condensate formation [33]. Dissolution of PAR‐FUS condensates is achieved by the recruitment of PARG and FUS phosphorylation by DNA‐PK [62, 63]. On the other hand, the ability of 53BP1 to undergo LLPS depends on its oligomerization domain instead of its unstructured N‐terminal domain [26]. However, the full understanding of spatial and temporal regulation in the sequential formation, maintenance and disassembly of these condensates has yet to be achieved. In this study, we found impaired PAR balance and subsequent disruption of 53BP1 recruitment and condensate formation in RSF1 KO cells (Figure 2B and Figure 3C,D) Upon reconstitution of RSF1 WT, the PAR chains in these cells were found to be extinguished. It became evident that this phenomenon was attributable to the delayed recruitment of PARG into damaged DNA strips of RSF1 KO cells (Figure 4A). It is established that PARG recruitment to sites of damaged DNA is known to be dependent on PARP1 and PAR synthesis, in addition to its interaction with PCNA [64]. In this study, we put forth the hypothesis that RSF1 functions as an additional regulatory protein in the recruitment of PARG. Furthermore, we demonstrated that the RSF1 C1 mutant, which impairs 53BP1 condensate formation, also exhibited delayed PARG recruitment (Figure 4B) and prolonged PAR chains (Figure 3C) at DNA lesions. It is of interest to note that the introduction of PARP inhibitors in these cells resulted in the restoration of 53BP1 optoDroplet formation, which indicates that complete PAR removal is important for 53BP1 condensates (Figure 3E). Therefore, our findings provide compelling evidence to support the hypothesis that the transient compartmentalization of damaged DNA by PARP and FUS is essential for the sequential formation of 53BP1 liquid condensates within the compartment of damage chromatin.

A recent study revealed that PARP1 co‐condensates with DNA at DSB ends and PARylated at its C terminal BRCT domain, which in turn promotes the recruitment of both PARG and FET proteins. The multivalent and electrostatic interaction of negatively charged PAR chains with positively charged RBD domains in FUS stabilize these condensates while excluding 53BP1 [65]. Upon activation of PARG, PAR chains are degraded, leading to the dissolution of FUS condensates and facilitating the subsequent formation of 53BP1 foci, which also exhibit LLPS properties [32, 56, 66]. Subsequent dissociation by PARP1 autoPARylation from DSB ends permits access for downstream DNA repair proteins. In this context, RSF1 may be recruited via interaction with PARP1 and subsequently PARylated. Negatively charged PAR on RSF1 can cooperate with PARP1 exclusion from DSB ends and timely degradation by recruiting PARG, ultimately allowing the recruitment of 53BP1 at DSB ends. These data explain the possibility that RSF1 C1 mutant, which fails to efficiently interact with PARG, may aberrantly associate with FET proteins, sequester PAR chains, and block PARG recruitment. This would interfere with proper PAR clearance and thereby delay or prevent the transition to 53BP1 condensates. Our data also demonstrated that overexpression of FUS or PARG depletion, both of which increase PAR levels, significantly suppressed 53BP1 OptoDroplet formation (Figure 4C and Figure S4E). The data support the previous finding showing that expression of EWS is able to suppress 53BP1 accumulation at damaged chromatin [32]. Together, our findings support a model in which RSF1 facilitates PARG‐dependent PAR clearance at DNA damage sites, thereby promoting the subsequent assembly of 53BP1 condensates at sites of DNA damage, which are linked to the p53‐mediated gene transcription (Figure S7).

## 4 Experimental Section/Methods

### 4.1 Cell Culture

All experiments were conducted using the human osteosarcoma cell line U2OS. Cells were cultured in high‐glucose DMEM (WELGENE) containing 10% heat‐inactivated fetal bovine serum (Gibco) and 1X Antibiotic‐Antimycotic (Gibco), which includes penicillin (100 units mL^−1^), streptomycin (100 µg mL^−1^), and amphotericin B (0.25 µg mL^−1^). Cultures were maintained at 37°C in a humidified incubator with 5% CO_2_. Mycoplasma contamination was routinely assessed using the MycoFluor Mycoplasma Detection Kit (Thermo Fisher Scientific), and only verified negative lines were used in downstream assays.

### 4.2 Cloning and Plasmids

RSF1–mCherry–Cry2 fusion constructs (wild‐type and domain‐specific mutants such as C2) were generated via Gibson assembly. RSF1 fragments were PCR‐amplified using primer pairs listed in Table S2 and inserted into the linearized Cry2‐mCherry vector backbone. PCR products were digested with DpnI to remove template DNA, gel‐purified, and then assembled using isothermal Gibson reaction, followed by transformation into DH5α‐competent E. coli. Constructs encoding full‐length and truncated variants of 53BP1 were cloned using a similar strategy. CRY2‐mCherry‐FUSN and CRY2‐mCherry‐53BP1 WT and W1495A constructs were generously provided by Dr. Matthias Altmeyer. SFB‐RSF1 expression vectors were obtained from Dr. Sunwoo Min (Department of Biochemistry, Chungnam National University). The GFP‐RSF1 constructs were generated previously and described in Min et al., Cell cycle, 2014 (*44*). An NLS‐GFP expression plasmid (Addgene) was used as the backbone for generation of RSF1 truncation constructs. RSF1‐N2 (amino acids 1–871) and RSF1‐N3 (amino acids 1–973) fragments were amplified by PCR and cloned into the BamHI and ClaI sites of the NLS‐GFP vector (addgene 67652). Vector digestion and insert preparation were performed using standard molecular cloning procedures, followed by In‐Fusion‐mediated assembly. Recombinant plasmids were transformed into *E. coli* TOP10 cells, and positive clones were confirmed by Sanger sequencing before use in subsequent experiments. The RSF1 C2 7A mutant, in which the DEFDEAI sequence was substituted with alanine, was generated by site‐directed mutagenesis using the Stratagene/QuikChange mutagenesis protocol. Primers used for mutagenesis are listed in Table S2.

### 4.3 Generation and Validation of YFP‐53BP1 Knock‐In Cells

YFP‐53BP1 knock‐in HEK293 cells were generated by CRISPR/Cas9‐mediated homology‐directed repair. Briefly, the YFP coding sequence was inserted in‐frame at the N‐terminus of the endogenous TP53BP1 locus using an HDR donor containing YFP flanked by 5′ and 3′ homology arms. Cas9/sgRNA plasmids targeting the genomic region around the TP53BP1 start codon were co‐transfected with the HDR donor into HEK293 cells. YFP‐positive cells were isolated by fluorescence‐activated cell sorting, and single‐cell clones were established by limiting dilution. A single‐cell clone expressing YFP‐tagged 53BP1 was selected and used for live‐cell imaging experiments. Additional technical details for the generation of the YFP‐53BP1 knock‐in cell line are available from the corresponding author upon reasonable request.

### 4.4 RNA Interference, Plasmid Transfections

The siRNAs were transfected with Lipofectamine RNAiMAX and incubated for 72hr. Plasmid transfections were done by using polyethylenimine (PEI, Polysciences) and harvested at 48 h after transfection. Sequences of siRNA are listed in Table S3.

### 4.5 Immunoblotting

Proteins were mixed with 1× SDS sample buffer and denatured through heat treatment. Equivalent amounts of each lysate were loaded onto 4%–20% gradient SDS‐polyacrylamide gels for electrophoretic separation. Following gel electrophoresis, proteins were transferred to nitrocellulose membranes using a conventional wet transfer apparatus. Membranes were blocked for one hour at ambient temperature in TBST supplemented with 1 percent bovine serum albumin to prevent nonspecific interactions. Blots were incubated overnight at 4 degrees Celsius with primary antibodies, washed, and then treated with horseradish peroxidase‐conjugated secondary antibodies (Bio‐Rad) for one hour at room temperature. Signals were developed using enhanced chemiluminescence reagents (GE Healthcare), and band intensities were detected with a digital imaging system. The primary antibodies used are listed in Table S4.

### 4.6 Pull‐Down Assay

HEK 293T cells were lysed with NETN buffer (50 mM Tris, pH 8.0, 150 mM NaCl, 1% Nonidet P‐40, 50 U mL^−1^ MNase, 50 U mL^−1^ Benzonase) including proteases and phosphatase inhibitor cocktail (Roche) and incubated for 20 min at room temperature. Cell lysate was centrifuged at 13 000 rpm for 15 min at 4°C. For pull down assay of SFB‐tagged proteins, the supernatant was incubated with Streptavidin Sepharose High Performance affinity resin (GE Healthcare) for 2 h at 4°C and washed four times with NETN buffer. The washed precipitates were boiled with 2× sample buffer and used for western blotting assay.

### 4.7 Immunoprecipitation

U2OS RSF1 KO cells were lysed with NETN buffer (50 mM Tris, pH 8.0, 150 mM NaCl, 1% Nonidet P‐40 and 5 mM EDTA) including proteases and phosphatase inhibitor cocktail (Roche). Cell lysate was sonicated using EpiShear Probe Sonicator (Active motif) and centrifuged at 13 000 rpm for 15 min at 4°C. For immunoprecipitation, the supernatant was incubated with primary antibody for overnight at 4°C. Next day, the immunoprecipitates were captured by incubation with Protein A Sepharose Fast‐Flow (GE Healthcare) for 1 h and the beads were washed four times with NETN buffer. The washed precipitates were boiled with 2× sample buffer and used for western blotting assay.

### 4.8 OptoDroplet Assay

Cells were transfected with IDR‐Cry2‐mCherry plasmids using polyethyleneimine (PEI, Polysciences) for 48 h before imaging. Droplet formation was induced with blue light pulse at 488 nm laser (50% laser power) every 2 s during the imaging process, and images were captured every 2 s. Images visible under a Nikon A1R confocal microscope (Nikon) with a 64x oil objective were captured.

### 4.9 Fluorescence Recovery After Photobleaching (FRAP)

For laser bleaching, U2OS RSF1 WT and U2OS RSF1 KO cells were seeded onto 35‐mm glass‐bottom dishes (SPL, Korea). Cells were transfected with 53BP1‐EGFP for 48 h and subsequently treated with NCS to induce 53BP1 foci formation. Regions of interest (ROIs) were selected within pre‐existing 53BP1 foci, and the selected regions were photobleached using the FRAP module of a Nikon A1R confocal microscope (Nikon) equipped with a 488‐nm laser. Bleaching was performed at 100% laser power, and images were collected every 1 s. The entire puncta or part of the puncta inside was photobleached during time‐lapse imaging. Images were further processed, and the fluorescence intensity in the photobleached region was measured using NIS‐element AR software and values were normalized to pre‐bleach time points.

### 4.10 53BP1 Fusion and Fission Events

Fusion and fission events of 53BP1 condensates were monitored using genetically engineered 293 cells expressing endogenously tagged YFP‐53BP1. Live‐cell images were acquired in confocal mode using a Leica STELLARIS 8 microscope at 5 min intervals for up to 1 h after DNA damage induction by treating cells with NCS (Neocarzinostatin, Sigma, N9162).

### 4.11 Laser Microirradiation and Immunofluorescence

For laser microirradiation, U2OS RSF1 WT and U2OS RSF1 KO cells were seeded onto 35 mm round bottom dishes (SPL, Korea). Cells were transfected with DNA for 48–72 h and incubated with 10 µM 5‐bromo‐2 deoxyridine (BrdU) for 24 h before laser‐induced DNA damage. Single‐ and double‐strand DNA breaks were induced by laser microirradiation using 405 nm laser in Nikon A1R confocal microscope (Nikon). Cells were subjected to laser‐induced DNA damage with two different conditions [3 s/ 32 line for live cell imaging and 1 s/32 line for fixed cell imaging] using 60× oil objective. For fixed cell imaging, cells were fixed with 2% (v/v) solution formaldehyde solution (Sigma) in PBS at room temperature after damaged for indicated times. Cells were blocked for 30 min at room temperature in blocking solution (1% BSA in PBS). Cells were incubated with primary antibodies for overnight at 4°C. Next day, secondary antibodies (Invitrogen, Carlsbad, CA, USA) were incubated for 1 h at room temperature and mounted with VECTASHIELD with DAPI (Vector Laboratories). The primary antibodies used are listed in Table S4.

### 4.12 Immunofluorescence for DNA Damage‐Induced Foci Formation

For analysis of damage‐induced 53BP1 foci formation, U2OS RSF1 WT and RSF1 KO cells were plated on 35 mm glass bottom dishes (SPL, Korea). Cells treated with 200 ng mL^−1^ Neocarzinostatin (NCS, Sigma–Aldrich) for the indicated time points. Cells were fixed with 4% PFA in PBS for 15 min at room temperature (RT) following NCS treatment. Fixed cells were washed three times with PBS and permeabilized with 0.5% TritonX‐100 in PBS for 15 min at RT. Non‐specific signals were blocked using blocking solution (PBS containing 1% BSA and 0.5% TritonX‐100) at RT for 1 h. After then, primary antibodies diluted in the blocking solution were overnight incubated at 4°C. Following day, Alexa Fluor‐conjugated secondary antibodies were incubated for 2 h at RT and washed three times with PBS. Cells were counterstained with 1 µg mL^−1^ DAPI (Sigma–Aldrich) diluted in PBS at RT for 10 min, washed with PBS, and then mounted onto glass using VECTASHIELD mounting solution (Vector laboratories). Detailed information of the antibodies used in this study are described in Table S4.

### 4.13 Recombinant Protein Expression and Purification From Sf9 Insect Cells and *E. Coli*

To characterize the biochemical properties of RSF1 and its variants, we employed both insect and bacterial expression systems for recombinant protein production. Full‐length and domain‐specific RSF1 constructs were inserted into pDEST20 and pDEST15 plasmids to produce GST‐tagged proteins. For baculovirus‐based expression, pDEST20 plasmids were introduced into DH10Bac E. coli to generate bacmid DNA, which was subsequently transfected into Sf9 cells using Cellfectin II (Invitrogen). After a 72 h incubation period, cells were collected and lysed in NETN buffer (50 mM Tris‐HCl pH 8.0, 150 mM NaCl, 1 mM EDTA, 0.5% NP‐40) supplemented with protease inhibitors (Roche). Following centrifugation at 14 000 rpm for 15 min at 4°C, cleared lysates were incubated with Glutathione‐Sepharose 4B (GE Healthcare) at 4°C with constant agitation for 2 h. After thorough washing, bound proteins were eluted using HEPES‐based buffer containing reduced glutathione. The eluted samples were quantified using the Bradford protein assay (Bio‐Rad). For bacterial expression, RSF1 fragments were subcloned into pDEST15 or pDEST15‐3C and transformed into *E. coli* BL21(DE3) pLysS cells. Protein induction was carried out overnight at 18°C using 0.5 mM IPTG. Subsequent purification followed the same procedure as above. For tag removal, samples from the pDEST15‐3C system were treated with HRV 3C protease in cleavage buffer at 4°C for 24 h and passed over glutathione resin to eliminate residual tags and protease. Protein purity was verified by SDS‐PAGE and Coomassie staining. Final preparations were aliquoted, rapidly frozen in liquid nitrogen, and stored at −80°C for use in downstream assays such as PARylation and phase separation analyses.

### 4.14 Poly‐ADP‐Ribosylation In Vitro Assay

Purified recombinant GST‐RSF1 WT and mutant DNA protein were subjected to SDS‐PAGE and transferred onto a nitrocellulose membrane. And the membranes were incubated with ribosylation buffer (Biotin‐NAD, 1X PARP cocktail, 1X PARP buffer, 1X Activated DNA, PARP1 enzyme) for 1 h at room temperature. After washing in PBST and then incubated with 5% SDS buffer for strip off non‐covalent PAR polymer. After washing in PBST, PAR‐binding proteins were detected with PAR antibody.

### 4.15 Quantitative Real‐Time PCR

Total RNA was extracted with TRIzol (Invitrogen), and cDNA was synthesized using a First Strand Synthesis Kit (Origen). Amplification of target transcripts was performed using SYBR‐based qPCR (Bio‐Rad) and analyzed via CFX Manager software. The primer sequences were listed in Table S5.

### 4.16 Reporter Gene Activity Measurement Using Luciferase Assay

To measure the transcriptional activity of p53 in the presence or absence of RSF1, U2OS cells were seeded in 24‐well plates and co‐transfected with the p53‐responsive PG13‐luciferase reporter, the mutant MG15‐luc reporter (containing non‐functional p53 response elements), and RSF1 expression plasmids using Lipofectamine 2000 (Invitrogen). After 48 h, cells were treated with 34 µM etoposide for an additional 12 h to induce DNA damage and p53 activation. Luciferase activity was determined using the Dual‐Luciferase Reporter Assay System (Promega) according to the manufacturer's protocol. Firefly luciferase signals from PG13 or MG15 constructs were normalized to Renilla luciferase, which was co‐transfected as an internal control under a constitutive promoter. All experiments were performed in triplicate, and statistical analyses were conducted using GraphPad Prism software. The results were expressed as firefly/Renilla ratios to reflect relative p53‐dependent transcriptional activity.

### 4.17 Chromatin Immunoprecipitation Assay

For Chromatin Immunoprecipitation (ChIP) analysis, cells were treated with 34 µM etoposide for 12 h to induce DNA damage. Crosslinking of DNA‐protein complexes was achieved using 1% formaldehyde for 20 min at room temperature. Cells were then lysed on ice using the SDS lysis buffer provided in the ChIP Assay Kit (Millipore, 17–295), supplemented with protease and phosphatase inhibitors (Thermo Scientific). Chromatin was sheared using a Bioruptor sonicator (Diagenode) and cleared by centrifugation. The resulting supernatant was diluted with ChIP dilution buffer and incubated overnight at 4°C with specific antibodies. On the following day, immune complexes were captured by incubation with Protein A agarose beads pre‐blocked with salmon sperm DNA (Millipore) for 2 h at 4°C. Bound chromatin was washed and eluted using a solution containing 1% SDS and 0.1 M NaHCO_3_. To reverse crosslinks, eluates were treated with 5 M NaCl and heated at 65°C for 4 h, followed by proteinase K digestion at 45°C for 1 h. DNA was purified using the Nucleospin PCR Cleanup Kit (Macherey Nagel) and analyzed by quantitative PCR using Maxima SYBR Green qPCR Master Mix (Thermo Scientific). The primer sequences were listed in Table S6.

### 4.18 Protein‐Protein Interaction Modeling via AlphaFold‐Multimer

To predict the three‐dimensional structural complexes of PARG with its interaction partners (RSF1, C1, and C2, respectively), in silico structural modeling was performed using ColabFold [67] implementing AlphaFold‐Multimer [68]. The full‐length amino acid sequences of human PARG, RSF1, C1, and C2 were retrieved from the UniProt database. Multiple sequence alignments (MSAs) were generated using the MMseqs2 API against the UniRef100 and environmental databases. For each complex heterodimer, five independent prediction models were generated. The resulting structural models were ranked based on the predicted local distance difference test (pLDDT) and predicted alignment error (PAE) scores. The top‐ranked structure (Rank 0), which exhibited the highest confidence score, was selected as the representative model for subsequent structural analysis.

### 4.19 Molecular Visualization and Analysis

The coordinates of the selected top‐ranked models were exported in PDB format. The three‐dimensional structures and interaction interfaces of the PARG complexes were rendered, visualized, and analyzed using the web‐based Mol* Viewer (https://molstar.org/viewer/) [69]. Distinct color codes were applied to differentiate PARG from its partner proteins. To quantitatively assess the interaction interfaces, the contact residues of RSF1, C1, and C2 located within a 4.5Å cutoff distance from any atom of PARG were identified and extracted using the command‐line selection tools in UCSF ChimeraX [70].

### 4.20 In Silico Molecular Docking Simulation

To evaluate and compare the binding affinities of wild‐type C2 and its alanine‐substituted mutants toward PARG, molecular docking simulations were performed using the HADDOCK2.4 web server (https://wenmr.science.uu.nl/haddock2.4/) [70]. The initial three‐dimensional coordinates (PDB files) for PARG and C2 and mutant variants were generated via AlphaFold3 structural predictions [71]. The docking runs were driven by defining ambiguous interaction restraints (AIRs) based on the manually identified proximal residues involved in the PARG‐C2 interface. Standard HADDOCK parameters were applied for the rigid‐body energy minimization, semi‐flexible refinement in explicit solvent, and final water refinement stages. The resulting docking poses were clustered based on the HADDOCK score, and the top‐ranked cluster for each run—representing the lowest‐energy conformation—was selected for further quantitative analysis. The interaction stability was assessed using the HADDOCK score, Van der Waals energy, electrostatic energy, and Buried Surface Area (BSA).

### 4.21 Clonogenic Survival Assay

U2OS RSF1 WT, RSF1 KO cells were transfected with GFP‐RSF1 WT, C1 or C2. Next day, cells were seeded (3000 cells/each well) on 6‐well plate (TPP). Next day, cells were treated with etoposide or phleomycin for indicated concentrations. Cells were incubated in a temperature‐controlled chamber (37°C, 5% CO2) for 10–14 days. Cells were fixed with purified methanol (Sigma) for 10 min and stained with 0.01% crystal violet for 20 min. Colony areas were measured and analyzed by ImageJ.

### 4.22 DNA Repair Assay

DR and EJ5 cells were seeded and, next day, cells were treated with the indicated siRNA and RSF1 mutants. After 24 h,*I‐Sce*I endonuclease was delivered to cells by Lipofectamine 2000 (Invitrogen). After 72 h, cells were trypsinized and centrifuged at 1500 × g for 5 min. Subsequently, cells were washed with PBS and resuspended in the same buffer. The cell suspension was then transferred to 1 mL Round Bottom Polystyrene Tube (Falcon) for FACS analysis. GFP‐positive cells were quantified using the FACSAria III (BD biosciences).

### 4.23 Image Quantification

All microscopy data were acquired using consistent settings on a Nikon A1R confocal microscope equipped with a 60× 1.4 NA oil objective. Image analysis was performed using either NIS‐Elements AR software (Nikon) or ImageJ (NIH). For laser‐induced damage assays, GFP fluorescence intensity within the irradiated region was measured and normalized against the average intensity across the entire nucleus. For optoDroplet experiments, condensate areas were quantified by detecting mCherry‐positive droplets formed by the Cry2‐fusion constructs. Droplets were identified in the mCherry channel as discrete objects with fluorescence intensity above the background signal. The same intensity threshold for droplet detection was applied to all samples within each experiment. The detected condensate area was calculated as the summed area of mCherry‐positive droplet objects per cell. Schematic diagrams in Figure S7 were prepared using BioRender.

### 4.24 Statistical Analysis

All statistical analyses were performed using GraphPad Prism 8 (GraphPad Software). Quantitative data are presented as mean ± standard error of the mean (SEM) from at least three independent biological experiments unless otherwise specified. The sample size (*n*) for each experiment is indicated in the corresponding figure legends. No data transformation was performed prior to statistical analysis, and no outliers were excluded. Comparisons between two groups were performed using an unpaired two‐tailed Student's t‐test unless otherwise specified. Comparisons among three or more groups were performed using one‐way analysis of variance (ANOVA) followed by Tukey's or Dunnett's multiple‐comparison tests, as appropriate. Statistical significance was defined as *p* < 0.05, with significance levels indicated as *p < 0.05, **p < 0.01, and ***p < 0.001. Protein band intensity was quantified using ImageJ (https://imagej.net/ij/).

## Author Contributions

**Y.H**. performed all experiments and carried out data analysis, with assistance from **Y.K**., **S.J**., **N.H**., **G.S**., and **Y.L**., and **J.‐H.L**. provided analytical and technical resources. **Y.W.C**., **H.‐C.K**., **H.C**., and **S.M**. conceived, designed, and supervised the research. **Y.H**., **H.C**., and **S.M**. wrote the manuscript. All authors contributed to data interpretation and reviewed the final version of the manuscript.

## Ethics Statement

This study did not involve human samples or animal experiments. Therefore, ethical approval and an approval number were not applicable.

## Conflicts of Interest

The authors declare no conflicts of interest.

## Supporting information




**Supporting File 1**: advs77271‐sup‐0001‐SuppMat.docx.

**Supporting File 2**: advs77271‐sup‐0002‐SuppMat.pdf.

**Supporting File 3**: advs77271‐sup‐0003‐SuppMat.pdf.

**Supporting File 4**: advs77271‐sup‐0004‐SuppMat.zip.

## Data Availability

The data that supports the findings of this study are available in the Supporting Information of this article.
