# Supplementary material for: RSF1‐Dependent PAR Turnover Promotes 53BP1 Liquid Condensate Formation at DNA Damage Sites
Source: Adv Sci (Weinh). 2026 Aug 24;13(63):e77271. doi: 10.1002/advs.77271 (PMC13502265; doi:10.1002/advs.77271)

Figure 1B

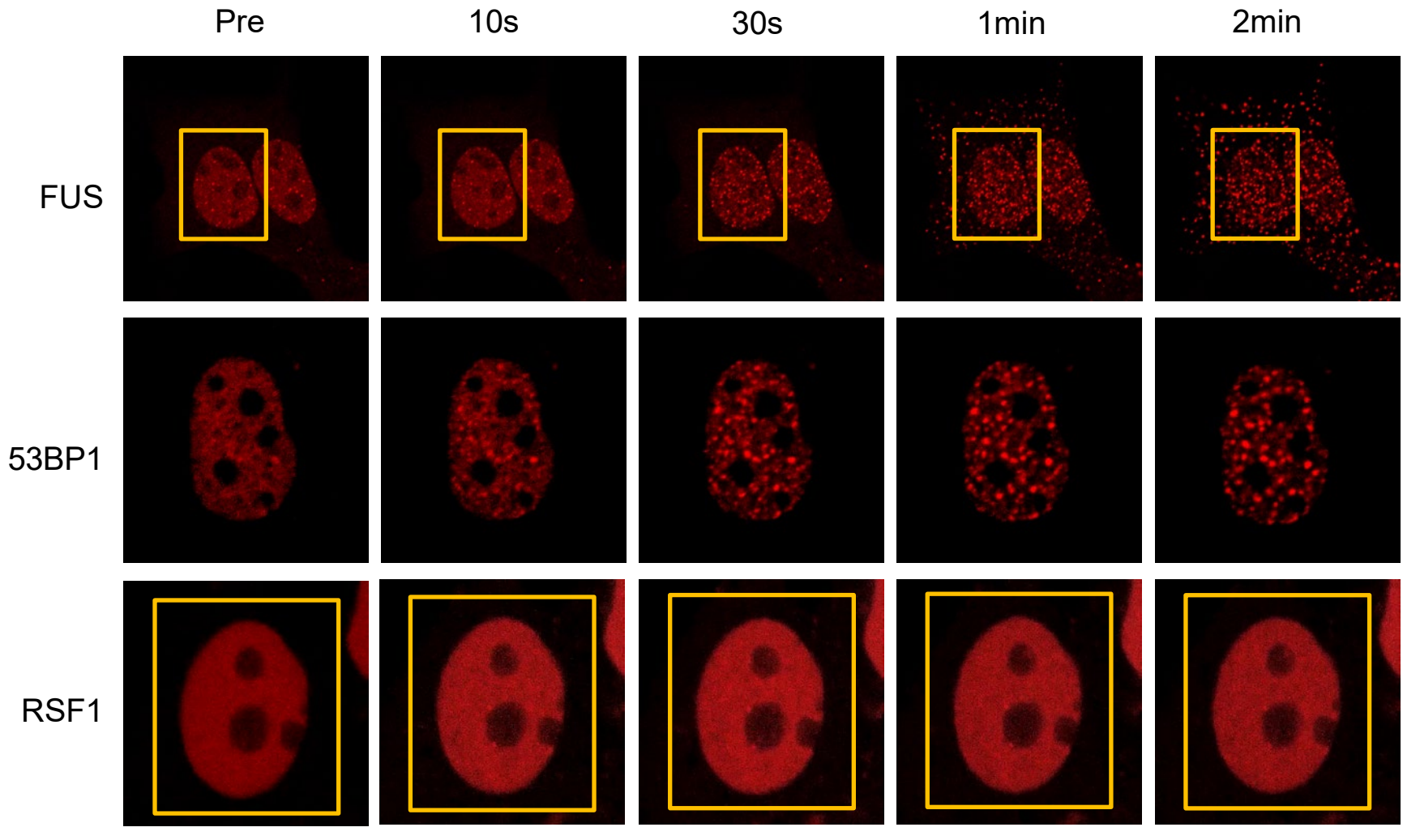

Figure 1C

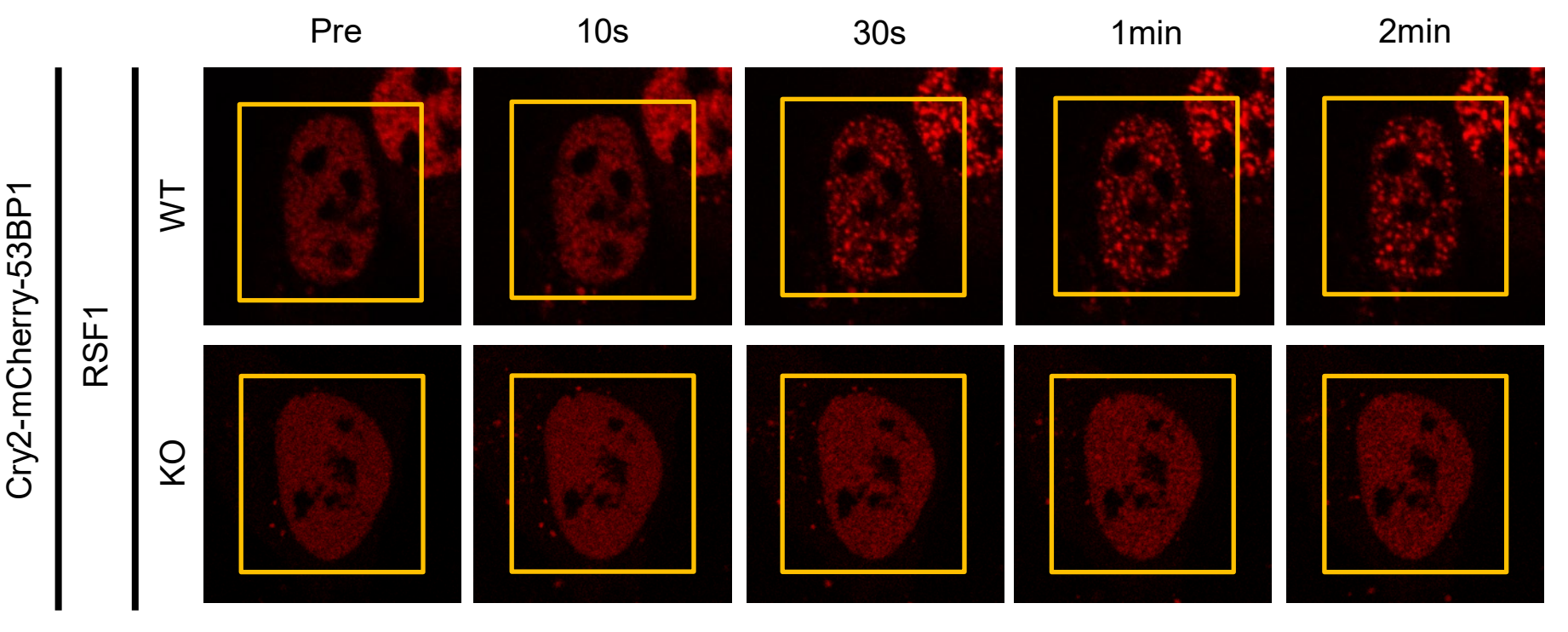

Figure 1D

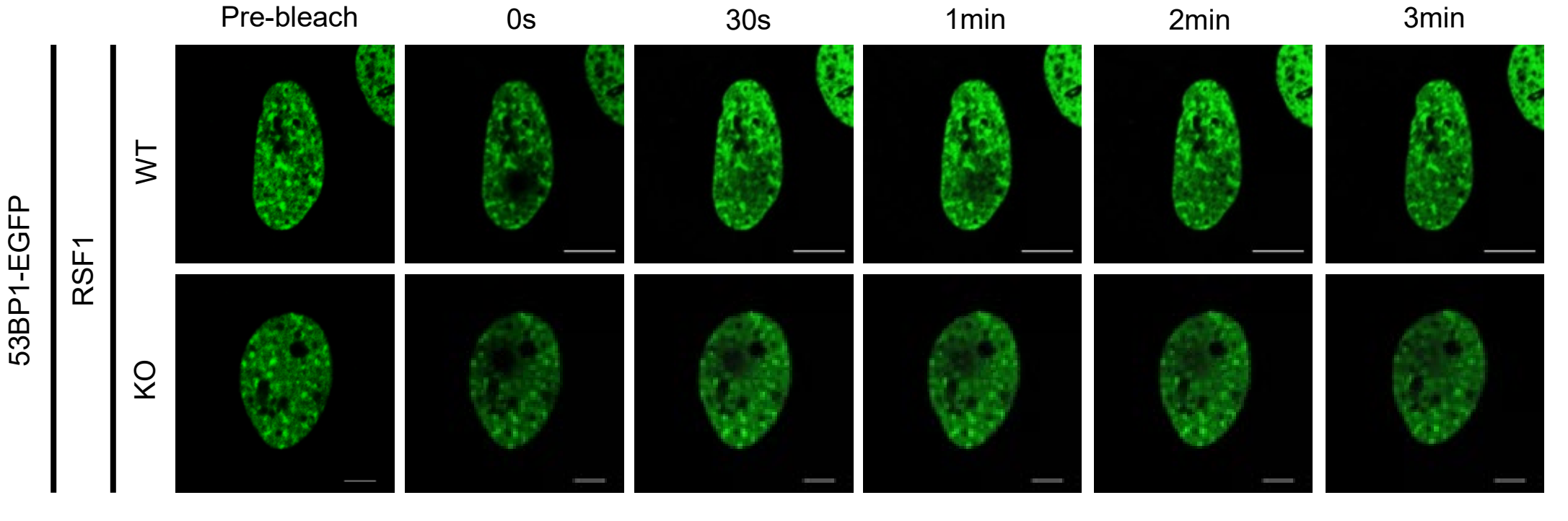

Figure 1E

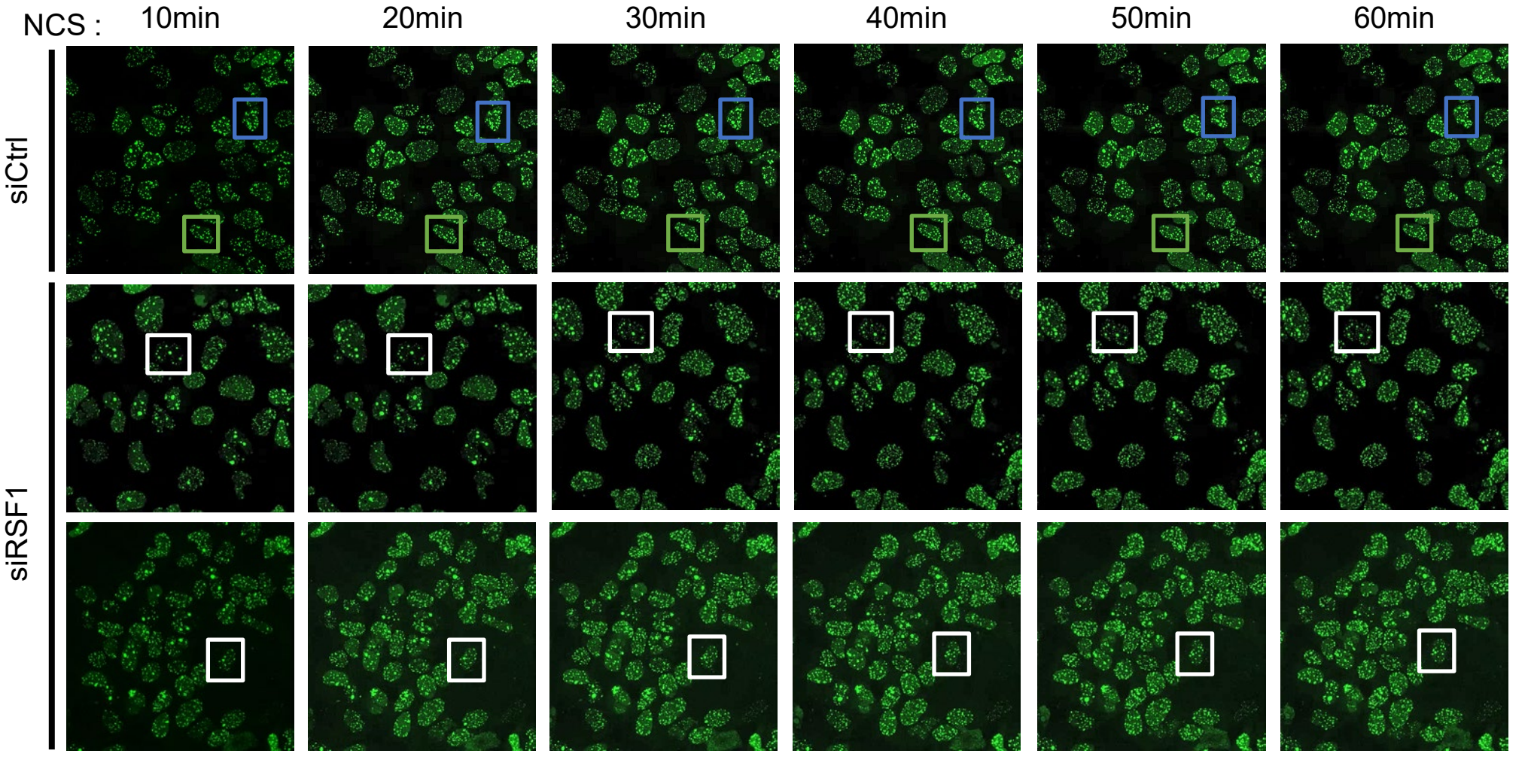

Figure 1F

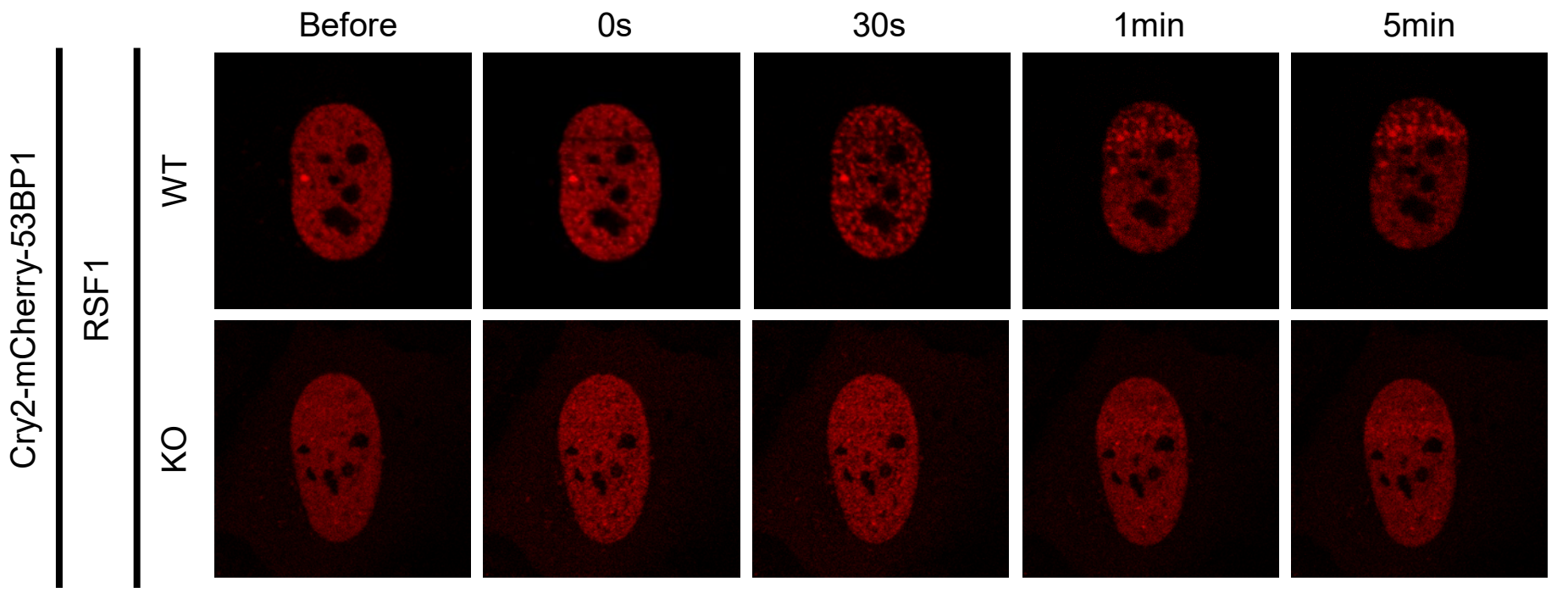

Figure 2B

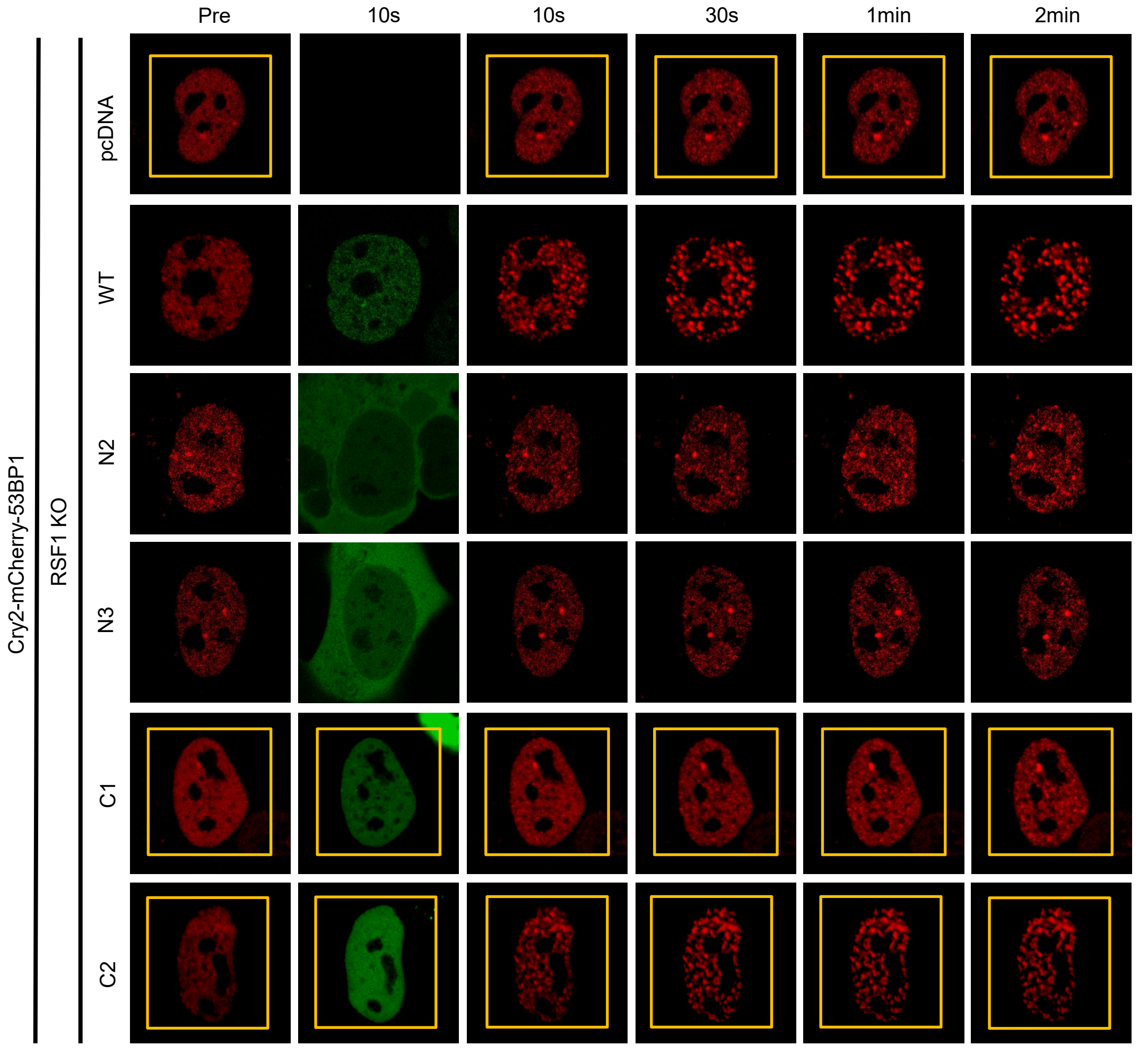

Figure 2C

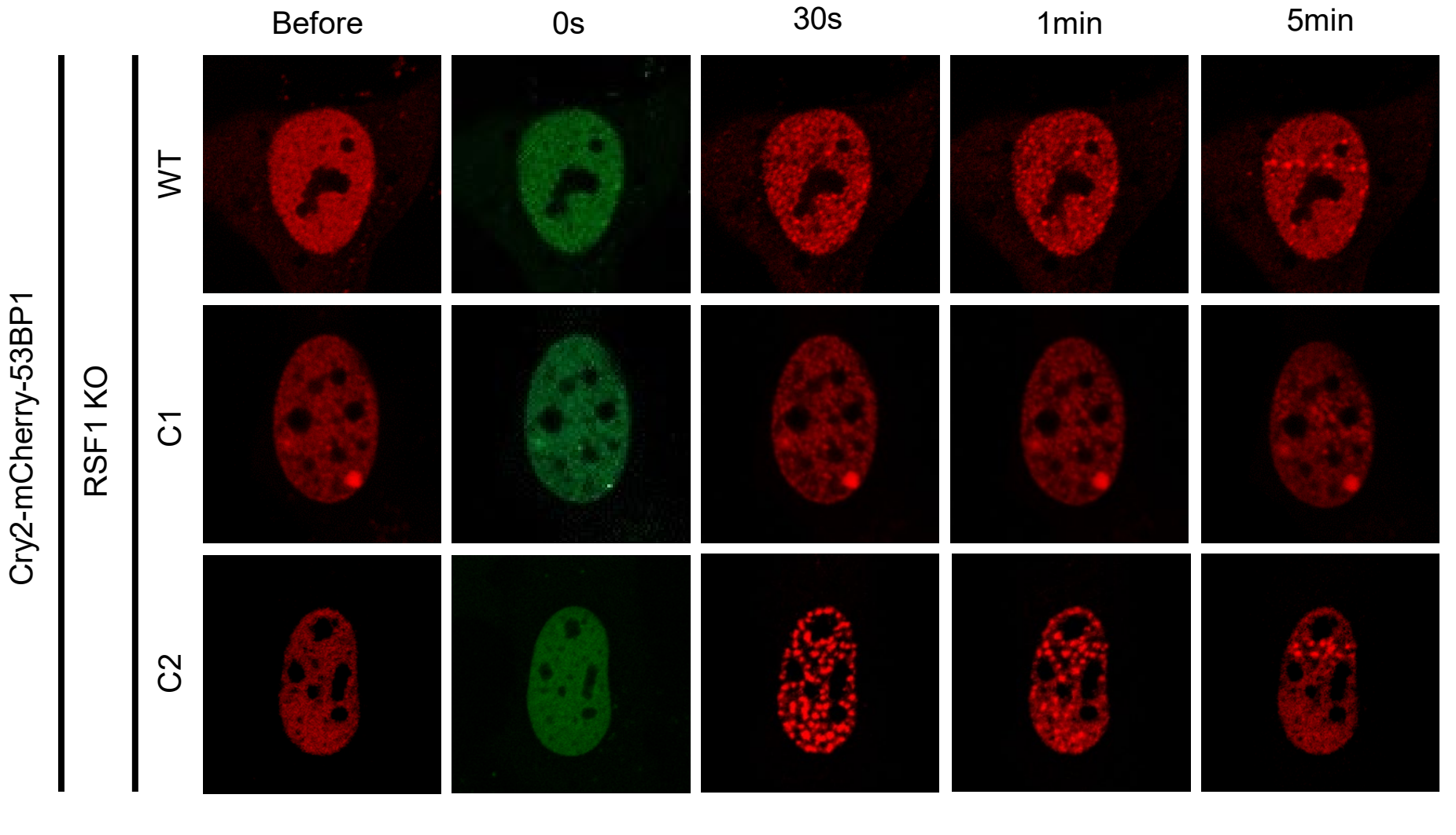

Figure 3C

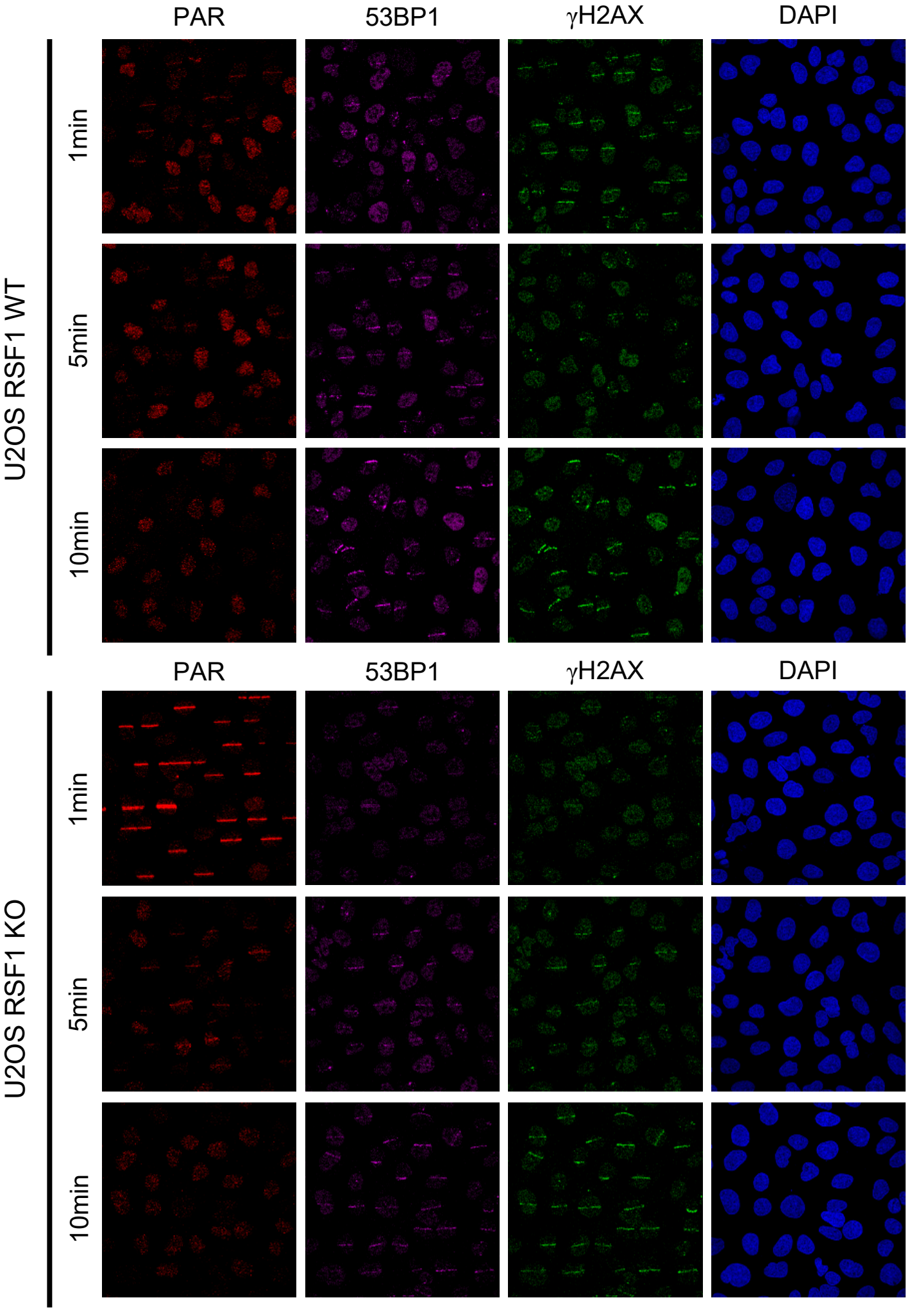

Figure 3D

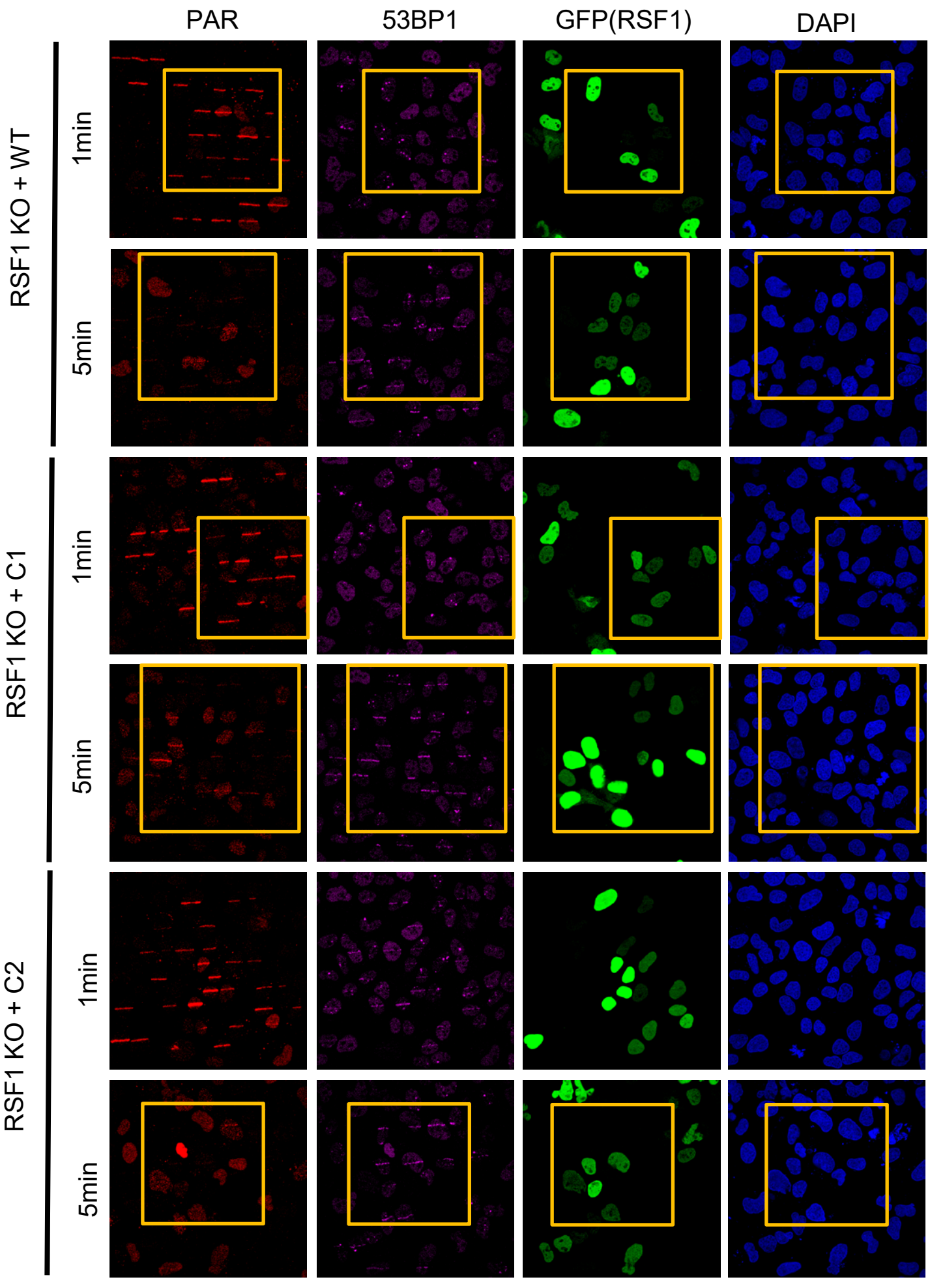

Figure 3E

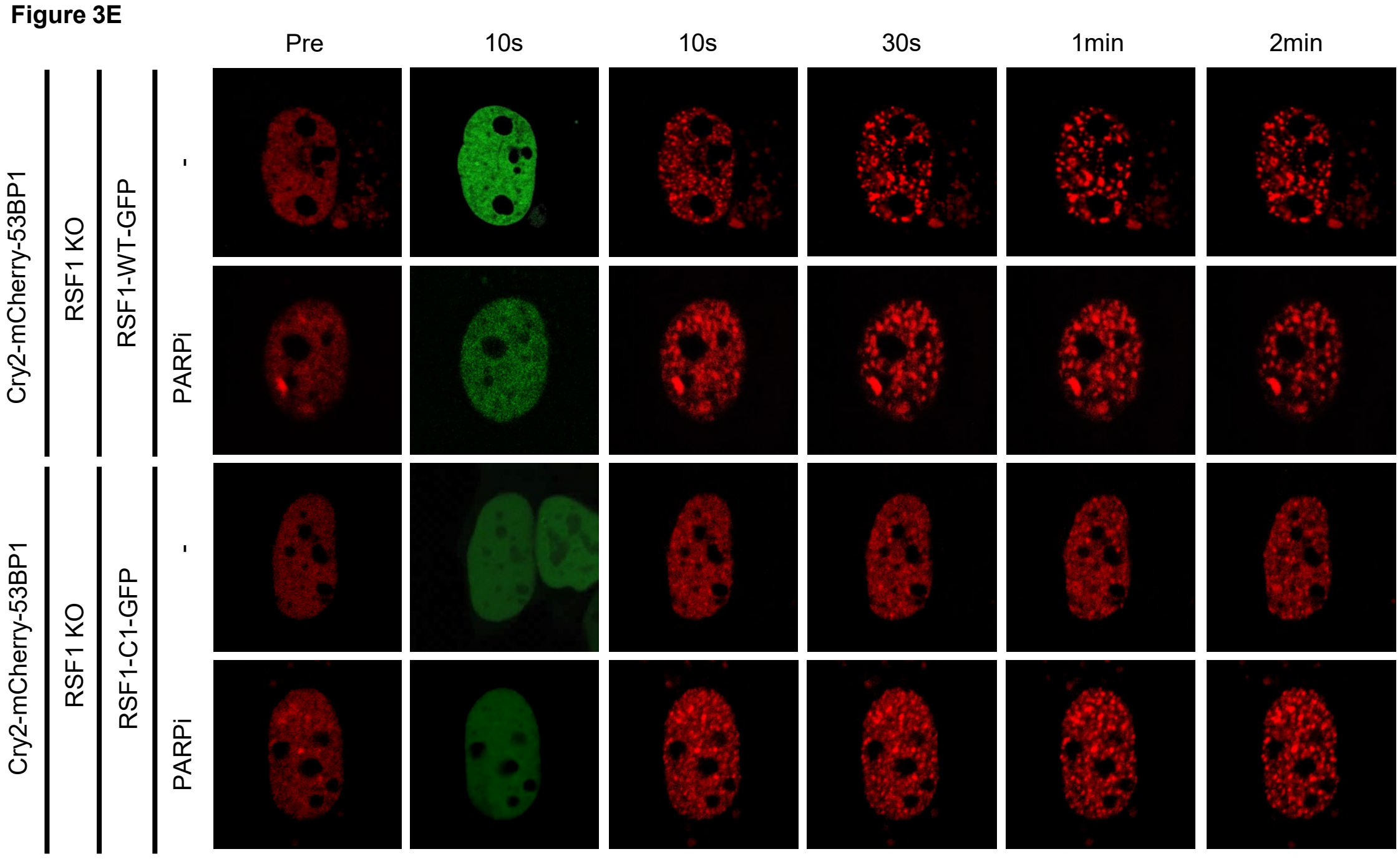

Figure 4A

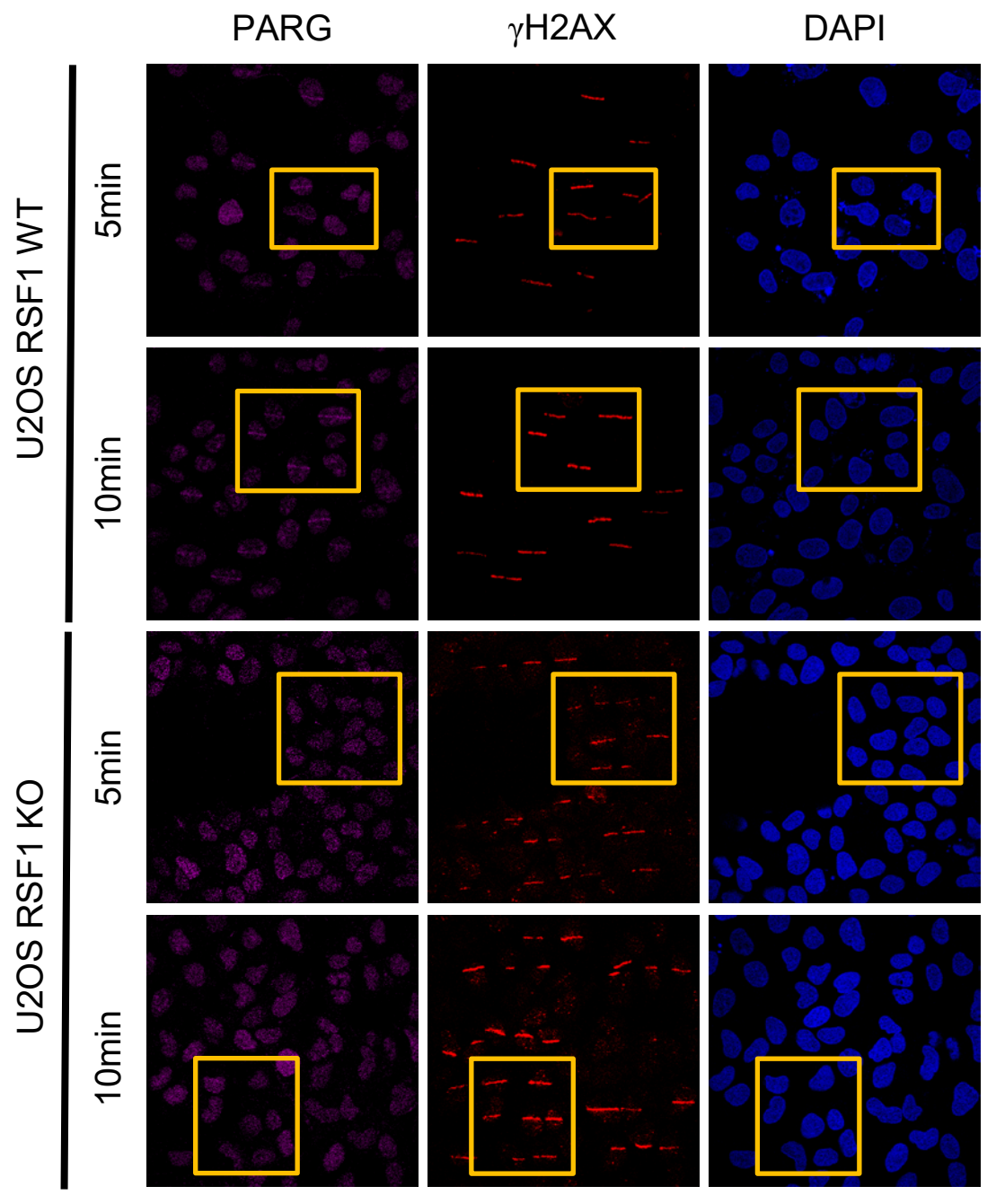

Figure 4B

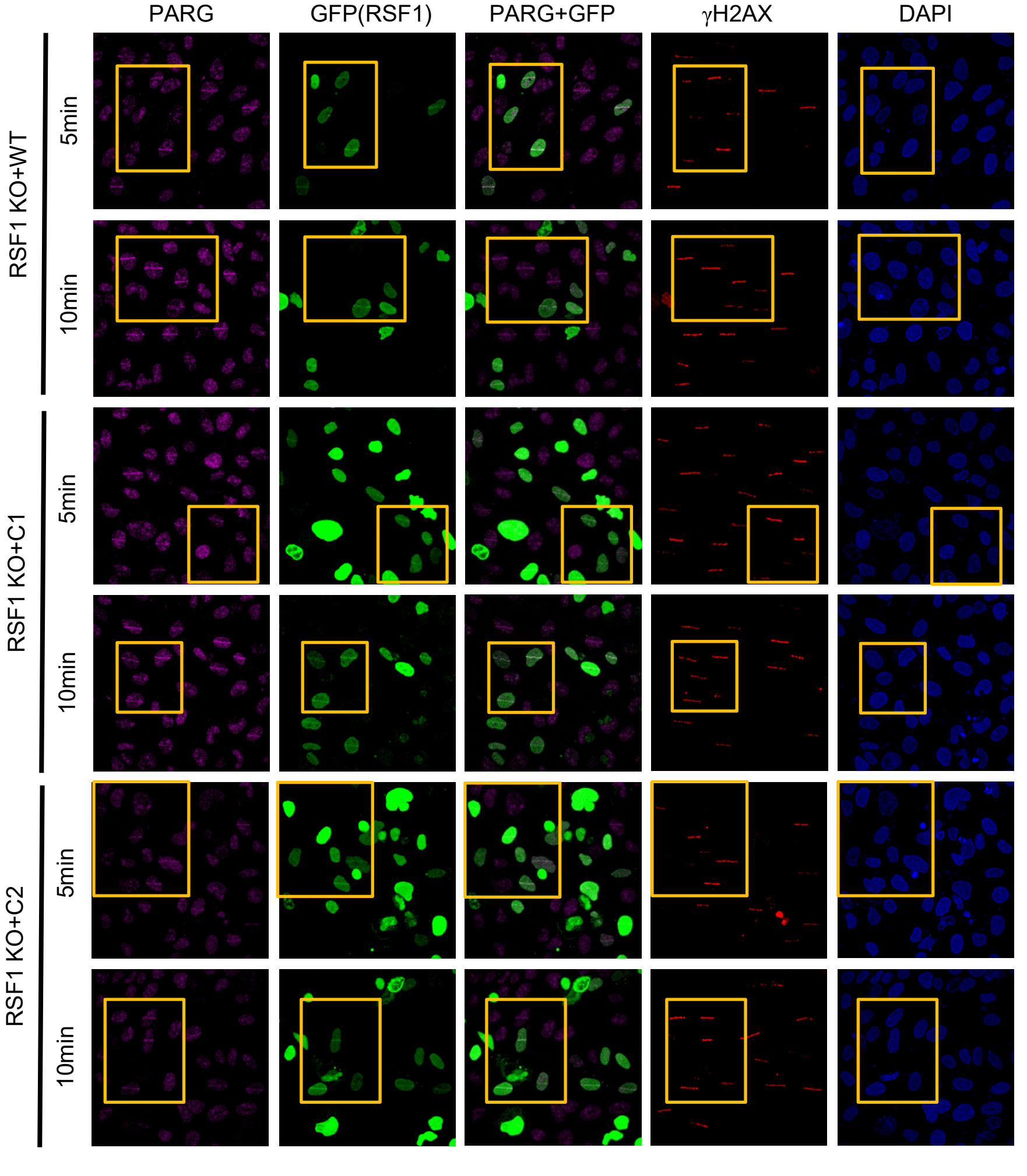

Figure 4C

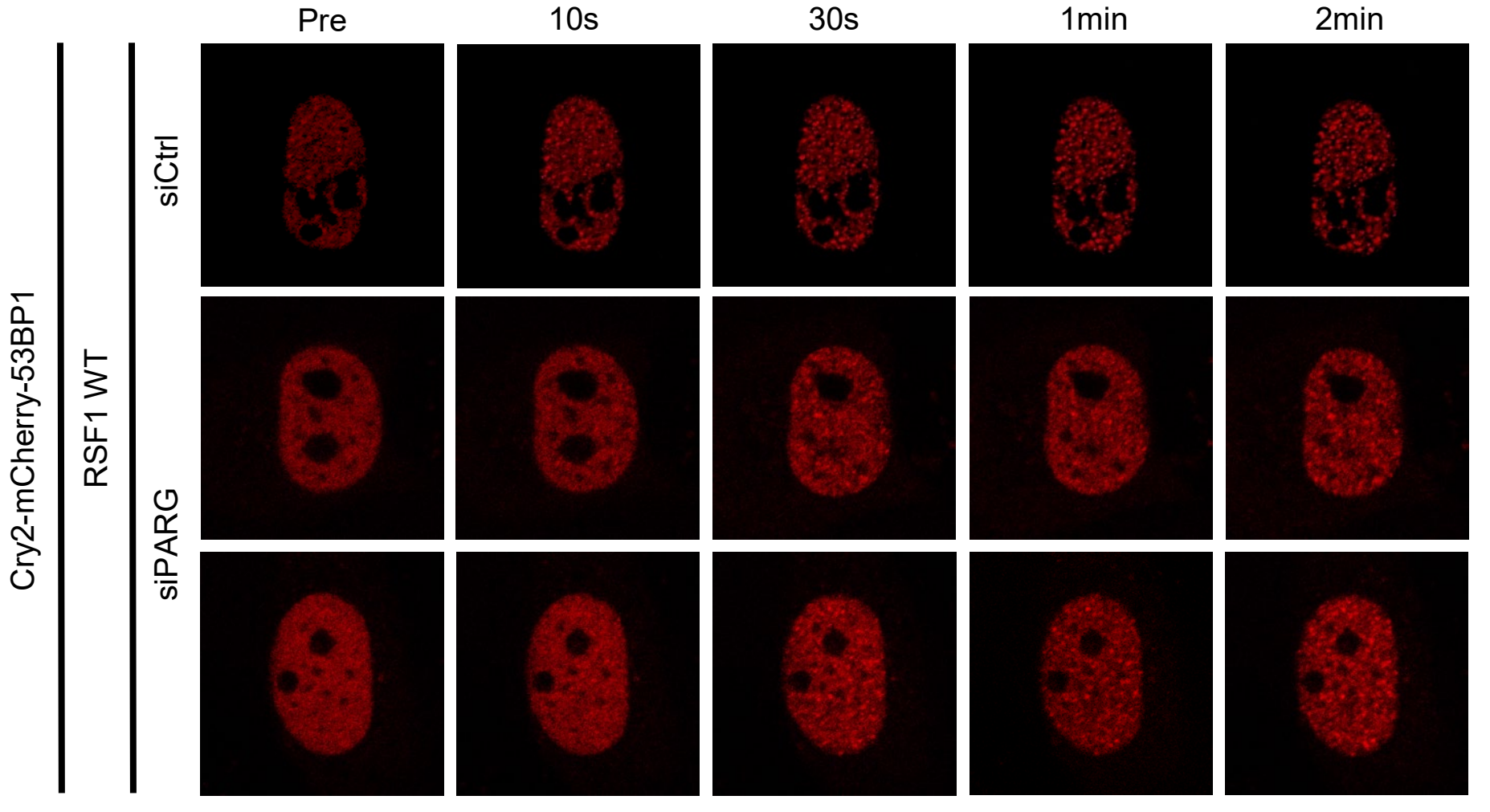

Figure 4E

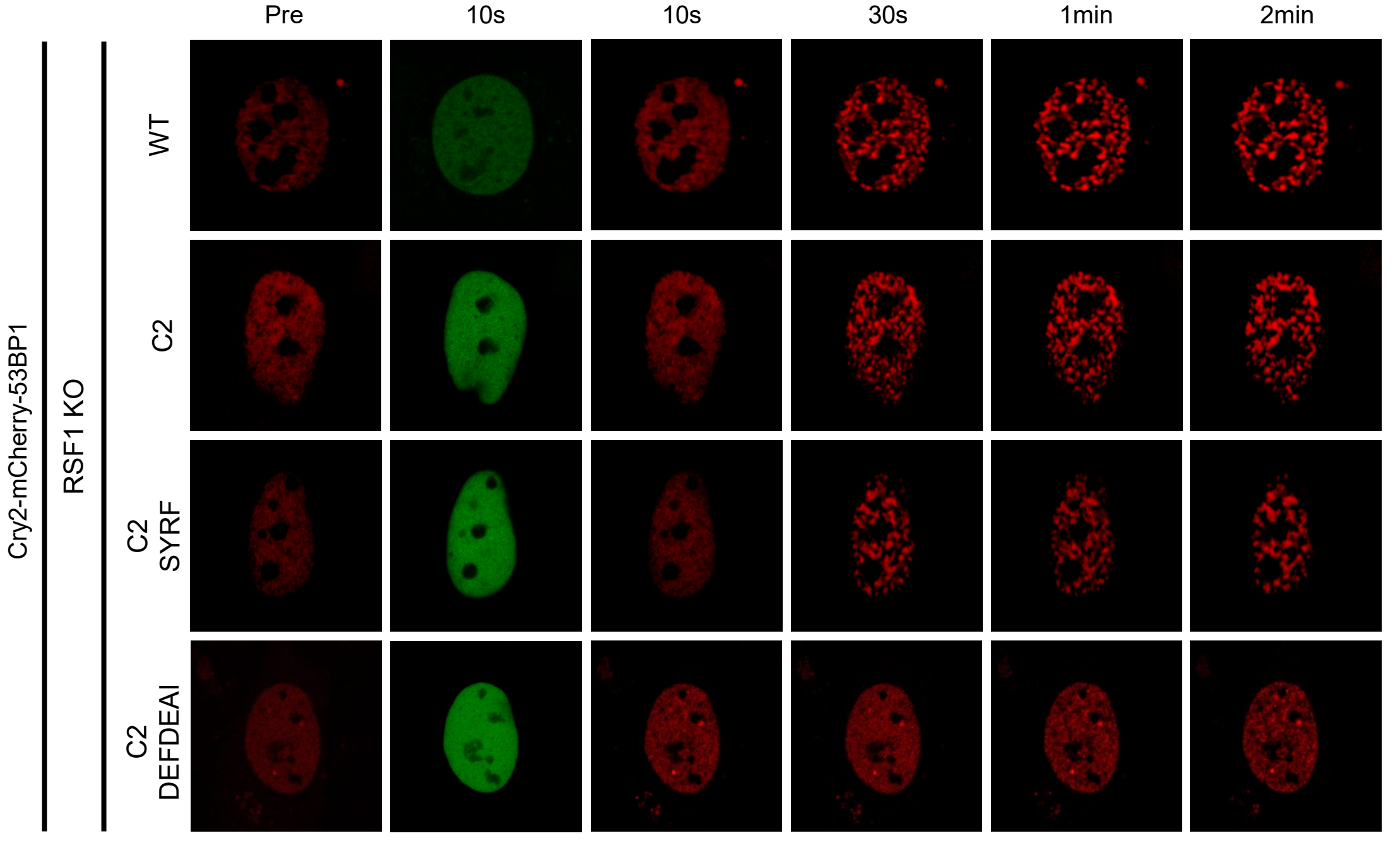

Figure 5C

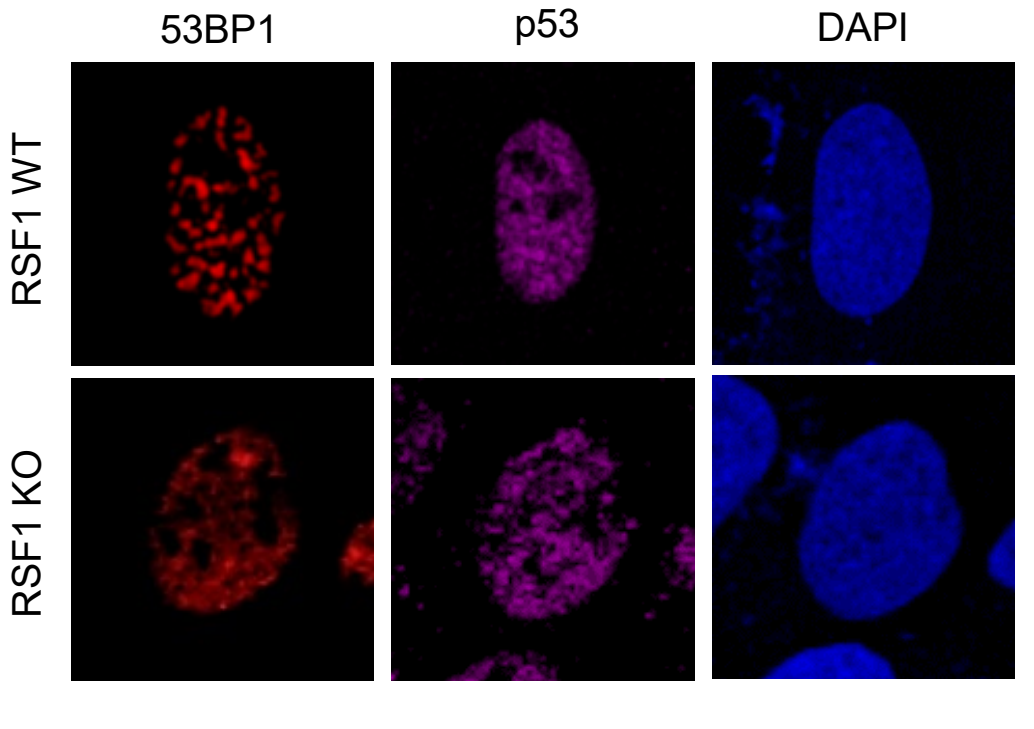

Figure S1C

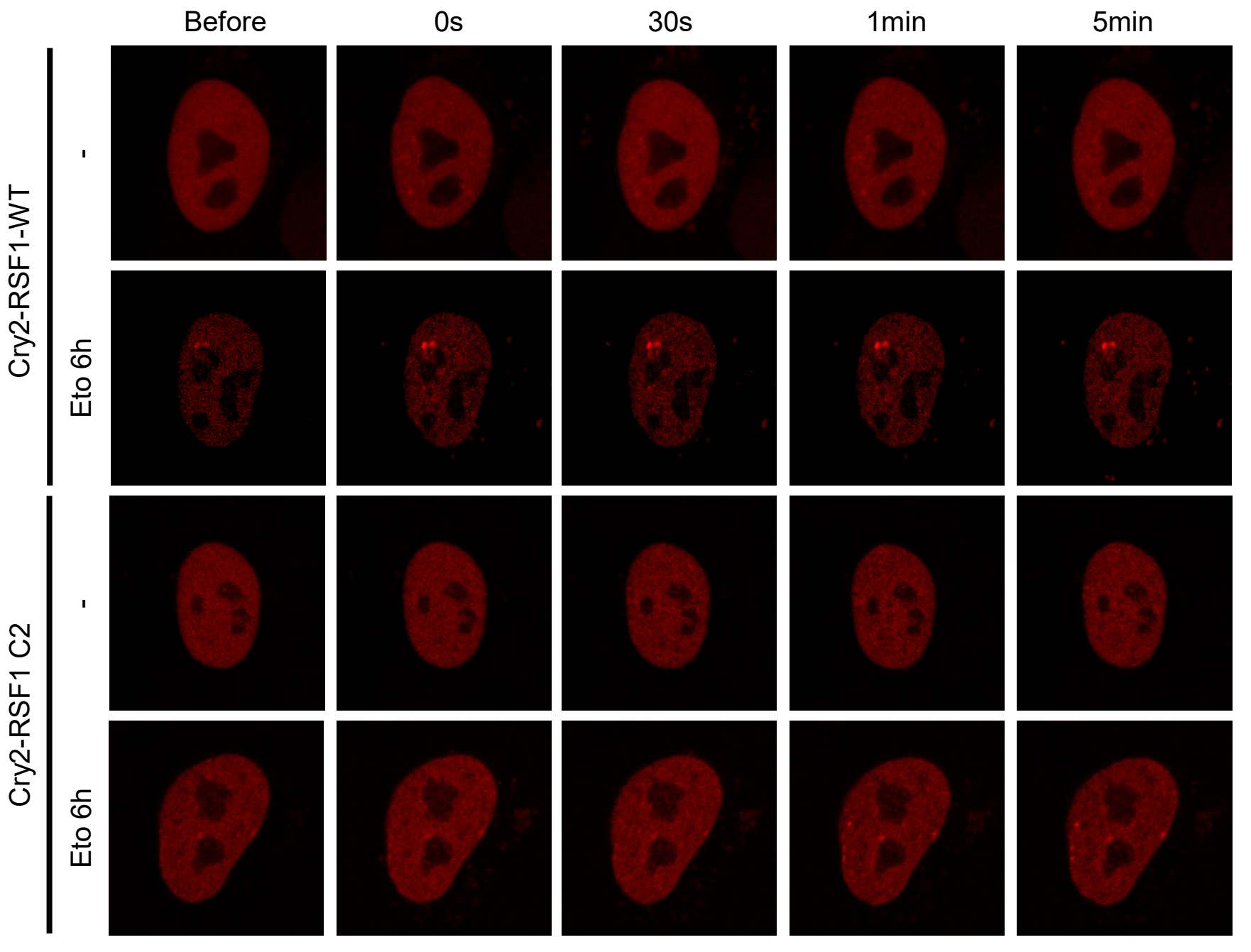

Figure S1D

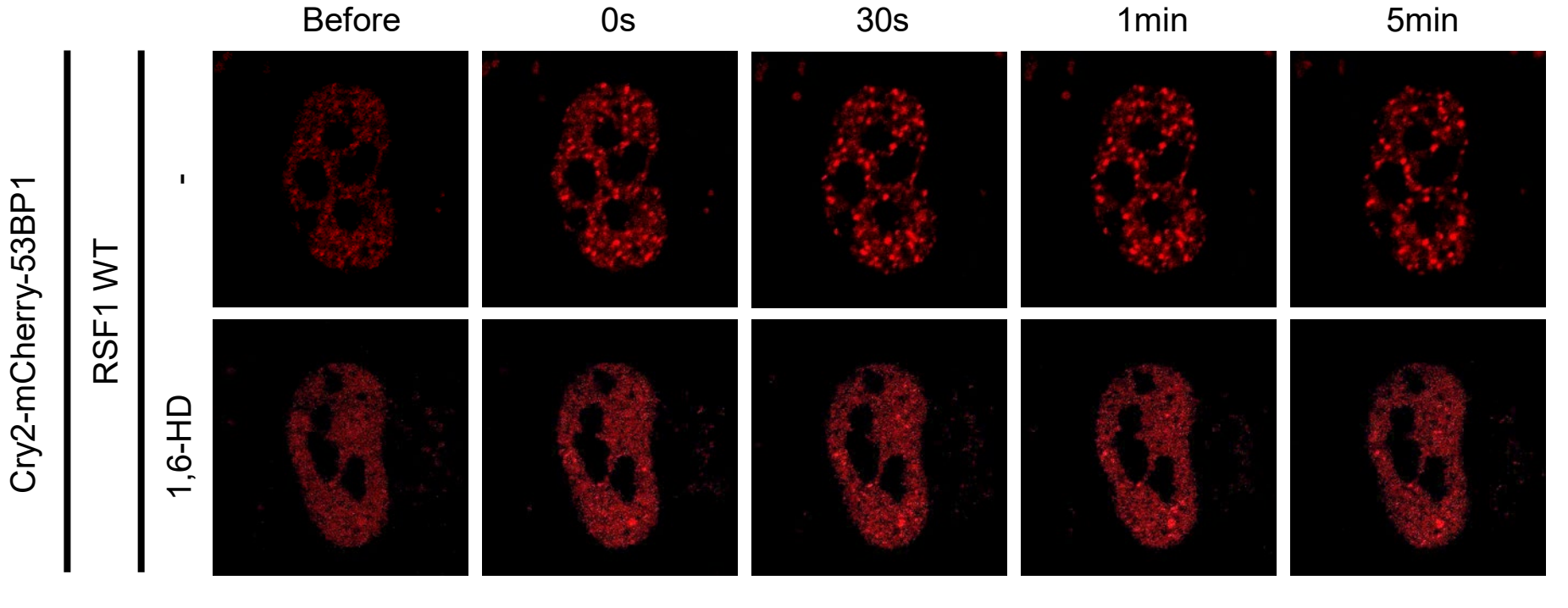

Figure S1E

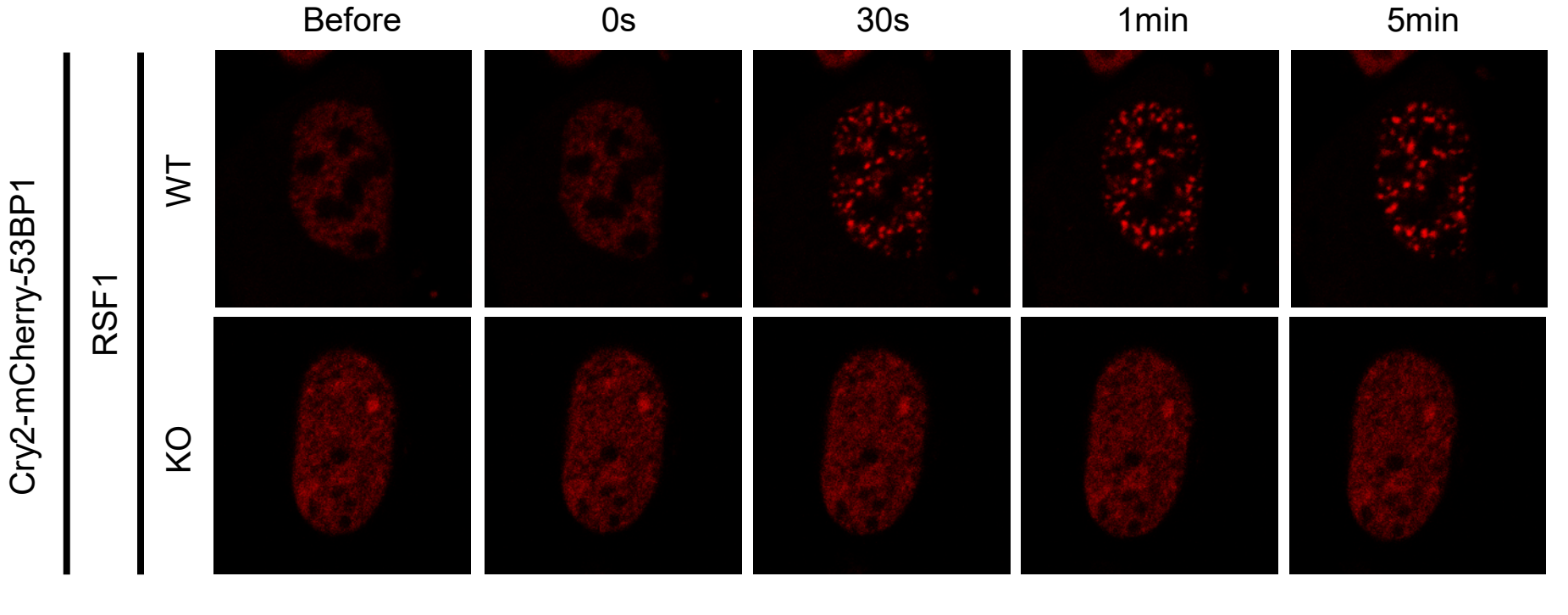

Figure S1F

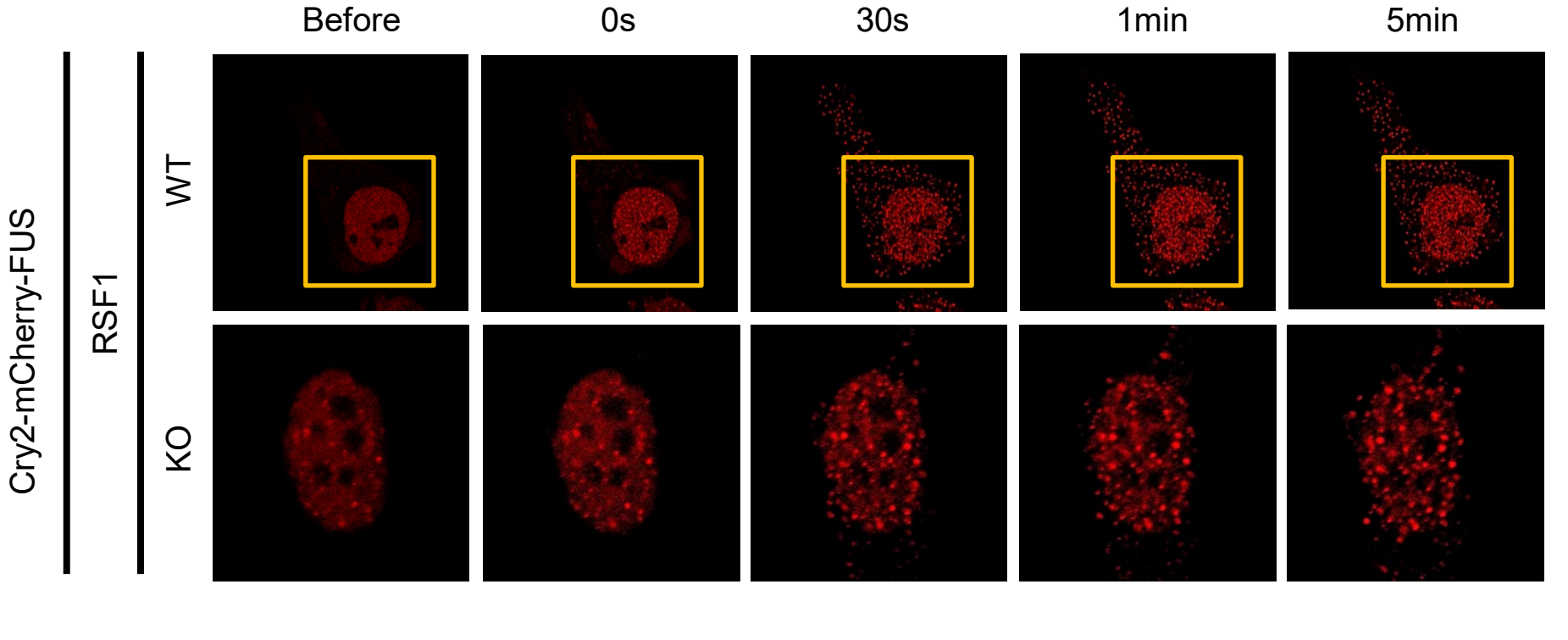

Figure S1G

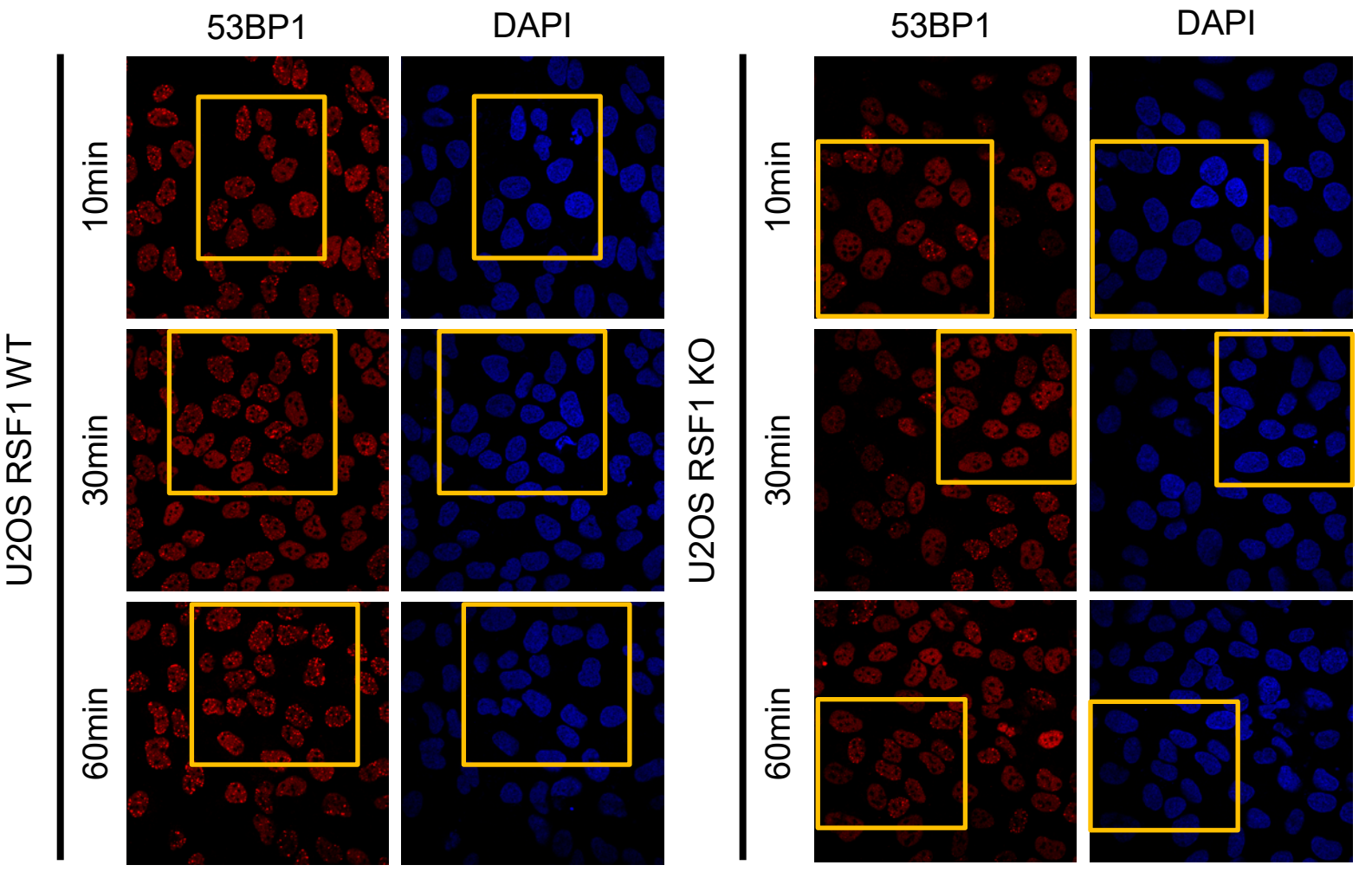

Figure S2A

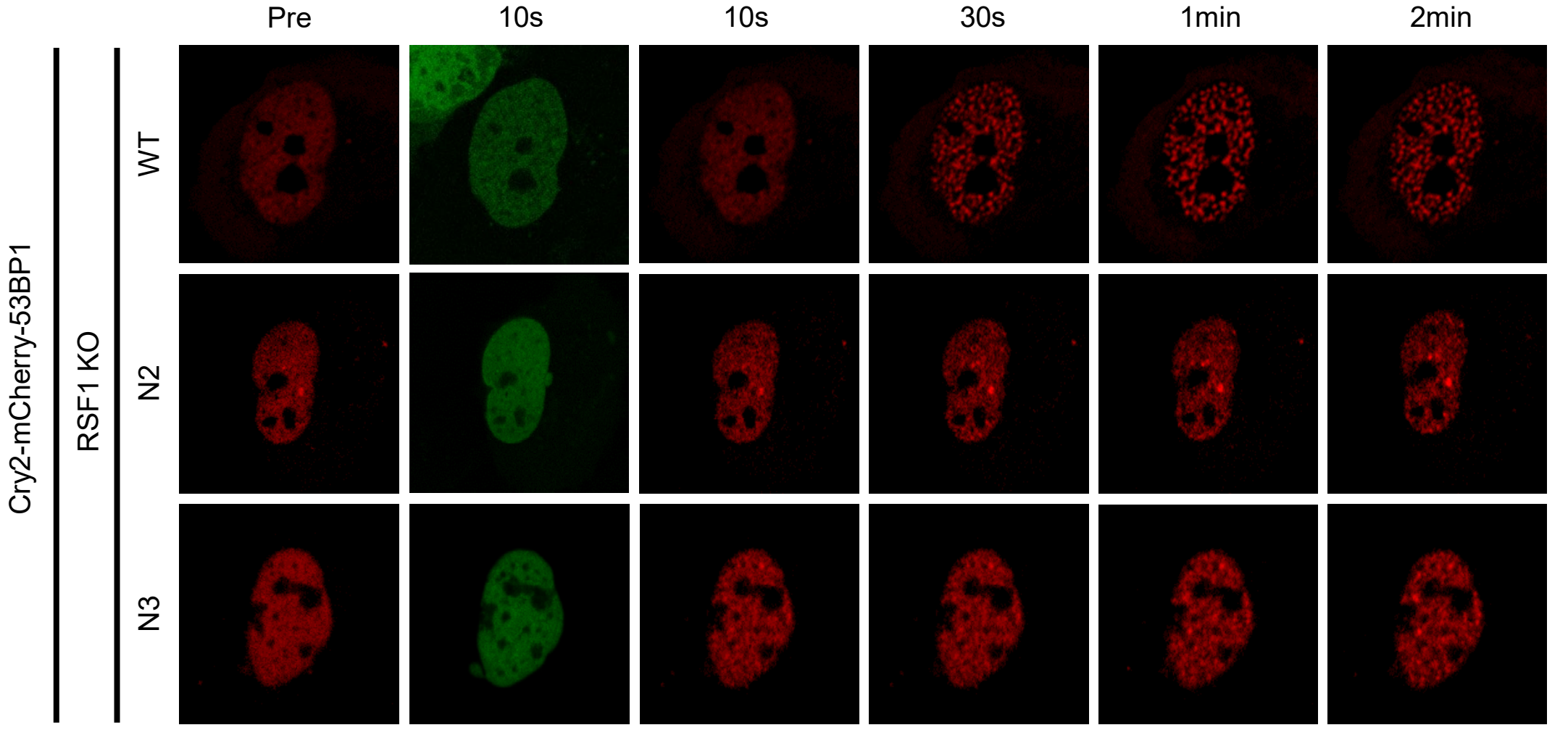

Figure S2B

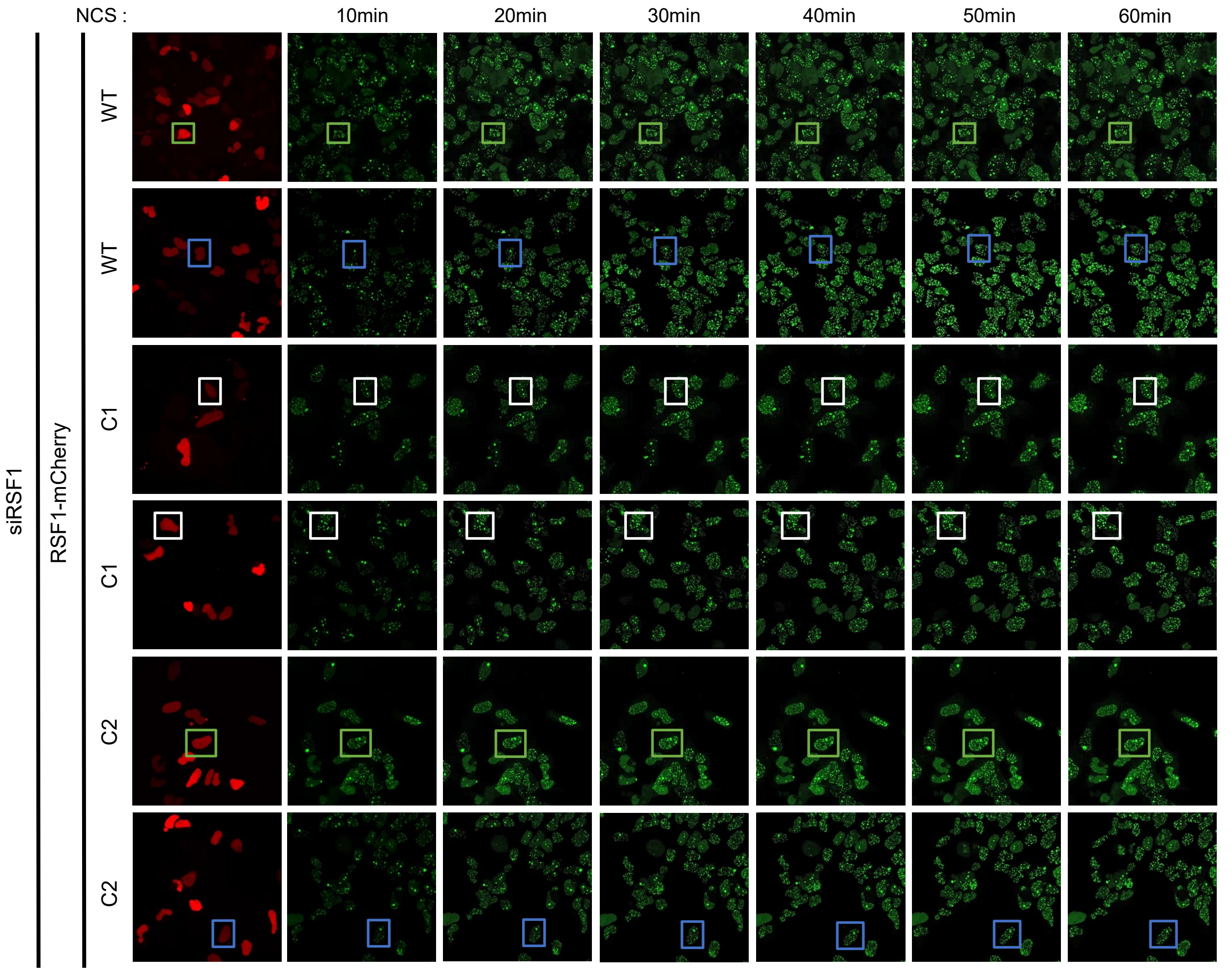

Figure S3C

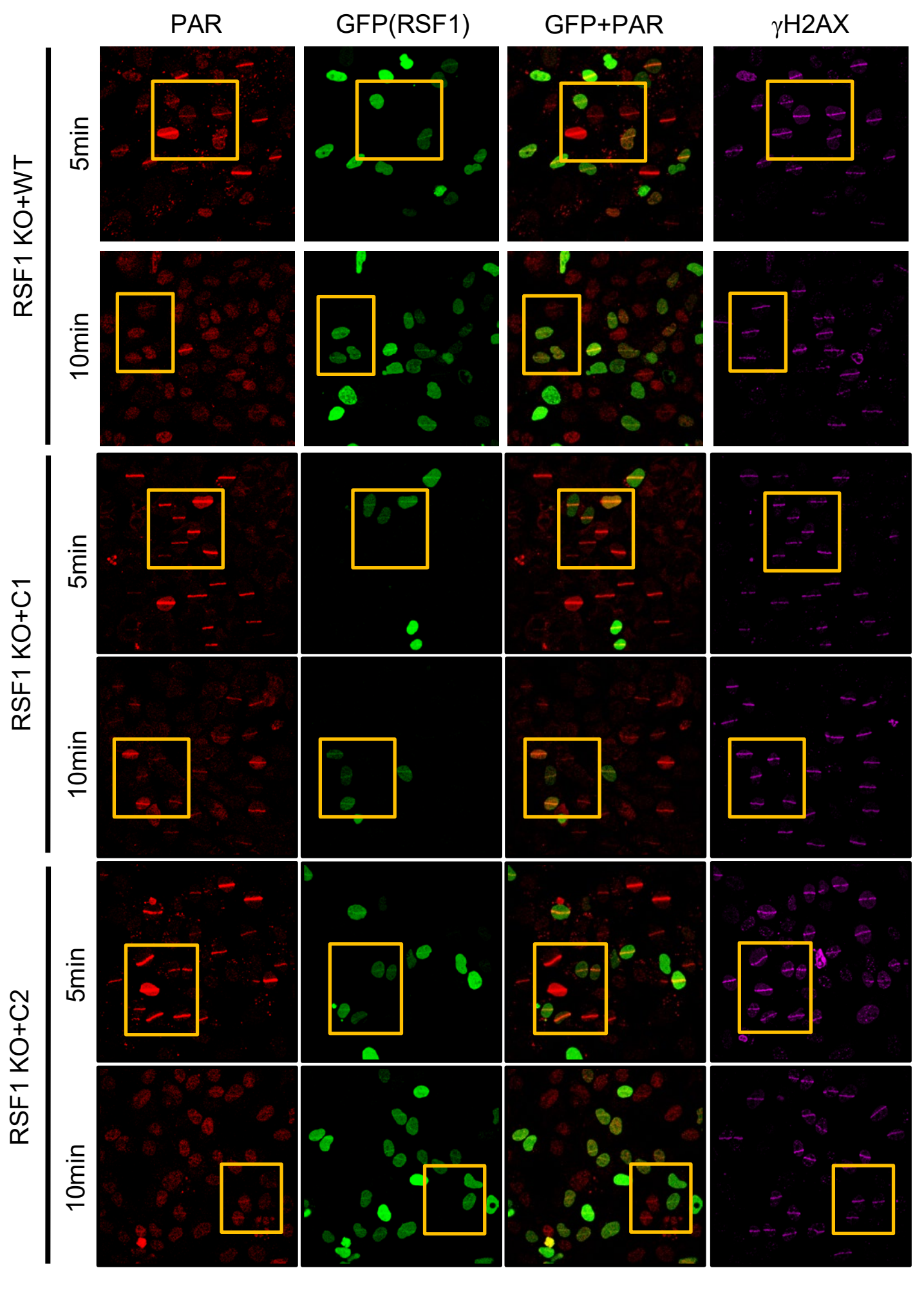

Figure S4A

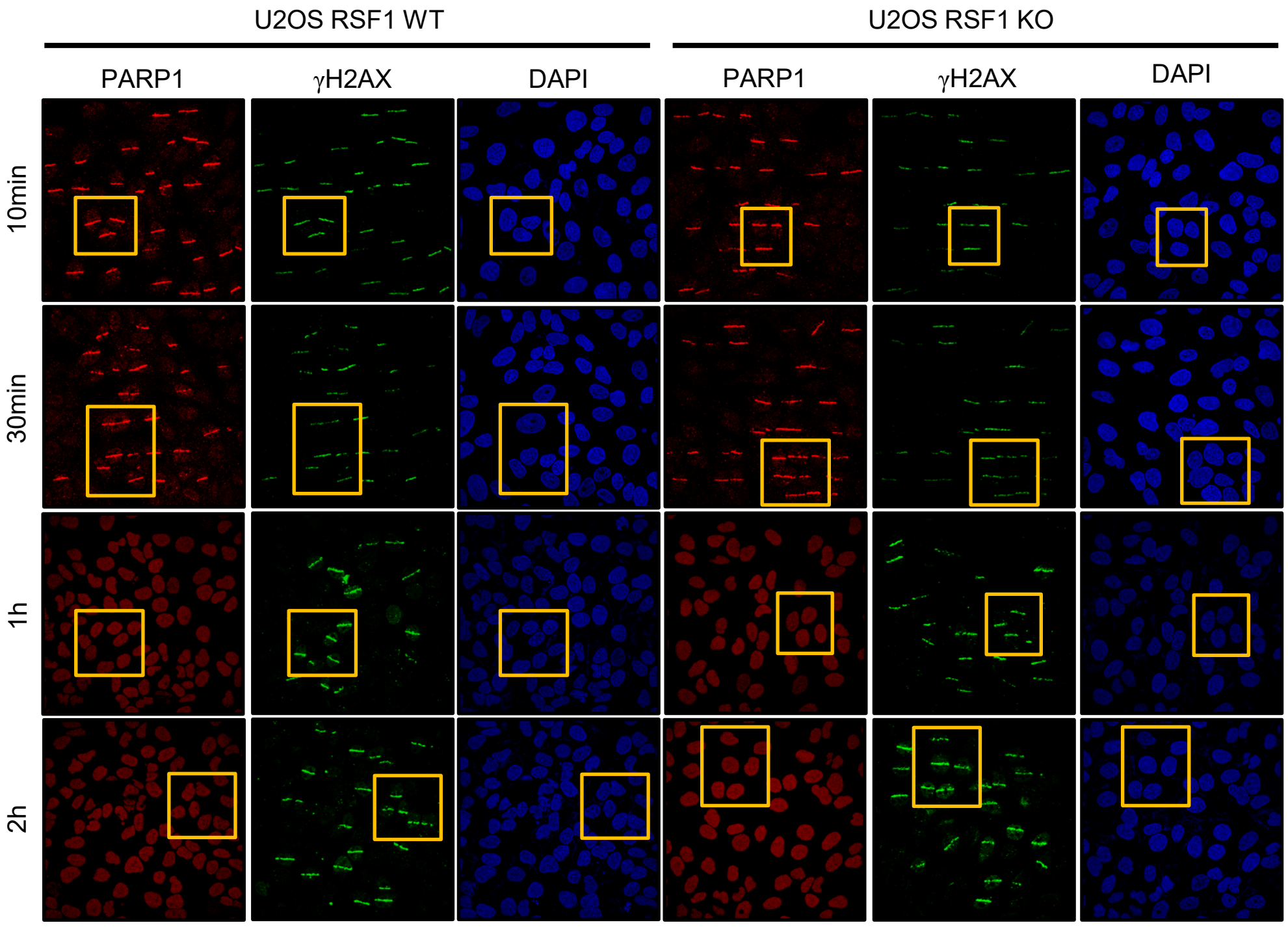

Figure S4B

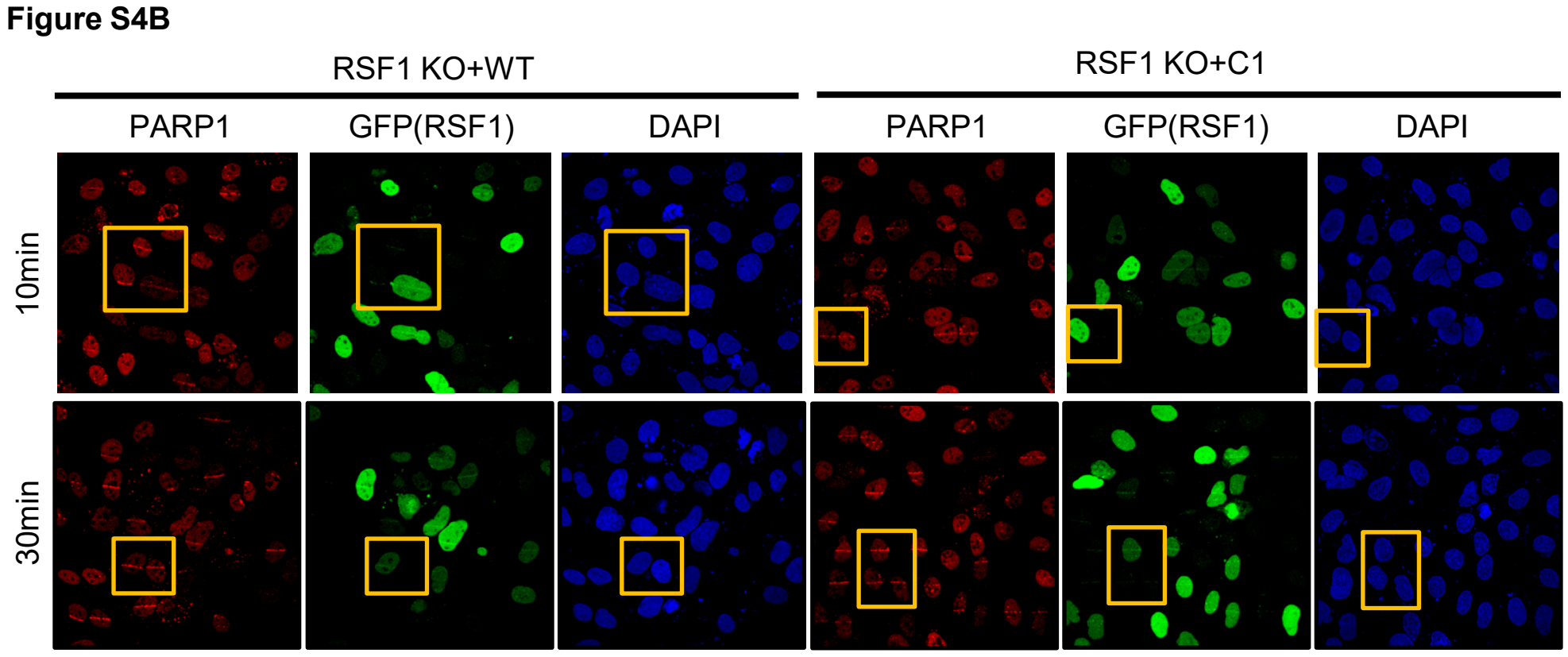

Figure S4C

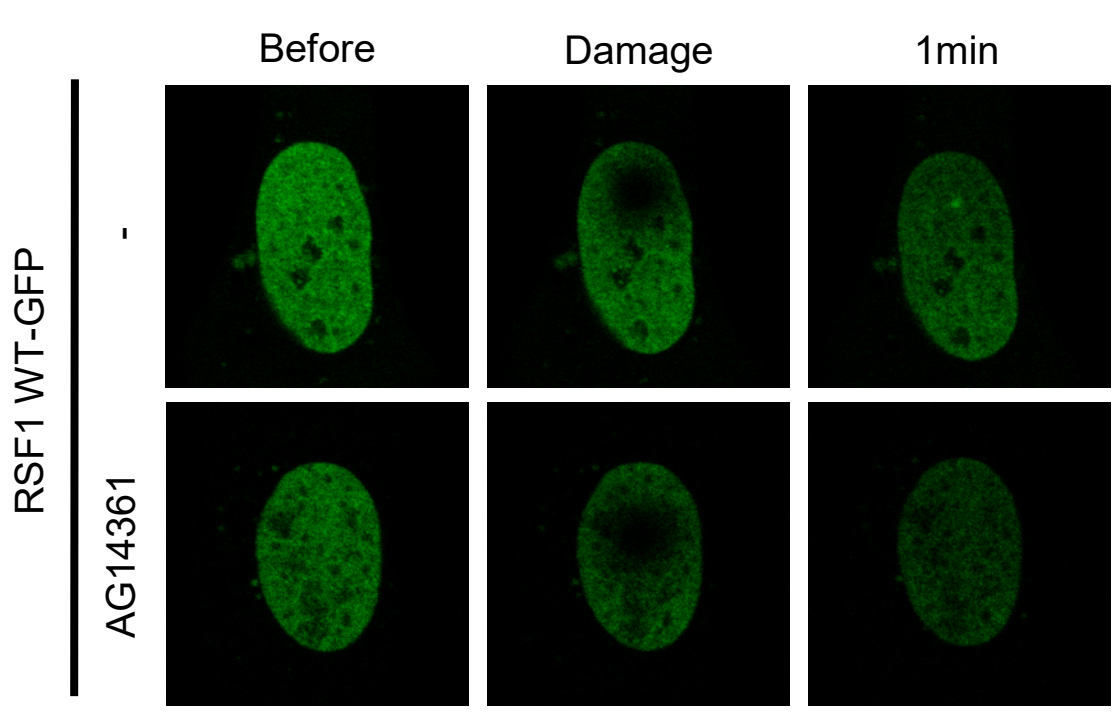

Figure S4D

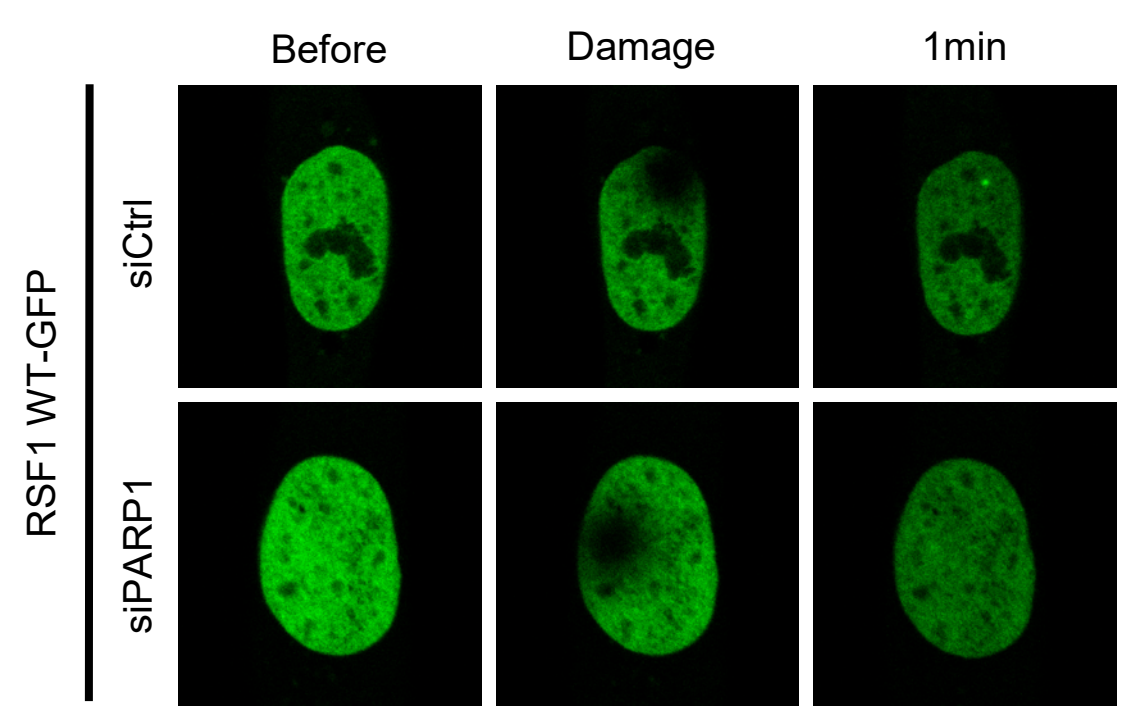

Figure S4E

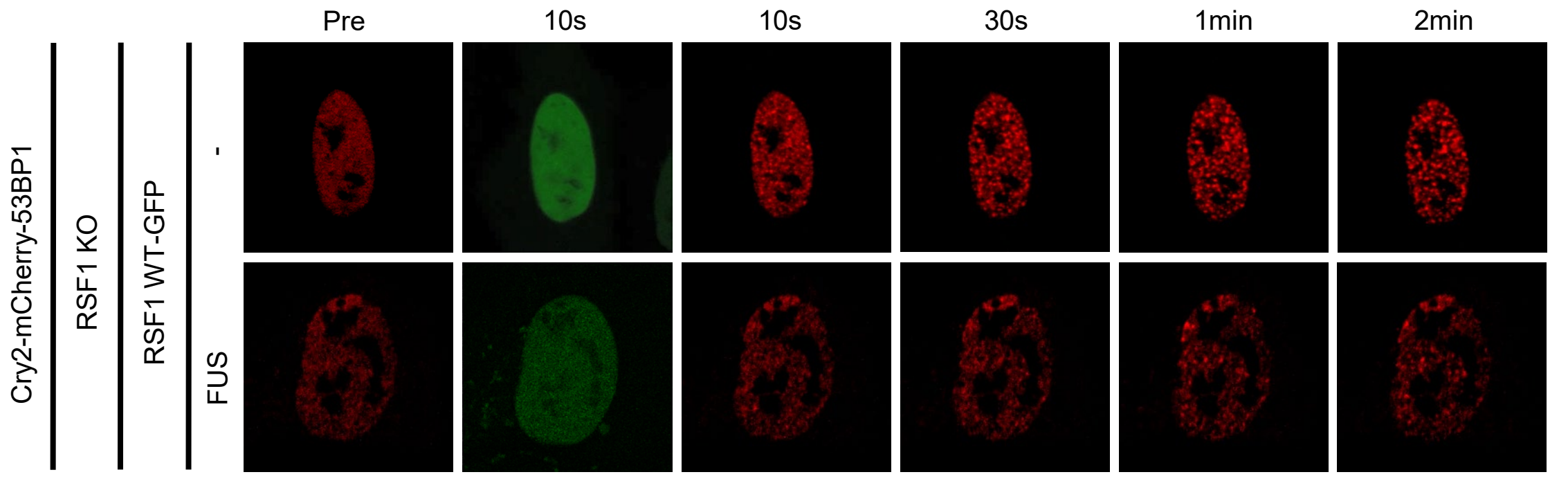

Figure S5D

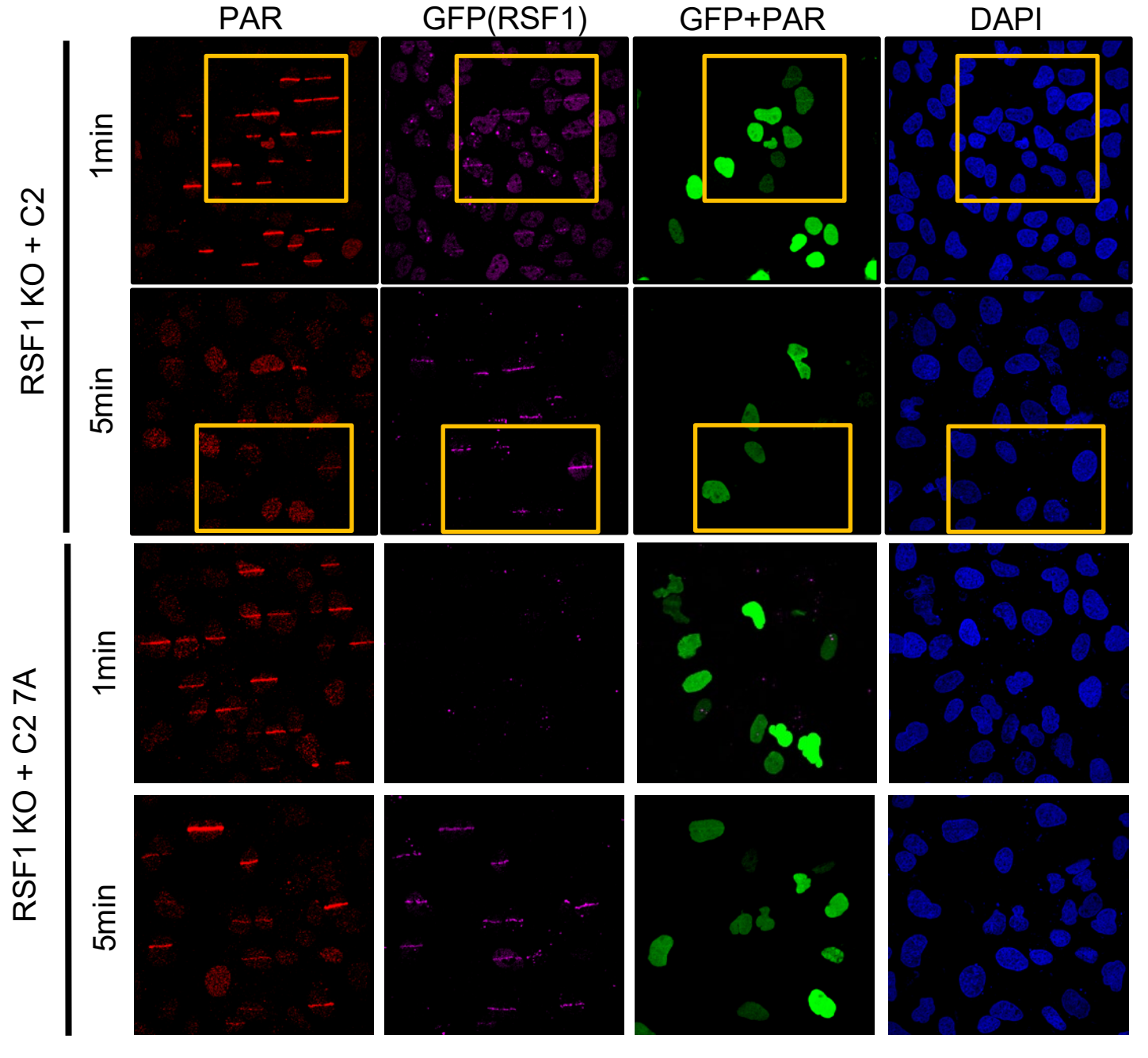

Supplement: Supplementary file 2 [file ADVS-13-e77271-s004.pdf]
