# Supplementary material for: RSF1‐Dependent PAR Turnover Promotes 53BP1 Liquid Condensate Formation at DNA Damage Sites
Source: Adv Sci (Weinh). 2026 Aug 24;13(63):e77271. doi: 10.1002/advs.77271 (PMC13502265; doi:10.1002/advs.77271)
Supplement: Supplementary file 3 [file ADVS-13-e77271-s001.docx]

Supporting Information

RSF1-dependent PAR turnover promotes 53BP1 liquid condensate formation at DNA damage sites

*Yungyeong Heo, Yonghyeon Kim,* *Nangyeong Hwang, Gwanho Sung, Sujin Jo, Yeonhu Lee, Ho-Chul Kang, Jae-Ho Lee, Yong Won Choi^*^, Hyeseong Cho^*^ and Sunwoo Min^*^*

**Figure S1.** (A) Structural prediction of RSF1 and analysis of light-induced condensate formation using the OptoDroplet system. Structural model of RSF1 from AlphaFold. High-confidence helical regions (pLDDT > 70) are shown in color, and predicted intrinsically disordered regions appear as extended loops. (B) Schematic of OptoDroplet constructs (RSF1 WT or C2 fused to mCherry–Cry2). (C) Representative blue-light–induced (488 nm) condensate formation in U2OS cells. RSF1 WT formed visible condensates, whereas C2 did not. Scale bar, 10 μm. (D) U2OS RSF1 WT cells expressing Cry2-mCherry-53BP1 W1495A were treated with 2% 1,6-hexanediol (HD) for 30 min before light activation. HD abolished optoDroplet formation, confirming the liquid-like properties of 53BP1 condensates. Quantification from three independent biological experiments (n = 3) is presented as mean ± SEM (right panel). Statistical significance was determined using an unpaired two-tailed Student's t-test. P-values were as follows: ***P=<0.001. Scale bar, 10 μm. (E) Light-induced optoDroplet formation of Cry2-mCherry-53BP1 WT. Cells were imaged at 15-s intervals. Representative images before and 2 min after blue-light (488 nm) activation are shown. Quantification of 53BP1 optoDroplet numbers per cell from three independent biological experiments (n = 3) is presented as mean ± SEM (right panel). Statistical significance was determined using an unpaired two-tailed Student's t-test. P-values were as follows: ***P=<0.001. Scale bar, 10 μm. (F) Representative Cry2-mCherry-FUS optoDroplet images in RSF1 WT and KO cells. Quantification of FUS optoDroplet numbers per cell from three independent biological experiments (n = 3) is presented as mean ± SEM (right panel). Statistical significance was determined using an unpaired two-tailed Student's t-test. P-values were as follows: ns=>0.999. Scale bar, 10 μm. (G) Immunoblot analysis of parental and YFP-53BP1 knock-in (KI) cells using the indicated antibodies. Comparable expression of YFP-53BP1 in the KI cells and endogenous 53BP1 in parental cells was confirmed. β-Actin was used as a loading control. (H) Quantification of the number of 53BP1 foci per nucleus in YFP-53BP1 KI cells from the experiment shown in Figure 1E. Data are presented as mean ± SEM from two independent biological experiments (n = 2). Statistical significance was determined using an unpaired two-tailed Student's t-test. P-values were as follows: *P=0.0186, ns=>0.8646,0.4734. (I) Representative immunofluorescence images of endogenous 53BP1 (red) in U2OS RSF1 WT and KO cells following Neocarzinostatin (NCS, 200 ng/mL) treatment for the indicated times to induce DNA damage. Nuclei were counterstained with DAPI (blue). Quantification of 53BP1 foci area from three independent biological experiments (n = 3) is presented as mean ± SEM (right panel). Statistical significance was determined using one-way ANOVA followed by Tukey's multiple-comparison test. P-values were as follows: ***P=<0.001.

**Figure S2.** (A) Light-induced optoDroplet formation of Cry2-mCherry-53BP1 W1495A in RSF1 WT, RSF1 KO, and RSF1 KO cells reconstituted with RSF1 WT-GFP, NLS-RSF1 N2-GFP, or NLS-RSF1 N3-GFP. Representative images before and 2 min after 488 nm light activation are shown. Droplet numbers were quantified based on red puncta detected at 2 min after light activation (right panel). Quantification of 53BP1 optoDroplet numbers in individual cells from three independent biological experiments (n = 3) is presented as mean ± SEM (right panel). Statistical significance was determined using one-way ANOVA followed by Tukey's multiple-comparison test. P-values were as follows: ***P=<0.001. Scale bar, 10 μm. (B) Fusion and fission dynamics of 53BP1 condensates were monitored in YFP-53BP1 KI cells transfected with siRSF1 and rescued with the indicated RSF1-GFP constructs (WT, C1, or C2) following NCS treatment. Live-cell images were acquired every 5 min for 1 h, and representative frames at 10-min intervals are shown. Examples of 53BP1 fusion events (green arrowheads) and fission events (blue arrowheads) are indicated. Insets show magnified views of the indicated regions. Representative cells expressing the indicated RSF1-GFP constructs are shown on the left. Quantification of fusion, fission, and no-event frequencies from two independent biological experiments (n = 2) is presented as mean ± SEM (right panel). A total of n = 22 WT-rescued cells, n = 12 C1-rescued cells, n = 24 C2-rescued cells from two independent experiments were analyzed. Cells showing at least one fusion event accounted for 8/22 cells (36.3%) in WT-rescued cells, 1/12 cells (8.3%) in C1 rescued cells, and 9/24 cells (37.5%) in C2-rescued cells, whereas cells showing at least one fission event accounted for 11/22 cells (50%) in WT-rescued cells, 0/12 cells (0%) in C1 rescued cells, and 10/24 cells (41.7%) in C2-rescued cells, respectively. Statistical significance was determined using an unpaired two-tailed Student's t-test. P-values were as follows: **P=0.0079, ns=0.3672. Scale bar, 10 μm.

**Figure S3.** (A) Recombinant GST-RSF1 constructs were spotted on nitrocellulose membranes and incubated in ribosylation buffer. PAR binding was detected by immunoblotting (upper panel). The expected molecular weights of GST-fused RSF1 constructs are approximately 245 kDa (WT), 160 kDa (C1), and 110 kDa (C2). Recombinant proteins in each reaction were examined by immunoblotting (middle panel) and Coomassie blue staining (lower panel). (B) U2OS cells were transfected with GFP-RSF1 WT, C1, or C2. Cells were treated with H₂O₂ (1 mM) for 2 h to induce DNA damage and subjected to immunoprecipitation using an anti-GFP antibody, followed by immunoblotting with the indicated antibodies. Red asterisks indicate the positions of GFP-RSF1 WT, C1, and C2 proteins. (C) U2OS RSF1 KO cells expressing GFP-RSF1 WT, C1, or C2 were subjected to laser microirradiation and fixed at the indicated time points. Cells expressing RSF1 WT or mutants are indicated (yellow, rescued cells; white, KO cells). Quantification of PAR fluorescence intensity at laser tracks, normalized to background levels, from three independent biological experiments (n = 3) is presented as mean ± SEM (right panel). Statistical significance was determined using one-way ANOVA followed by Tukey's multiple-comparison test. P-values were as follows: ***P=0.009, *P=0.0350, ns=0.4501, 0.7547. Scale bar, 10 μm.

**Figure S4.** (A) Immunostaining of PARP1 and γH2AX in U2OS RSF1 WT and KO cells after laser microirradiation. Cells were fixed at the indicated time points to examine PARP1 recruitment to DNA damage sites. Quantification of PARP1 fluorescence intensity at laser tracks, normalized to background fluorescence intensity, from three independent biological experiments (n = 3) is presented as mean ± SEM (right panel). Statistical significance was determined using an unpaired two-tailed Student's t-test. P-values were as follows: ns=0.7202, 0.6926. Scale bar, 10 μm. Scale bar, 10 μm. (B) Analysis of PARP1 accumulation kinetics in RSF1 KO cells reconstituted with GFP-RSF1 WT or GFP-RSF1 C1. Cells expressing RSF1 WT or C1 are marked by dashed circles (yellow, rescued cells; white, KO cells). Quantification of PARP1 fluorescence intensity at laser tracks, normalized to background fluorescence intensity, from three independent biological experiments (n = 3) is presented as mean ± SEM (right panel). Statistical significance was determined using an unpaired two-tailed Student's t-test. P-values were as follows: ns=0.7809, 0.7563. Scale bar, 10 μm. (C) Representative live-cell images showing recruitment of RSF1 WT-GFP to laser microirradiation-induced DNA damage sites in the absence or presence of the PARP inhibitor AG14361. Images were acquired before and 1 min after DNA damage induction. RSF1 WT-GFP enrichment at laser-induced DNA damage sites was ultimately observed in 15/15 cells (100%) in both groups. However, AG14361 treatment significantly delayed the recruitment kinetics of RSF1 WT-GFP compared with the control group. The fraction of cells showing RSF1 WT-GFP accumulation at DNA damage sites was quantified over time (lower panel) and is presented as mean ± SEM from three independent biological experiments (n = 3). Statistical significance was determined using an unpaired two-tailed Student's t-test. P-values were as follows: *P=0.0411,0.0319, **P=0.00343. Scale bar, 10 μm. (D) Representative live-cell images showing RSF1 WT-GFP accumulation at DNA damage sites in cells transfected with control siRNA or PARP1 siRNA. Images were acquired at the indicated time points following laser microirradiation. A total of n = 15 siCtrl cells and n = 15 siPARP1 cells from three independent biological experiments were analyzed. RSF1 WT-GFP enrichment at laser-induced DNA damage sites was ultimately observed in 15/15 cells (100%) in both groups; however, PARP1 depletion delayed the recruitment kinetics compared with siCtrl cells. The fraction of cells showing RSF1 WT-GFP accumulation at DNA damage sites was quantified over time (lower panel) and is presented as mean ± SEM from three independent biological experiments (n = 3). Statistical significance was determined using an unpaired two-tailed Student's t-test. P-values were as follows: *P=0.0263, **P=0.0061, ns=0.0889. Scale bar, 10 μm. (E) FUS overexpression disrupts RSF1-mediated 53BP1 condensate formation. RSF1 KO U2OS cells were co-transfected with Cry2-mCherry-53BP1 W1495A together with either control vector or a FUS expression plasmid and illuminated with 488 nm light to induce 53BP1 optoDroplet formation. Quantification of 53BP1 optoDroplet numbers per cell from three independent biological experiments (n = 3) is presented as mean ± SEM (right panel). Statistical significance was determined using an unpaired two-tailed Student's t-test. P-values were as follows: ***P=<0.001. Scale bar, 10 μm.

**Figure S5.** (A) Predicted structures of PARG in complex with full-length RSF1, C1, and C2 generated using AlphaFold-Multimer. The top-ranked models were visualized using Mol* Viewer. PARG is shown in green and RSF1 constructs are shown in orange. (B) Predicted PARG-contacting regions in full-length RSF1, C1, and C2. Representative RSF1 residues located within 4.5 Å of PARG are highlighted (green), and arrows indicate putative PARG-interacting regions identified from structural modeling. (C) Multiple sequence alignment of RSF1 SYRFDEFDEAI region among hyman, mouse, and rat proteins. Conserved residues are highlihted in red boxes. (D) U2OS RSF1 KO cells expressing GFP-RSF1 C2 or C2 7A (DEFDEAI→AAAAAAA) were subjected to laser microirradiation and fixed at the indicated time points. Cells expressing RSF1 constructs are indicated (yellow, rescued cells; white, KO cells). Quantification of PAR and 53BP1 fluorescence intensity at laser tracks, normalized to background levels, from three independent biological experiments (n = 3) is presented as mean ± SEM (right panel). Statistical significance was determined using one-way ANOVA followed by Tukey's multiple-comparison test. P-values were as follows: PAR: **P=0.010, ns=0.380, 53BP1: **P=0.0020, 0.004. Scale bar, 10 μm.

**Figure S6.** (A) U2OS cells transfected with the indicated RSF1 constructs were treated with increasing concentrations of etoposide (0, 0.1, 0.5, and 1 μM) or phleomycin (0, 0.5, 1, and 2 μg/mL) and subjected to clonogenic survival assays. Representative colonies (upper panels) and quantification of colony survival from three independent biological experiments (n = 3) are presented as mean ± SEM (lower panels). Statistical significance was determined using one-way ANOVA followed by Tukey's multiple-comparison test. P-values were as follows: ***P=<0.001, *P=0.0219, ns=0.1849. (B) U2OS DR-GFP and EJ5-GFP cells were co-transfected with siRSF1 and the indicated RSF1 constructs (WT, C1, or C2). The following day, cells were transfected with an I-SceI expression plasmid to induce DNA double-strand breaks. GFP-positive cells were quantified by flow cytometry 72 h later to assess homologous recombination (DR-GFP) and non-homologous end joining (EJ5-GFP) repair efficiency. Expression of the indicated RSF1 rescue constructs (WT, C1, and C2) in siRSF1-treated cells was confirmed by immunoblotting (right panel). Data are presented as mean ± SEM from three independent biological experiments (n = 3). Statistical significance was determined using one-way ANOVA followed by Tukey's multiple-comparison test. P-values were as follows: DR: ***P=<0.001,**P=0.020, ns=0.085, EJ: ***P=<0.001, **P=0.001.

**Figure S7.** Schematic model of RSF1-mediated sequential condensate formation in response to DNA damage.

**Table S1. HADDOCK RSF1 C2 or mutants with PARG docking results.**

**Table S2.** Primers for plasmid construction.

| Gene symbol | Primer sequences (5’ → 3’) |
| --- | --- |
| Cry2_mCh_lin | FW: AGCGGCCGCGACTCTAG  RV: CTTGTACAGCTCGTCCATGCC |
| RSF1_WT_Cry2 | FW: CGGCGGCATGGACGAGCTGTACAAGATGGCGACGGCGGCGGCAGCGGCGGCGGGATG  RV: TATGATCTAGAGTCGCGGCCGCTTTATAACTGTTCACTGTTACAGACATAATCAACAAG |
| RSF1_D6_Cry2 | FW: CGGCGGCATGGACGAGCTGTACAAGATTCCTCCACAAGAGCCAGACTTTTCTGAAGATCAAG  RV: TATGATCTAGAGTCGCGGCCGCTTTATAACTGTTCACTGTTACAGACATAATCAACAAGG |
| RSF1_C2_ SYRF mutation | FW:TAGCTTCATCAATTGCTTCATCAAACTCATCAGCTGCGGCGGCTATACATTTCCTTGTTCTTGTTGACCTCCTTTCAAGCAAGTTTG  RV: CAAACTTGCTTGAAAGGAGGTCAACAAGAACAAGGAAATGTATAGCCGCCGCAGCTGATGAGTTTGATGAAGCAATTGATGAAGCTA |
| RSF1_C2_ DEFDEAI mutation | FW: CGGCTTCTTTGATGTCATCTTCAATAGCTTCATCAGCTGCTGCAGCAGCCGCAGCAAATCTGTAGCTTATACATTTCCTTGTTCTTGTT  RV: AACAAGAACAAGGAAATGTATAAGCTACAGATTTGCTGCGGCTGCTGCAGCAGCTGATGAAGCTATTGAAGATGACATCAAAGAAGCCG |
| RSF1_F | FW: GTTCTTTTTGCAGGATCCATGGCGACGGCGGC |
| RSF1_N2_R | RV: CCTTGAATTCGAATCGATGAGATGATTTTTCACTGCCAGACCC |
| RSF1_N3_R | RV: CCTTGAATTCGAATCGATGATACACCAAGCGTTCTTTTCTTCG |

**Table S3.** Sequences of siRNA.

| Gene symbol | siRNA sequences (5’ → 3’) |
| --- | --- |
| Negative control | AAUUCUCCGAACGUGUCACGU |
| RSF1 | GGAAAAUGUCAAACCCAUU |
| PARG | AGGCUAUGCUGAGACAUAU |
| PARP1 | GCUUCAAGUGUCAAGGAAATT |

**Table S4.** Reagent and resources used in this study.

| REAGENT or RESOURCE | Source | Catalog No. |
| --- | --- | --- |
| Antibodies | | |
| Mouse monoclonal anti-Poly (ADP-ribose) | Novus Biologicals | nb100-60654 |
| Mouse monoclonal anti-PARP1 | Bd bioscience | 556494 |
| Rabbit monoclonal anti-53BP1 | Cell signaling | 4937S |
| Rabbit monoclonal anti-53BP1 | Novus Biologicals | NB100-305 |
| Mouse monoclonal anti-Flag | Sigma | F3165 |
| Mouse monoclonal anti-FUS/TLS (4H11) | Santa Cruz | sc-47711 |
| Rabbit monoclonal anti-PARG | Cell signaling | 66564S |
| Mouse monoclonal anti-GAPDH | Santa Cruz | sc-25778 |
| Mouse monoclonal anti-γ-H2AX(Ser139) | Cell signaling | 2577S |
| Mouse monoclonal anti-γ-H2AX(Ser139) | Millipore | 05-636 |
| Rabbit monoclonal anti- γ-H2AX(Ser139) | Novus Biologicals | 100-384 |
| Mouse monoclonal anti-P53 | Santa Cruz | sc-126 |
| Rabbit monoclonal anti-P21 | Cell signaling | 2947S |
| Mouse monoclonal anti-GST(B-14) | Santa Cruz | sc-138 |
| Mouse monoclonal anti-GFP(B-2) | Santa Cruz | sc-9996 |
| Mouse monoclonal anti-GFP | Abcam | Ab290 |
| Rabbit monoclonal anti-RSF1 | Abcam | ab109002 |
| Anti-mouse IgG, HRP-linked Antibody | Invitrogen | 170-6516 |
| Anti-rabbit IgG, HRP-linked Antibody | Invitrogen | 170-6515 |
| Alexa Fluor 647-conjugated anti-Mouse | Thermo Fisher Scientific | 21236 |
| Alexa Fluor 488 goat anti-mouse IgG H&L | Invitrogen | A11059 |
| Alexa Fluor 546 goat anti-rabbit IgG H&L | Invitrogen | A11035 |
| Alexa Fluor 647 goat anti-rabbit IgG H&L | Invitrogen | A21245 |
| Alexa Flour Cyanine3-conjugate goat anti-mouse IgG H&L | Invitrogen | A10521 |
| Bacterial and virus stranins | | |
| One Shot™ MAX Efficiency™ DH5α-T1R Competent Cells | Invitrogen | 12297016 |
| One Shot™ TOP10 Chemically Competent E. coli | Invitrogen | C404010 |
| Chemicals, peptides and recombinant proteins | | |
| 5-bromo-2 deoxyuridine (BrdU) | Calbiochem | 203806 |
| Etoposide | From YONG WON CHOI |  |
| 1,6-Hexanediol | Sigma-Aldrich | 240117-50G |
| Hydrogen peroxide solution | Sigma-Aldrich | H1009-500ML |
| Olaparib, PARP inhibitor | Selleckchem | S1060 |
| AG14361, PARP inhibitor | Selleckchem | S2178 |
| Phleomysin | Sigma-Aldrich | P9564 |
| Neocarzinostatin from Streptomyces carzinostaticus | Sigma-Aldrich | N9162 |
| Nuclease, Micrococcal | Worthington | LS004797 |
| Benzonase nuclease | Millipore | 70664 |
| Formaldehyde solution | Sigma-Aldrich | F8775 |
| Pfu Turbo DNA polymerase | Agilent | 600250-52 |
| Experimental models: Cell lines | | |
| Human: U2OS | ATCC | HTB-96 |
| Human: U2OS RSF1 KO | From Hyeseong Cho | PMID: 26259146 |
| Human: HEK293T RSF1 KO | From Hyeseong Cho | PMID: 26259146 |
| Human: U2OS-DR-GFP | From Jeremy Stark | N/A |
| Human: U2OS-EJ-GFP | From Jeremy Stark | N/A |
| Recombinant DNA | | |
| Cry2-FUS-mCherry | From Matthias Altmeyer | PMID: 31267591 |
| Cry2-53BP1-WT-mCherry | From Matthias Altmeyer | PMID: 31267591 |
| Cry2-53BP1-W1495A-mCherry | From Matthias Altmeyer | PMID: 31267591 |
| Cry2-RSF1-WT-mCherry | This paper | N/A |
| Cry2-RSF1-C2-mCherry | This paper | N/A |
| RSF1-WT-GFP | From Sunwoo Min | PMID: 24351651 |
| RSF1-C1-GFP | From Sunwoo Min | PMID: 24351651 |
| RSF1-C2-GFP | From Sunwoo Min | PMID: 24351651 |
| RSF1-N2-GFP | This paper | N/A |
| RSF1-N3-GFP | This paper | N/A |
| RSF1-C2-SYRF mutant-GFP | This paper | N/A |
| RSF1-C2-DEFDEAI mutant-GFP | This paper | N/A |
| SFB empty vector | From Hongtae Kim | https://faculty.unist.ac.kr/khtlab/contact/ |
| SFB-RSF1-WT-V5 | From Hyeseong Cho | PMID: 26259146 |
| SFB-RSF1-N2-V5 | From Hyeseong Cho | PMID: 26259146 |
| SFB-RSF1-N3-V5 | From Hyeseong Cho | PMID: 26259146 |
| SFB-RSF1-C1-V5 | From Hyeseong Cho | PMID: 26259146 |
| SFB-RSF1-C2-V5 | From Hyeseong Cho | PMID: 26259146 |
| GST-RSF1-WT | From Ho-Soo Lee | PMID: 30242288 |
| GST-RSF1-C1 | From Ho-Soo Lee | PMID: 30242288 |
| GST-RSF1-C2 | From Ho-Soo Lee | PMID: 30242288 |
| pcDNA 3.1 | Addgene | V790-20 |

**Table S5.** Primers for RT-qPCR.

| Gene symbol | Primer sequences (5’ → 3’) |
| --- | --- |
| P21 | FW: TTGTACCCTTGTGCCTCGCT  RV: TTGGAGAAGATCAGCCGGC |
| PUMA | FW: GCTGCTGTAGATACCGGAATGAA  RV: AAAAAAATTAACCAAACATGTACAGAAAAT |
| GAPDH | FW: CTCTGCTCCTCCTGTTCGAC  RV: ACGACCAAATCCGTTGACTC |

**Table S6.** Primers for ChIP-qPCR.

| Gene symbol | primer sequences (5’ → 3’) |
| --- | --- |
| P21 + 182 | FW: CGTGTTCGCGGGTGTGT  RV: CATTCACCTGCCGCAGAAA |
| P21 -20 | FW: AGCTGCGCCAGCTGAGG  RV: CACAAGGAACTGACTTCGGCA |
| P21 -1391 | FW: CTGTCCTCCCCGAGGTCA  RV: ACATCTCAGGCTGCTCAGAGTCT |
| P21 -2283 | FW: AGCAGGCTGTGGCTCTGATT  RV: CAAAATAGCCACCAGCCTCTTCT |
