# Supplementary material for: RSF1‐Dependent PAR Turnover Promotes 53BP1 Liquid Condensate Formation at DNA Damage Sites
Source: Adv Sci (Weinh). 2026 Aug 24:e77271. Online ahead of print. doi: 10.1002/advs.77271 (PMC13502265; doi:10.1002/advs.77271)

Figure 1E

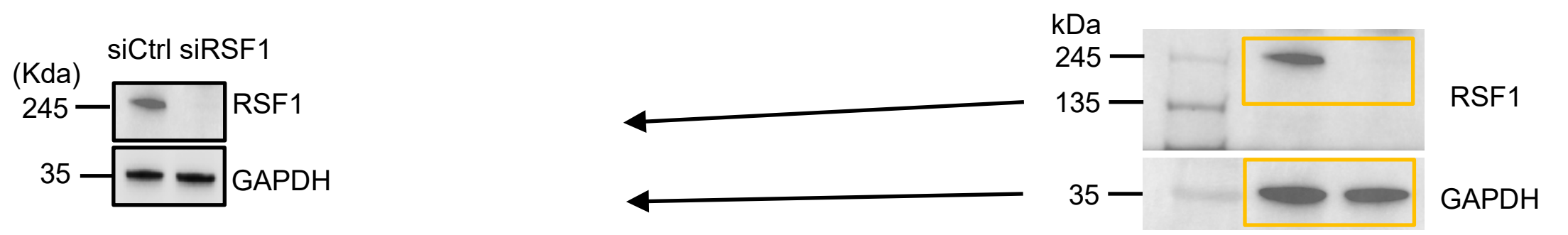

Figure 2D

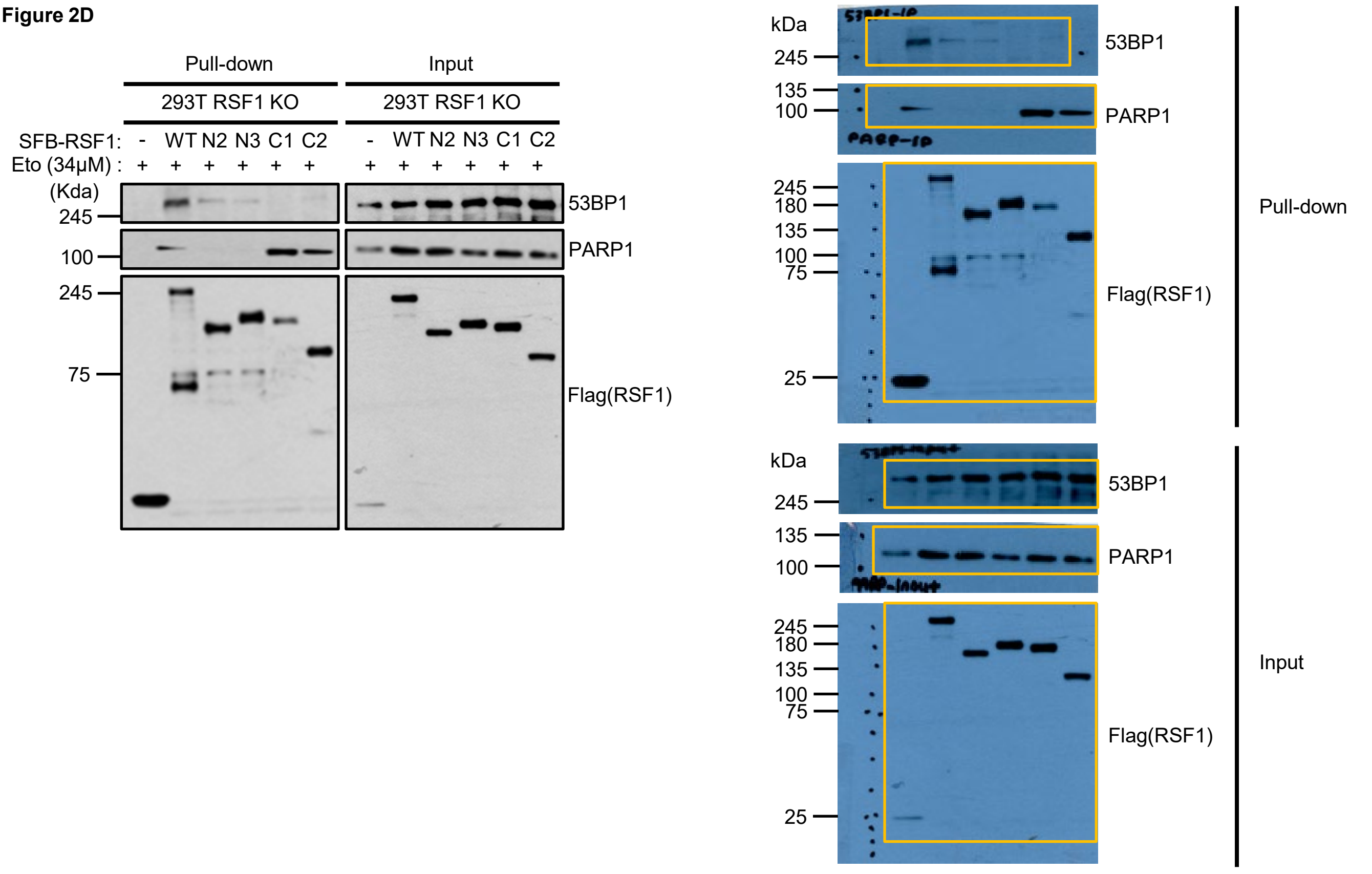

Figure 3A

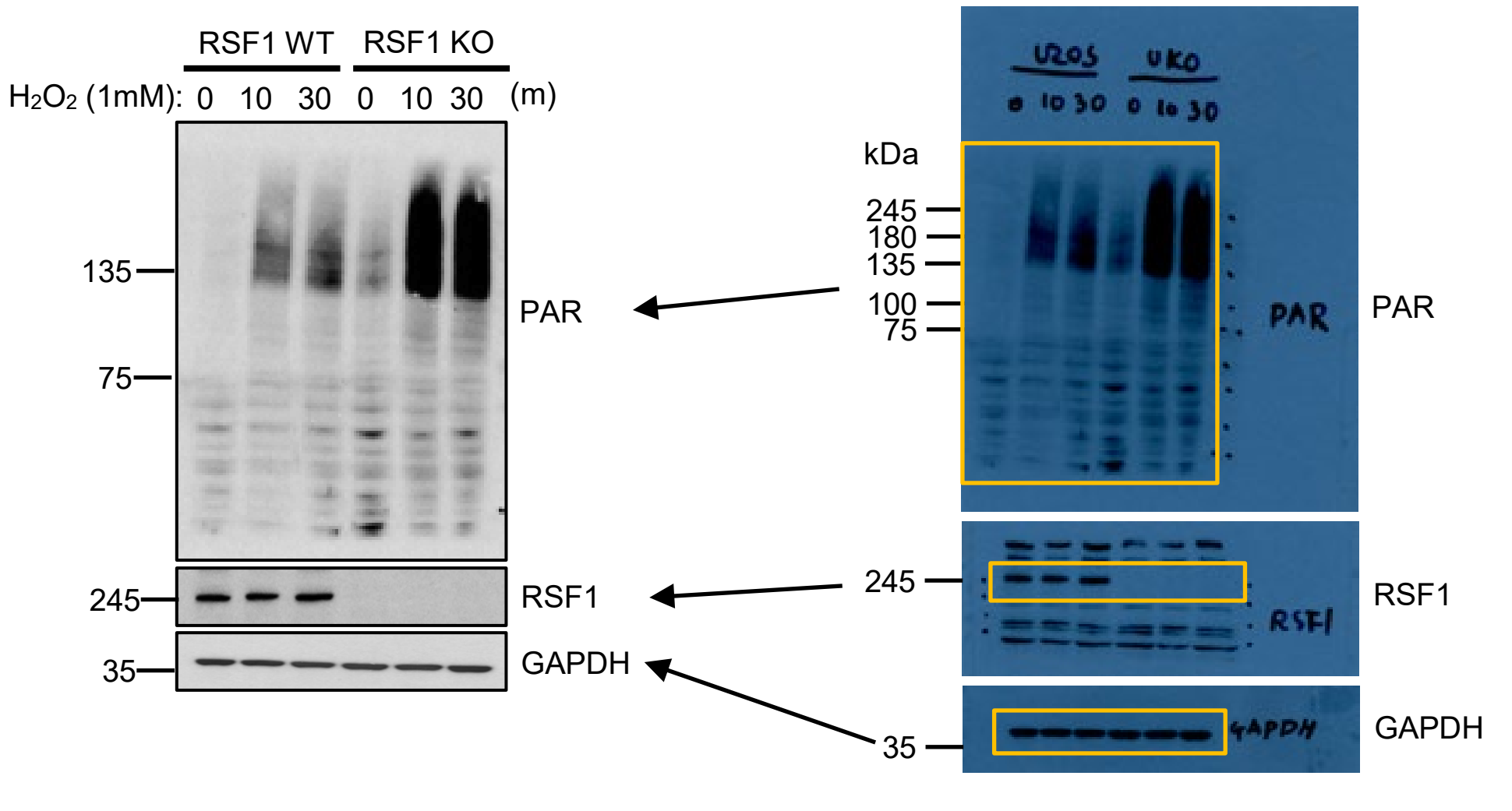

Figure 3B

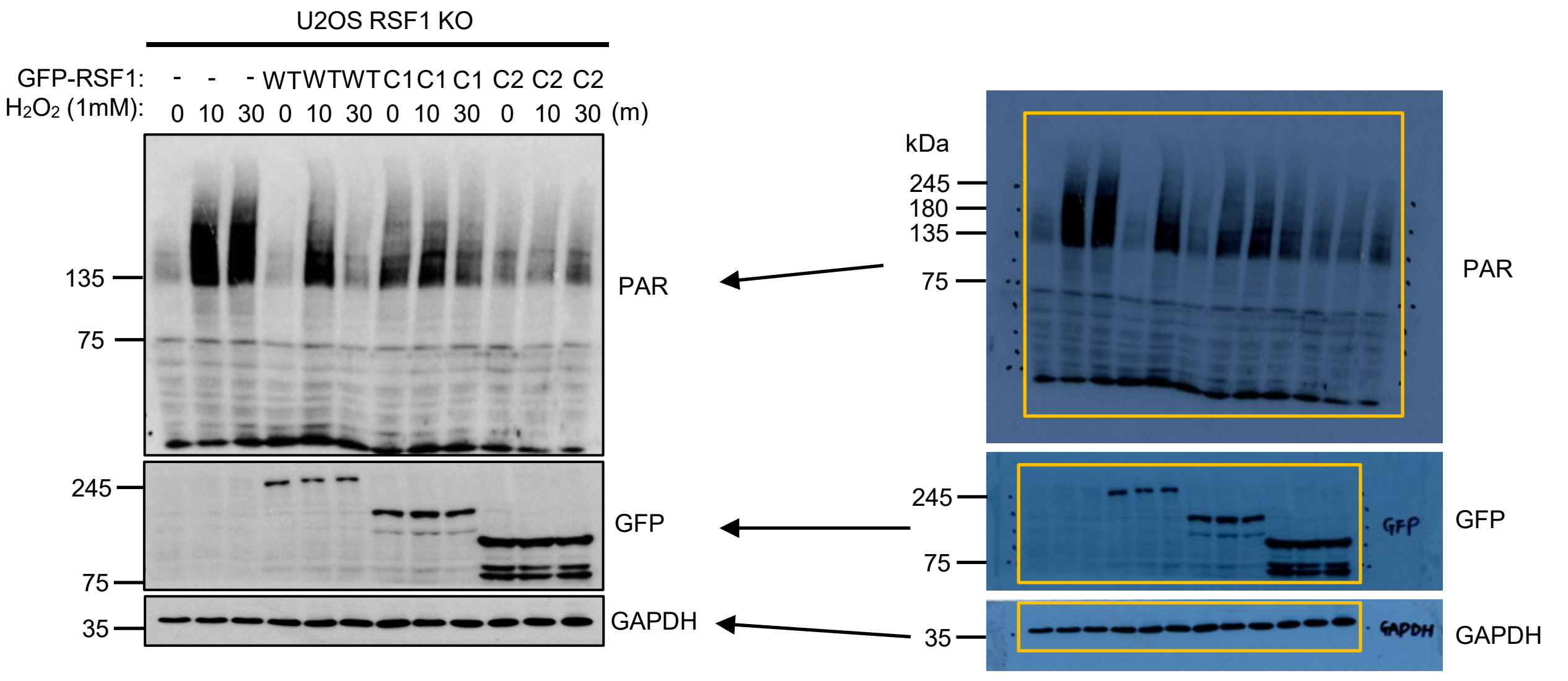

Figure 4C

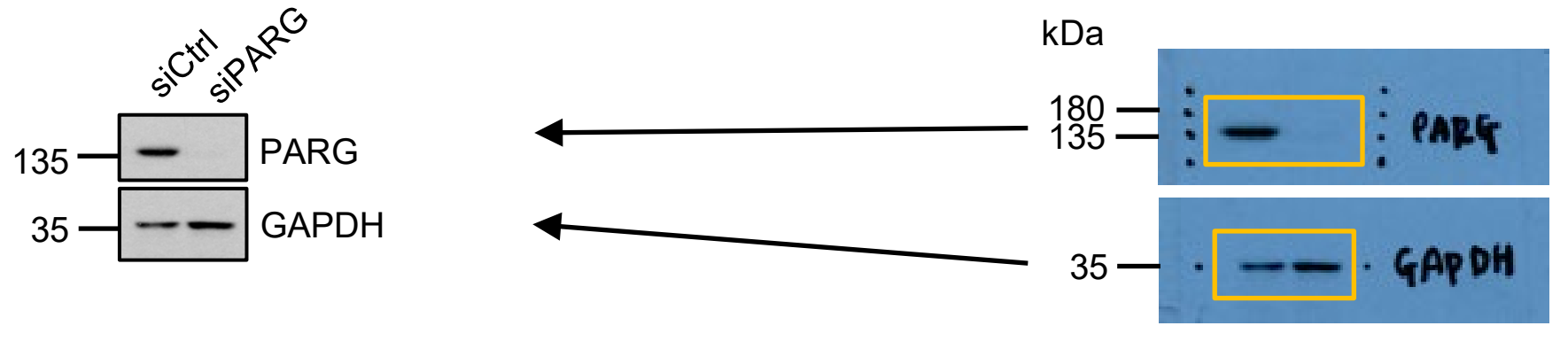

Figure 4F

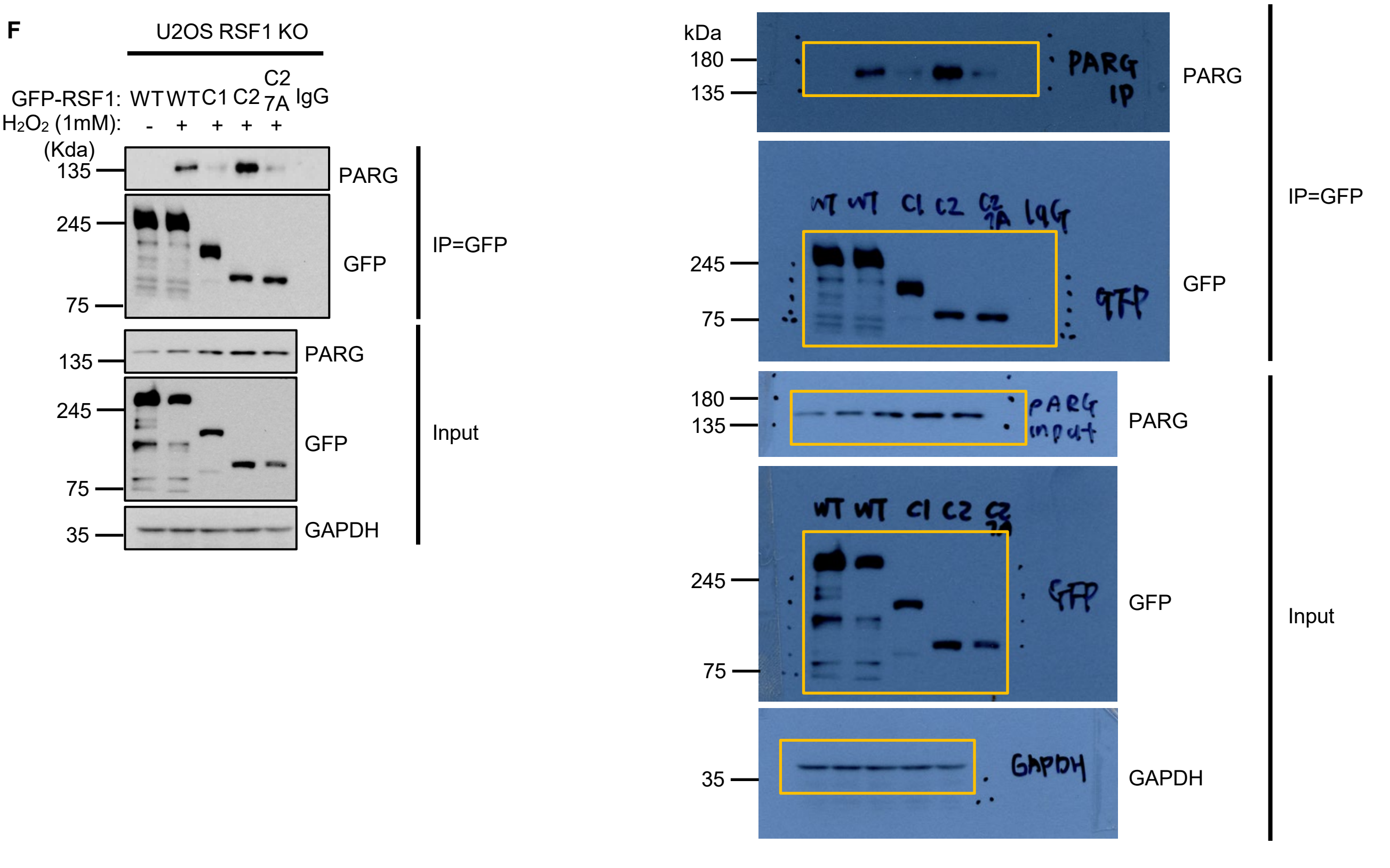

Figure 5A

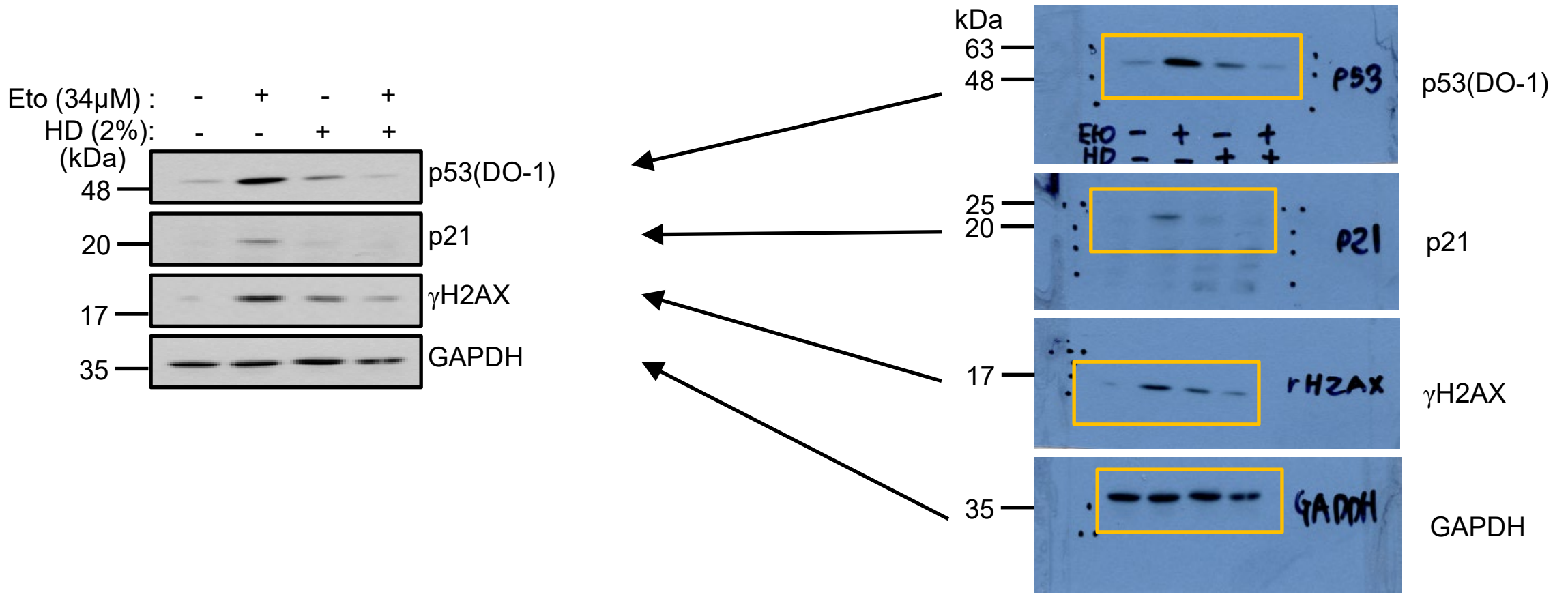

Figure 5D

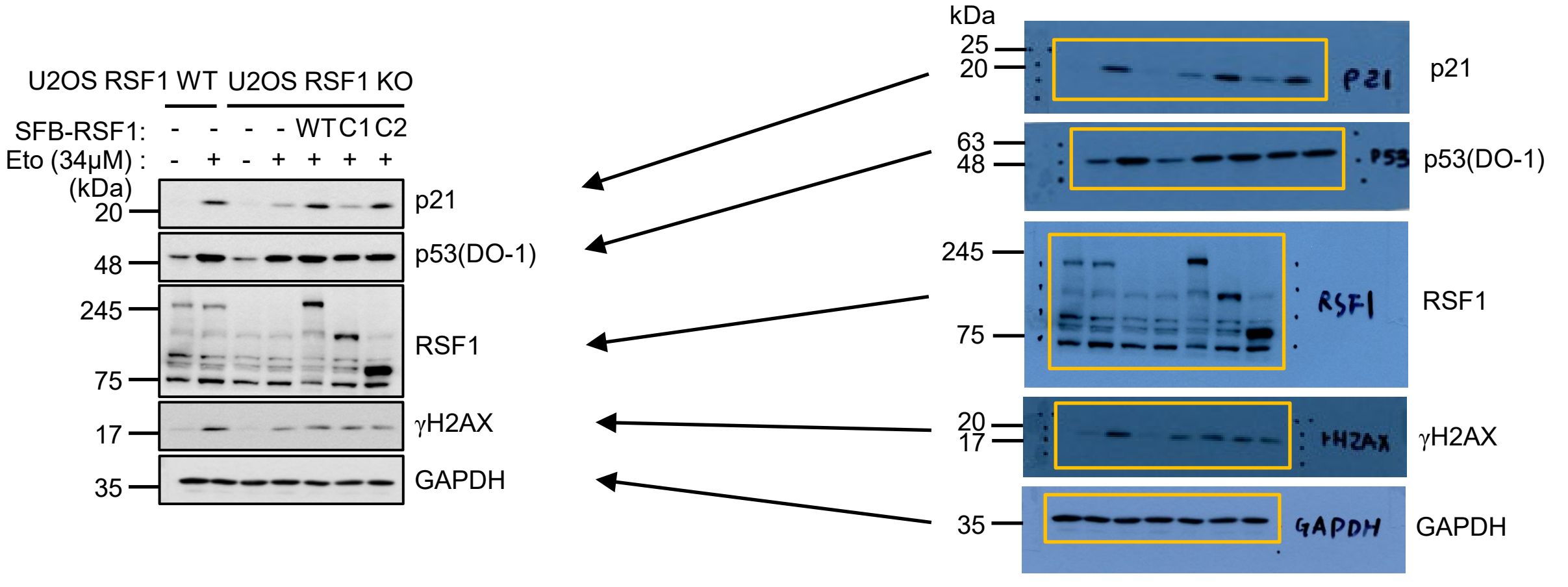

Figure S1D

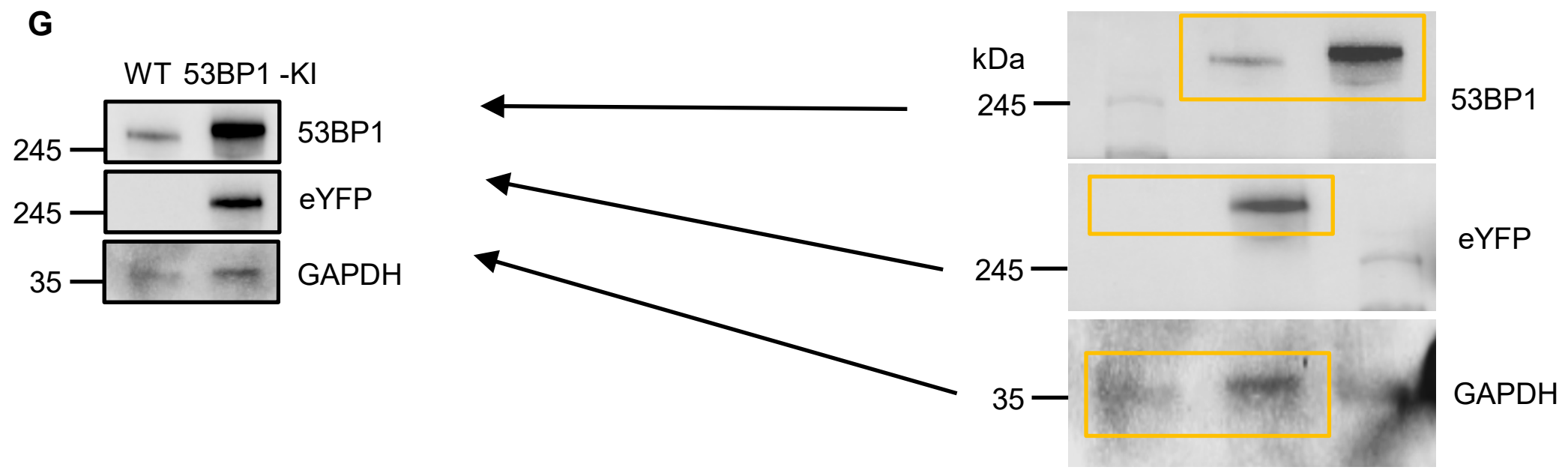

Figure S3A

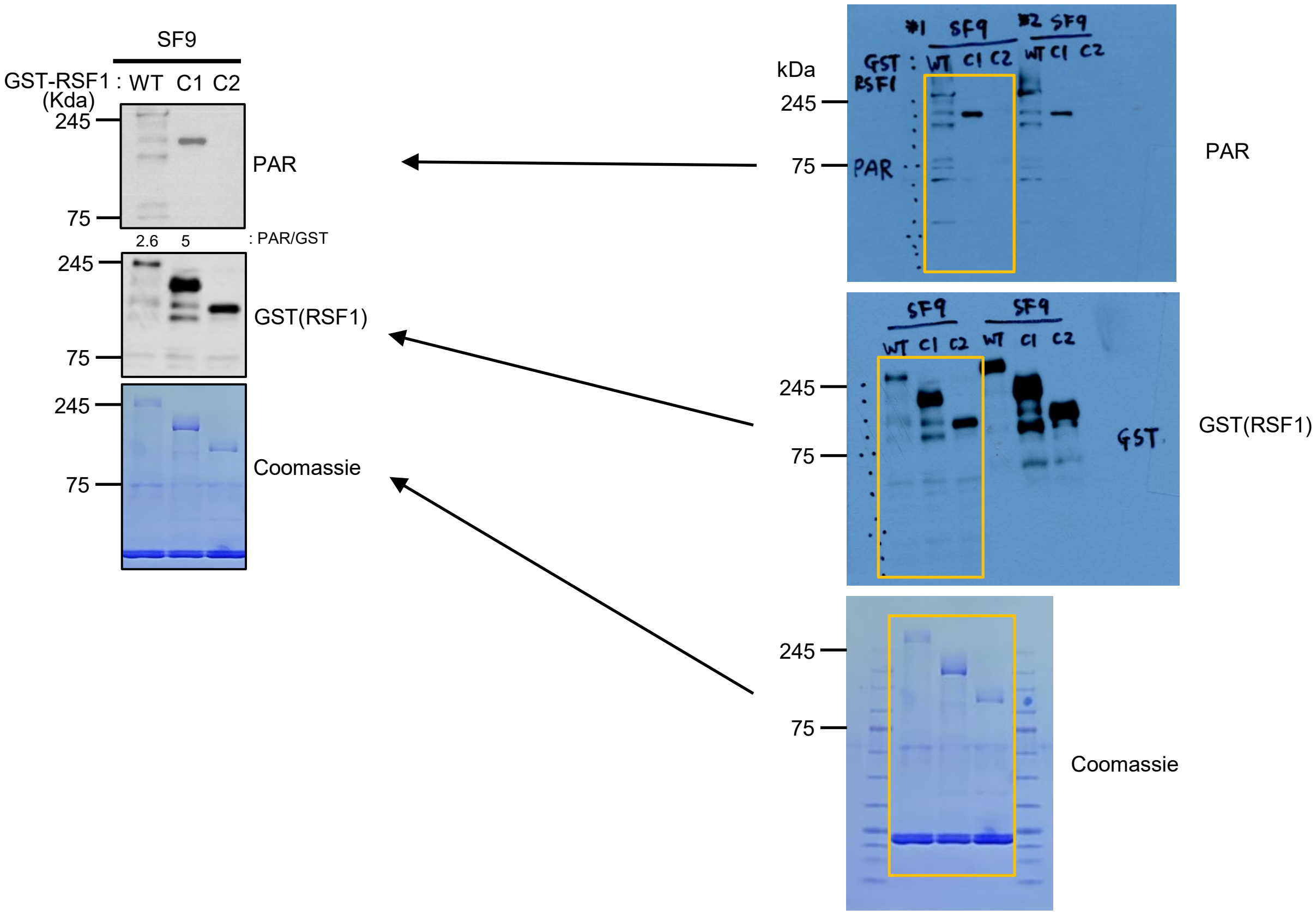

Figure S3B

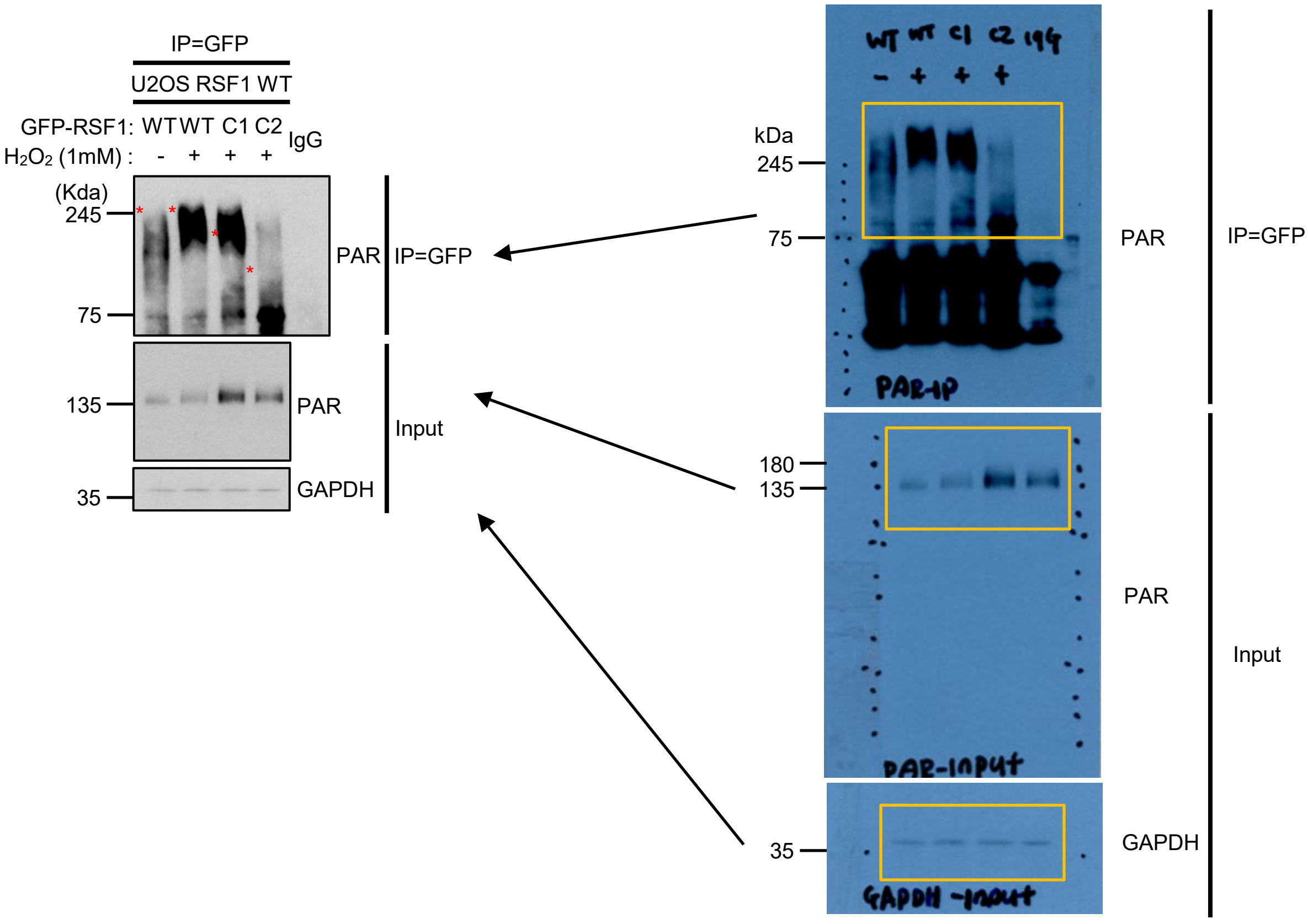

Figure S6B

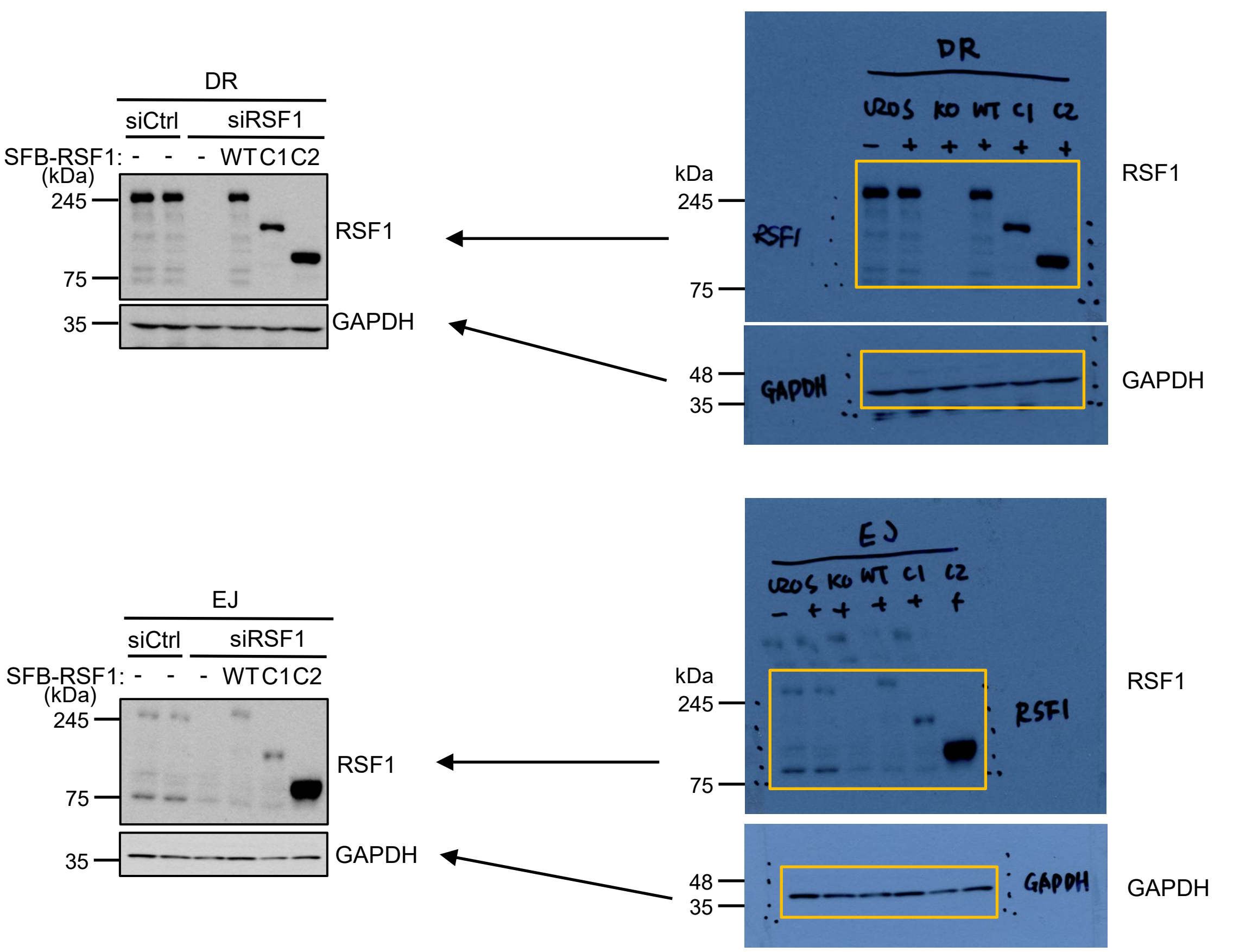

Supplement: Supplementary file 1 [file ADVS-9999-e77271-s001.pdf]
